# Chromogenic Properties of *p*-Pyridinium- and *p*-Viologen-Calixarenes and Their Cation-Sensing Abilities

**DOI:** 10.1021/acs.joc.1c01687

**Published:** 2021-09-01

**Authors:** Veronica Iuliano, Paolo Della Sala, Carmen Talotta, Luca Liguori, Giovanni Monaco, Ermelinda Tiberio, Carmine Gaeta, Placido Neri

**Affiliations:** Department of Chemistry and Biology “A. Zambelli”, University of Salerno, Via Giovanni Paolo II, 132, I-84084 Fisciano, Salerno, Italy

## Abstract

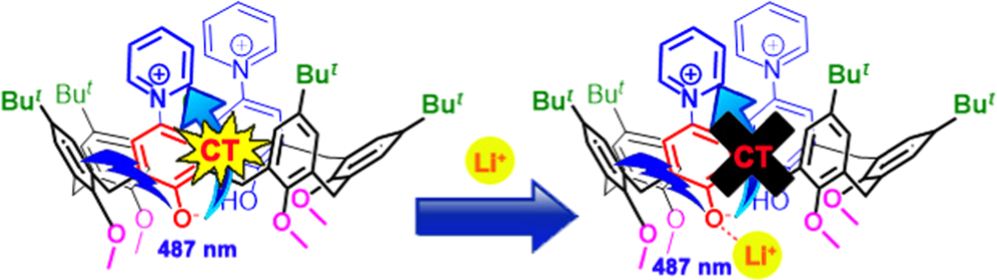

The synthesis of calix[4]- and -[6]arene derivatives **P6**(H)_2_^2+^·(Cl^–^)_2_, **V4**(H)_2_^4+^·(Cl^–^)_2_·(I^–^)_2_, and **V6**(H)_2_^4+^·(Cl^–^)_2_·(I^–^)_2_ bearing *N*-linked pyridinium (**P**) and viologen (**V**) units at the upper rim is described here. A rare example of an anionic conformational template is reported for *p*-pyridiniumcalix[6]arene **P6**(H)_2_^2+^, which adopts a 1,3,5-alternate conformation in the presence of chloride anions. Derivatives **P6**(H)_2_^2+^·(Cl^–^)_2_, **V6**(H)_2_^4+^·(Cl^–^)_2_·(I^–^)_2_, and **V4**(H)_2_^4+^·(Cl^–^)_2_·(I^–^)_2_ show a negative solvatochromism, while their UV–vis acid–base titration evidenced that upon addition of a base, new bands appear at 487, 583, and 686 nm, respectively, due to the formation of betainic monodeprotonated species **P6**(H)_1_^+^, **V6**(H)_1_^3+^, and **V4**(H)_1_^3+^. These new bands were attributable to the intramolecular charge-transfer (CT) transition from the phenoxide to the pyridinium or viologen moiety and were responsive to the presence of cations. In fact, the band at 487 nm of **P6**(H)_1_^+^ was quenched in the presence of a hard Li^+^ cation, and the color of its acetonitrile solution was changed from pink to colorless upon addition of LiI. Consequently, this derivative can be considered as a useful host for the recognition and sensing of lithium cations.

## Introduction

**Figure 1:**
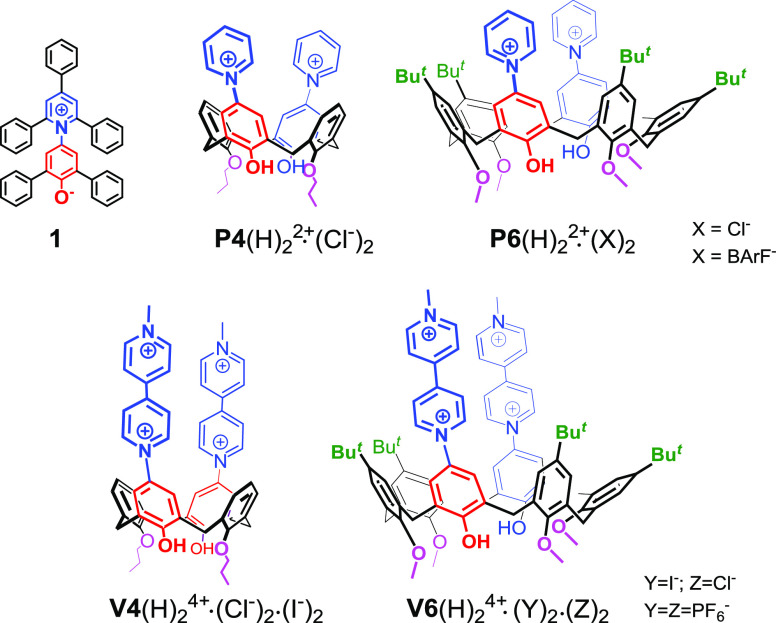
Chemical drawing of derivatives 1, **P4**(H)_2_^2+^·(Cl^–^)_2_, **P6**(H)_2_^2+^·(Cl^–^)_2_, **P6**(H)_2_^2+^·(BArF^–^)_2_, **V4**(H)_2_^4+^·(Cl^–^)_2_·(I^–^)_2_, **V6**(H)_2_^4+^·(Cl^–^)_2_·(I^–^)_2_, and **V6**(H)_2_^4+^·(PF_6_^–^)_4_ investigated in the present work (BArF = tetrakis[3,5-bis(trifluoromethyl)phenyl]borate).

Chromogenic molecules^1^ can respond to external stimuli by varying their optical properties. In recent years, much effort has been devoted to the design of chromogenic derivatives as supramolecular hosts for the sensing of cations and anions^2^ or biomedical applications.^3^ In addition, the study of chromogenic molecules with novel optical properties plays a crucial role in the development of high-performance chromogenic materials.^4^ Among chromogenic phenomena, solvatochromism^5^ is one of the most studied^6^ and consists in a change of absorption and/or emission spectra of a chromophore by changing the solvent polarity. It is a complex phenomenon in which the secondary interactions between a solvent and excited and ground states of a chromophore play a crucial role. Reichardt and co-workers^7^ reported the synthesis and the study of the chromogenic properties of derivatives incorporating a *N*-phenoxide pyridinium group (e.g., **1** in Figure 1). This *p*-pyridiniumphenoxide shows a betaine structure in which absorption arises from a charge-transfer band from the phenoxide donor group to the pyridinium acceptor.^7,8^ Interestingly, the betaine reported by Reichardt shows a negative solvatochromism in which a hypsochromic shift is experienced by increasing the polarity of the solvent. In this case, the dipole moment of the betaine decreases in the excited state when compared to the ground state; consequently, the ground state is energy-stabilized in polar solvents, which can interact with it by H-bonding and/or dipolar interactions.

In addition to solvatochromism, another noteworthy chromogenic property is halochromism, which was introduced for the first time in 1902 by Baeyer and Villiger.^8a^ This phenomenon is based on a color change of a molecule upon addition of an acid or base. Successively, Reichardt and co-workers^8b^ defined as trivial this form of halochromism and defined true halochromism as a color change of a dye solution upon addition of an electrolyte not accompanied by a chemical reaction. They suggested the term negative or positive true halochromism for a hypsochromic or bathochromic shift, respectively, of the UV–vis absorption band of a dissolved molecule on increasing the electrolyte concentration.

Calix[*n*]arenes^9^ are versatile macrocycles largely used in host–guest chemistry.^10^ Consequently, they have also been widely used to design chromogenic hosts^2,3,10^ for molecular recognition and sensing of cationic and/or anionic guests. Very recently, we have synthesized *p*-pyridiniumcalix[4]arene derivative **P4**(H)_2_^2+^·(Cl^–^)_2_^11^ (Figure 1) incorporating two pyridiniumphenoxide units into the calix[4]arene backbone. Derivative **P4**(H)_2_^2+^·(Cl^–^)_2_ was obtained by a Zincke reaction,^12^ which can be considered as a useful strategy for introducing pyridinium units at the upper rim of calixarene macrocycles. **P4**(H)_2_^2+^·(Cl^–^)_2_ shows a negative solvatochromism,^11^ and time-dependent density-functional theory (TD-DFT) quantum chemical calculations indicate that the species responsible for this phenomenon is the monodeprotonated betainic form, which is very abundant at the experimental neutral pH.^11^

Prompted by these results, we decided to extend our previous investigation by exploring other members of the calixarene family and their conjugation with pyridinium (**P**) and viologen (**V**) units (Figure 1). Thus, we report here the synthesis of derivatives **P6**(H)_2_^2+^·(Cl^–^)_2_, **V4**(H)_2_^4+^·(Cl^–^)_2_·(I^–^)_2_, and **V6**(H)_2_^4+^·(Cl^–^)_2_·(I^–^)_2_ (Figure 1) and their chromogenic and cation-sensing properties.

## Results and Discussion

### Synthesis and Conformational Properties of the Studied Macrocycles

**Scheme 1:**
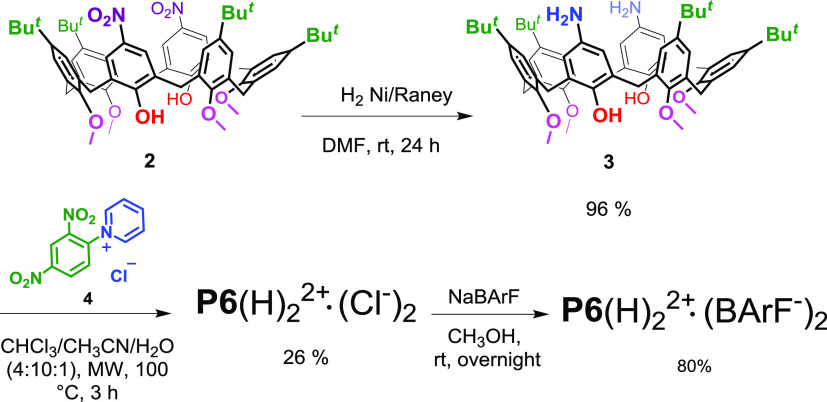
Synthesis of Derivatives **P6**(H)_2_^2+^·(Cl^–^)_2_ and **P6**(H)_2_^2+^·(BArF^–^)_2_

The synthesis of *p*-pyridiniumcalix[6]arene **P6**(H)_2_^2+^·(Cl^–^)_2_ is outlined in Scheme 1. Starting with the known distal dinitro-derivative **2**,^13^ its nitro groups were reduced with H_2_ in the presence of Raney nickel to give derivative **3** in high yield (96%). At this point, **3** and Zincke’s salt **4**(14) were reacted under microwave irradiation at 100 °C and using a mixture of CHCl_3_/CH_3_CN/H_2_O (4:10:1) as a solvent to give bis(*p*-pyridinium)calix[6]arene **P6**(H)_2_^2+^·(Cl^–^)_2_ in 26% yield.

**Figure 2:**
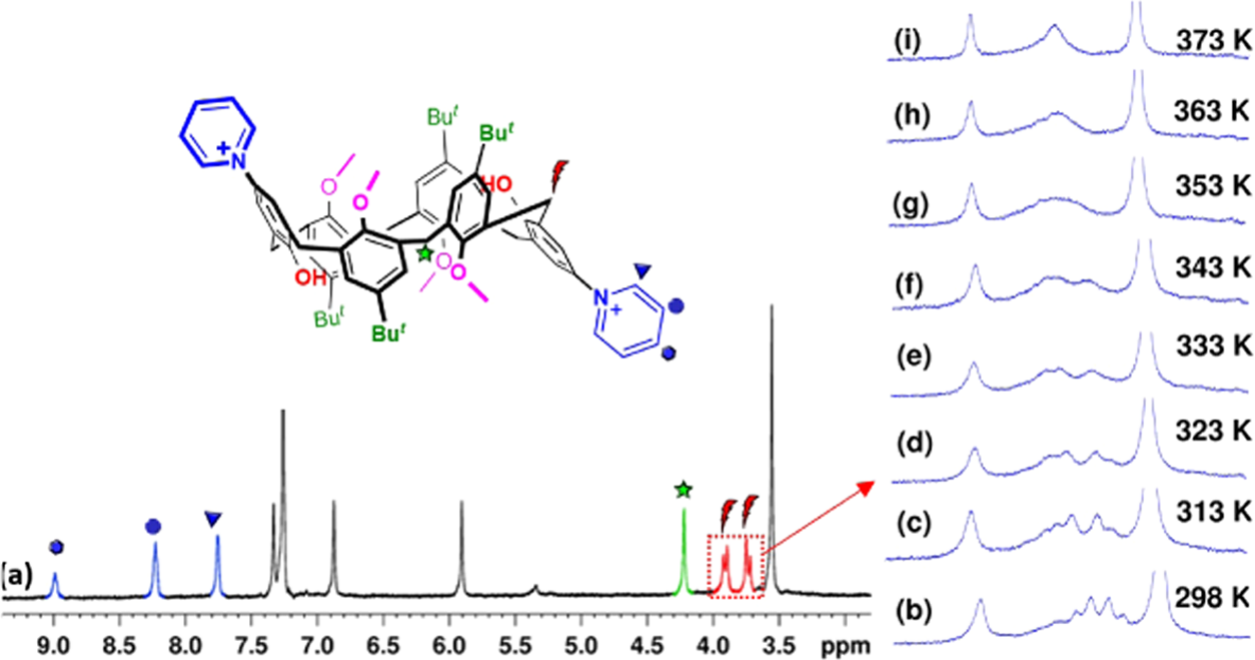
Portion of the ^1^H NMR spectrum of derivative **P6**(H)_2_^2+^·(Cl^–^)_2_ (CDCl_3_, 600 MHz, 298 K). (Inset on the right) Significant portions of the VT ^1^H NMR spectra of **P6**(H)_2_^2+^·(Cl^–^)_2_ (TCDE, 600 MHz);*T*_c_ = 353 K.

**Figure 3:**
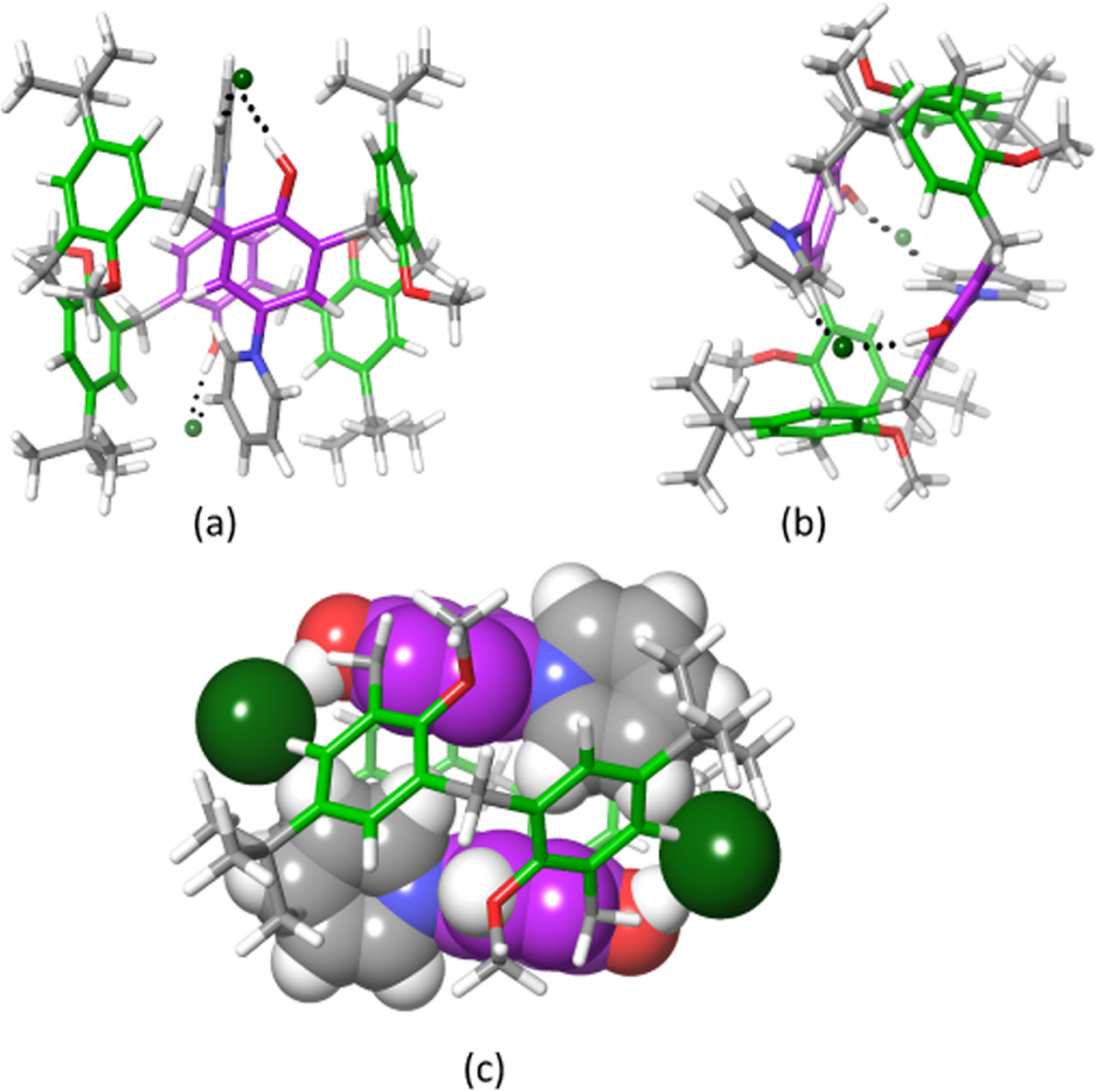
(a–c) Different views of the DFT-optimized structure of the **P6**(H)_2_^2+^·(Cl^–^)_2_ salt obtained at the B3LYP/6-31G(d,p) level of theory. The dashed lines indicate H-bonding interactions with chloride anions. (c) DFT-predicted templating mode of the chloride anions involves the pyridiniumphenol groups as a chelating motif toward the Cl^–^ anions, while the calix[6]arene skeleton adopts the 1,3,5-alternate conformation.

**P6**(H)_2_^2+^·(Cl^–^)_2_ was completely characterized by one-dimensional (1D) and two-dimensional (2D) nuclear magnetic resonance (NMR) studies and mass spectrometry. 1D and 2D NMR studies (Figures S5–S9) clearly indicated that the calix[6]arene backbone of **P6**(H)_2_^2+^ adopts a 1,3,5-alternate conformation (Figures 2 and 3), as evidenced by the presence of a singlet signal at 4.22 ppm attributable to the ArCH_2_Ar groups between two *anti*-oriented anisole rings^15^ (Figure 2) and an AB system at 3.88/3.72 ppm (*J* = 17.3 Hz) between *anti*-oriented anisole and *p*-pyridiniumphenol rings (Figure 2).^15^ Regarding the *p*-pyridinium group, three signals were found at 8.99, 8.23, and 7.76 ppm. VT ^1^H NMR study (Figures 2b–d and S14) of **P6**(H)_2_^2+^·(Cl^–^)_2_ clearly indicated that the calix[6]arene macrocycle experiences a conformational interconversion by means of rotation around the ArCH_2_Ar bonds. Thus, by increasing the temperature of a TCDE solution of **P6**(H)_2_^2+^·(Cl^–^)_2_, a coalescence of the ArCH_2_Ar AB system was detected in its ^1^H NMR spectrum at 353 K (Figure 2b–d). From these data, an energy barrier of 17.4 kcal/mol^16^ was calculated for the rotation around the ArCH_2_Ar bonds in **P6**(H)_2_^2+^·(Cl^–^)_2_.

**Figure 4:**
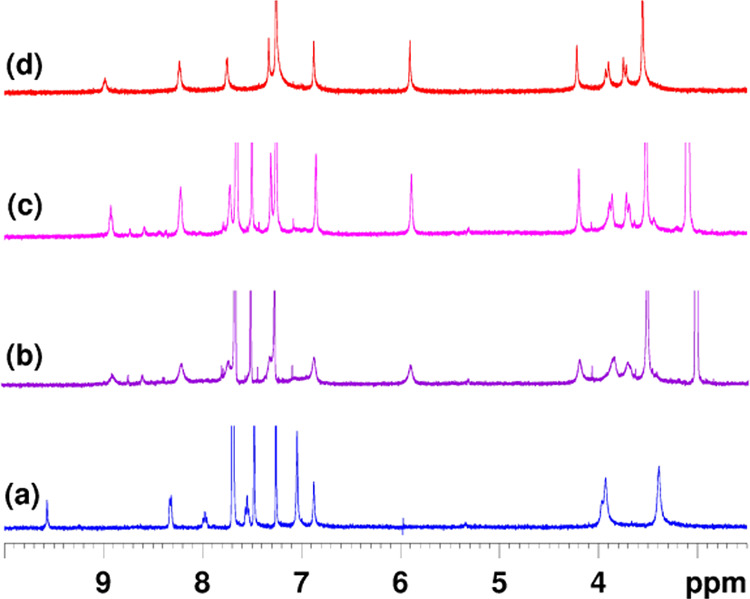
Significant portions of ^1^H NMR spectra (CDCl_3_, 600 MHz, 298 K) of (a) **P6**(H)_2_^2+^·(BArF^–^)_2_; (b) 1:1 mixture of **P6**(H)_2_^2+^·(BArF^–^)_2_ and tetrabutylammonium chloride; (c) 1:3 mixture of **P6**(H)_2_^2+^·(BArF^–^)_2_ and tetrabutylammonium chloride; and (d) **P6**(H)_2_^2+^·(Cl^–^)_2_.

Impressively, when the chloride anions of **P6**(H)_2_^2+^·(Cl^–^)_2_ were exchanged with BArF (tetrakis [3,5-bis(trifluoromethyl)phenyl]borate) anions,^17^ then the ^1^H NMR spectrum of the salt **P6**(H)_2_^2+^·(BArF^–^)_2_ at 298 K in CDCl_3_ loses the AB system and shows only a sharp singlet at 3.97 ppm for the ArCH_2_Ar groups (Figures 4a and S10). On lowering the temperature, a broadening of this methylene signal was observed and a coalescence was detected at 243 K (Figure S15). Analogously, a broadening and coalescence were observed for the aromatic signals of **P6**(H)_2_^2+^. These results indicated that below the coalescence temperature of 243 K the rotation around the ArCH_2_Ar bonds was slowed down and at 183 K a mixture of conformers of **P6**(H)_2_^2+^ was detected in its ^1^H NMR spectrum (Figures S14 and S15). From these data, an energy barrier of 10.8 kcal/mol^16^ was calculated for the conformational interconversion of **P6**(H)_2_^2+^·(BArF^–^)_2_, a value significantly lower than that calculated for the chloride salt **P6**(H)_2_^2+^·(Cl^–^)_2_.

These results clearly indicate that the chloride anion can act as a conformational template for bis(pyridinium)calix[6]arene dication **P6**(H)_2_^2+^ blocking its skeleton in the 1,3,5-alternate conformation (Figure 3). To gain insights into the reason for this amazing anionic template, we performed DFT calculations at the B3LYP/6-31G(d,p) level of theory.^18^ The DFT-optimized structure of **P6**(H)_2_^2+^·(Cl^–^)_2_ (Figure 3) showed a 1,3,5-alternate conformation for its calix[6]arene skeleton. The distal inverted *p*-pyridiniumphenol moieties (Figure 3) pointed in antiparallel orientation and chelated the two chloride anions by H-bonding and electrostatic interactions (N^+^···Cl^–^). In detail, the phenolic OH group engaged a H-bonding interaction with Cl^–^ at an O–H···Cl^–^ distance of 2.9 Å and an O–H···Cl^–^ angle of 167°. Furthermore, the chloride anion established a weak H-bonding interaction with the C(β)–H group on the distal pyridinium ring with a C(β)–H···Cl^–^ distance of 3.3 Å and a C(β)–H···Cl^–^ angle of 156°. This chelating motif of two pyridiniumphenol units toward each chloride anion (Figure 3c) plays a crucial role in the stabilization of the 1,3,5-alternate conformation of **P6**(H)_2_^2+^. Differently, in the **P6**(H)_2_^2+^·(BArF^–^)_2_ salt, the barfate anion forms a very loose ion pair in solution. As known, BArF^–^ is a weakly coordinating anion in which the negative charge is highly disperse, and literature data^19^ clearly indicate that BArF^–^ does not have any H-bonding acceptor ability. Consequently, in an organic solvent, ammonium cations and barfate anion give very loose ion pairs originating free “naked” organic cations.^17,19^

In conclusion, differently with respect to the chloride salt, when the bis(pyridinium)calix[6]arene dication **P6**(H)_2_^2+^ is associated with BArF^–^ anions, no H-bonding interactions can be established with the phenolic OH groups because of the poor coordinating abilities of this anion. To corroborate this conclusion, we performed a ^1^H NMR titration experiment (Figure 4) in which the tetrabutylammonium chloride salt was added to the solution of **P6**(H)_2_^2+^·(BArF^–^)_2_ in CDCl_3_. Upon addition of Cl^–^, changes in the ^1^H NMR spectrum of **P6**(H)_2_^2+^·(BArF^–^)_2_ became evident, and finally a singlet and an AB system for ArCH_2_Ar appeared (Figure 4c), attributable to the 1,3,5-alternate conformation of **P6**(H)_2_^2+^·(Cl^–^)_2_. Differently, the titration of **P6**(H)_2_^2+^·(BArF^–^)_2_ with tetrabutylammonium bromide or iodide did not change the ^1^H NMR signals of **P6**(H)_2_^2+^, a clear sign that less significant interactions occurred between the pyridiniumcalix[6]arene skeleton and bromide or iodide anions. To further corroborate the role of the chloride anion as a conformational template, we performed ^1^H NMR investigations in polar solvents such as CD_3_CN and CD_3_OD (Figure S44), which have great aptitude to disrupt ion pairs. In both the solvents, the ^1^H NMR spectrum of **P6**(H)_2_^2+^·(Cl^–^)_2_ showed the typical features of a high conformational mobility (Supporting Information). This result is indicative of the rupture of the **P6**(H)_2_^2+^·(Cl^–^)_2_ ion pair in polar solvents and consequently of the loss of the chloride template effect on the 1,3,5-alternate conformation of **P6**(H)_2_^2+^. Even though a cationic conformational template^19,20^ is widely described for calixarene macrocycles,^19,20^ this is a rare example of an anionic conformational template for this class of hosts.^21^

With these results in hand, we focused our attention on the synthesis of calixarenes bearing 4,4′-bipyridinium units at the upper rim. The known^22^ derivative **V4**(H)_2_^4+^ (Figure 1) bearing two viologen units at the upper rim of the calix[4]arene scaffold was obtained by following the procedure reported by Bucher and co-workers.^22^

**Scheme 2:**
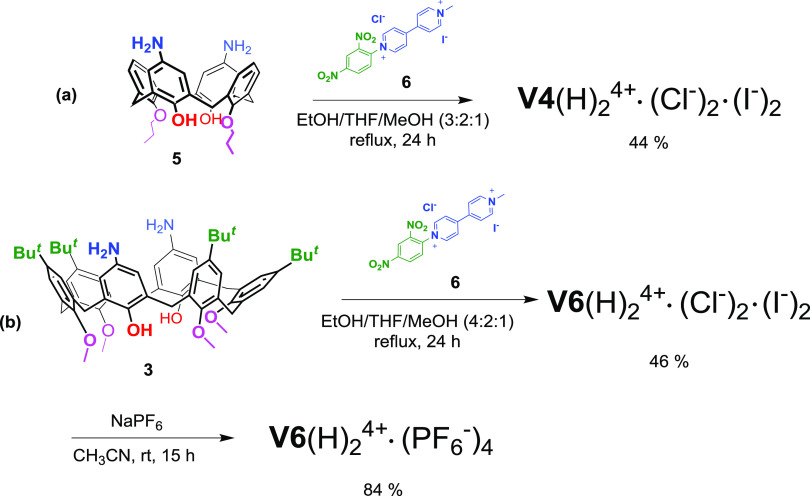
(a) Synthesis of Derivative **V4**(H)_2_^4+^·(Cl^–^)_2_·(I^–^)_2_and (b) Synthesis of Derivatives **V6**(H)_2_^4+^·(Cl^–^)_2_·(I^–^)_2_ and **V6**(H)_2_^4+^·(PF_6_^–^)_4_

In this work, the chloride/iodide salt **6**(23) (see Scheme 2a) was used for the Zincke reaction with diaminocalix[4]arene **5**(22) and, consequently, a chloride/iodide salt **V4**(H)_2_^4+^·(Cl^–^)_2_·(I^–^)_2_ was formed (Scheme 2a), which adopts a cone conformation in solution (Figure S16). Analogous conditions^22^ were employed for the synthesis of *p*-viologencalix[6]arene **V6**(H)_2_^4+^·(Cl^–^)_2_·(I^–^)_2_ bearing two 4,4′-bipyridinium units at the upper rim (Scheme 2b). Diaminocalix[6]arene **3** was reacted with an excess of dinitrophenyl-bipyridinium **6** in a mixture of ethanol/THF/methanol (4:2:1) at reflux for 24 h to afford **V6**(H)_2_^4+^·(Cl^–^)_2_·(I^–^)_2_ in 46% yield, after precipitation by water. At this point, anion exchange with NaPF_6_ afforded derivative **V6**(H)_2_^4+^·(PF_6_^–^)_4_ in 84% yield. **V6**(H)_2_^4+^·(PF_6_^–^)_4_ was completely characterized by 1D and 2D NMR studies and mass spectrometry (Figures S24–S27). The ^1^H NMR spectrum of **V6**(H)_2_^4+^·(PF_6_^–^)_4_ in CD_3_CN shows the presence of sharp singlets for ArCH_2_Ar groups indicative of a fast conformational interconversion of the calix[6]arene scaffold.

### Acid–Base UV–Vis Titrations of Calixarene Derivatives **P6**(H)_2_^2+^, **V4**(H)_2_^4+^, and **V6**(H)_2_^4+^

At this point, the absorption properties and the acid–base UV–vis titrations of *p*-pyridinium- and *p*-viologencalixarenes were investigated.^24^ Starting with **P6**(H)_2_^2+^·(Cl^–^)_2_, its absorption spectrum in acetonitrile shows a band at 326 nm, with a molar extinction coefficient ε_326_ = 12 171 M^–1^ cm^–1^ (Figure S30). Similarly, the corresponding barfate **P6**(H)_2_^2+^·(BArF^–^)_2_ shows an analogous absorption band at 326 nm (ε_326_ = 12 787 M^–1^ cm^–1^) attributable to the pyridiniumphenol unit (Figures S28 and S31). Barfate is a chromogenic anion that shows absorption bands in the UV–vis region of our interest, and for this reason, we decided to continue UV–vis studies on derivative **P6**(H)_2_^2+^·(Cl^–^)_2_.

**Figure 5:**
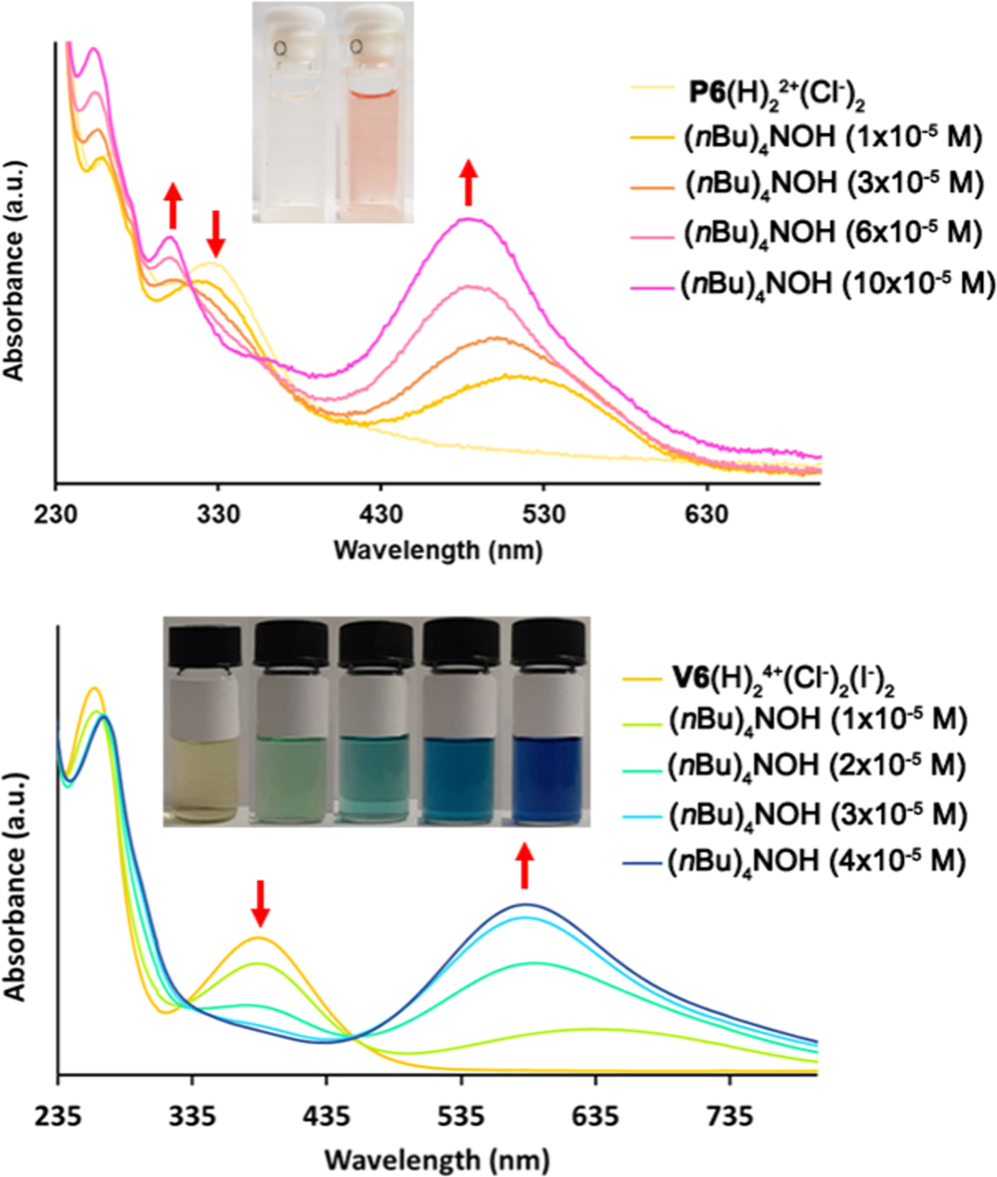
(Top) Color change of *p*-pyridiniumcalix[6]arene **P6**(H)_2_^2+^·(Cl^–^)_2_ upon addition of (*n*Bu)_4_NOH in acetonitrile (inset) and the UV–vis titration of **P6**(H)_2_^2+^·(Cl^–^)_2_ (6 × 10^–5^ M) with (*n*Bu)_4_NOH in acetonitrile. (Bottom) Color changes of *p*-viologencalix[6]arene **V6**(H)_2_^4+^·(Cl^–^)_2_·(I^–^)_2_ upon addition of (*n*Bu)_4_NOH in acetonitrile (inset) and the UV–vis titration of **V6**(H)_2_^4+^·(Cl^–^)_2_·(I^–^)_2_ (4 × 10^–5^ M) with (*n*Bu)_4_NOH in acetonitrile.

**Figure 6:**
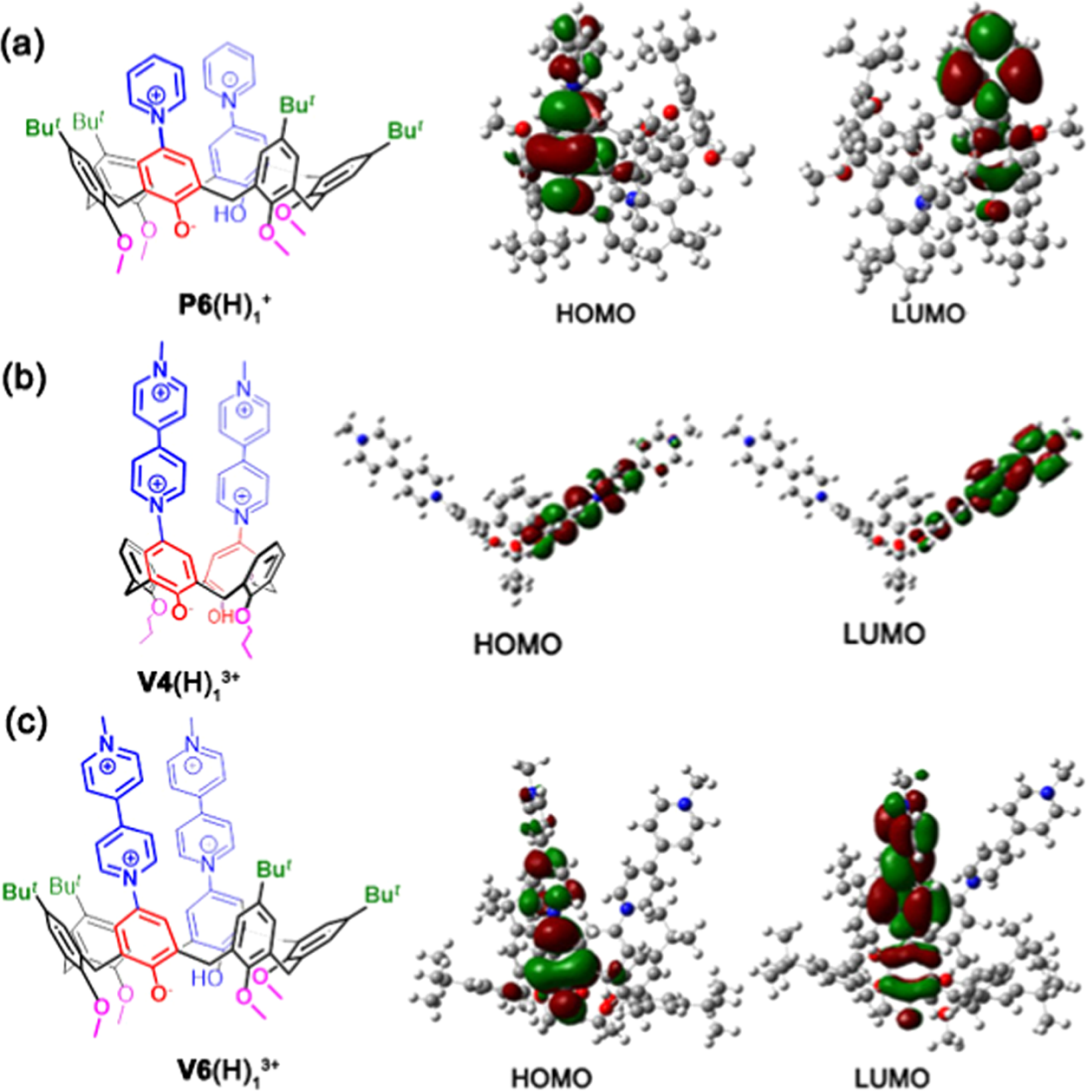
Isodensity surface plots of the frontier Kohn–Sham molecular orbitals of (a) betainic monocation **P6**(H)_1_^+^, (b) betainic trication **V4**(H)_1_^3+^, and (c) betainic trication **V6**(H)_1_^3+^.

Subsequently, the acid–base UV–vis titration of **P6**(H)_2_^2+^·(Cl^–^)_2_ was investigated. In Figure 5 (top), the UV–vis titration of **P6**(H)_2_^2+^·(Cl^–^)_2_ with (*n*Bu)_4_NOH in acetonitrile is reported. After addition of a base, the absorption band at 326 nm disappears and two new bands appear at 305 and 487 nm with an isosbestic point at 312 nm (Figure 5, top), attributable to a first deprotonation with formation of betainic monocation **P6**(H)_1_^+^. The band at 487 nm in the UV–vis spectrum of **P6**(H)_1_^+^ was simulated by DFT calculations at the TD-CAM-B3LYP/6-31G(d,p) level of theory.^25^ An optical transition was computed at 437 nm (Figures S37 and S38) starting by the DFT-optimized structure of **P6**(H)_1_^+^. The corresponding highest occupied molecular orbital (HOMO)/lowest unoccupied molecular orbital (LUMO) for this transition (S1 ← S0) is reported in Figure 6 and clearly suggests an intramolecular charge-transfer (CT) transition from the phenoxide to the pyridinium moiety (Figure 6a).^25^ The formation of the betainic monocation **P6**(H)_1_^+^ was studied also by a ^1^H NMR titration experiment and 2D correlation spectroscopy (COSY) (Supporting Information). In detail, the CD_3_CN solution of **P6**(H)_2_^2+^·(Cl^–^)_2_ was titrated with (*n*Bu)_4_NOH, and the formation of **P6**(H)_1_^+^ was confirmed by the upfield shift of the signals of the pyridinium units of about 0.5–1.0 ppm (Figures S45–S47). This result confirmed the charge transfer from the phenoxide to the pyridinium moiety already discussed by UV–vis experiments and DFT calculations (Figure 6a).

In a similar way, a study was conducted on *p*-viologencalix[4]arene **V4**(H)_2_^4+^·(Cl^–^)_2_·(I^–^)_2_ whose yellow solution in acetonitrile shows an absorption band at 367 nm (ε_367_ = 19 326 M^–1^ cm^–1^) (Figures S32 and S35). With the gradual addition of (*n*Bu)_4_NOH, a color change of the solution from yellow to green was observed attributable to the formation of the monodeprotonated betainic form **V4**(H)_1_^3+^. Consequently, the original absorption band at 367 nm disappeared while a new band at 686 nm (Figure S34) emerged. DFT calculations at the TD-CAM-B3LYP/6-31G(d,p) level of theory predicted for betainic trication **V4**(H)_1_^3+^ a S1 ← S0 transition at 594 nm (experimental: 686 nm) in which the involved HOMOs/LUMOs clearly indicated an intramolecular charge-transfer (CT) transition from the phenolate to the viologen unit (Figures 6b, S39, and S40).

A further study on *p*-viologencalix[6]arene **V6**(H)_2_^4+^·(Cl^–^)_2_·(I^–^)_2_ (Figure 5, bottom) evidenced an absorption band at 386 nm (ε_385_ = 18 975 M^–1^ cm^–1^, Figure S33). Similarly, the corresponding hexafluorophosphate **V6**(H)_2_^4+^·(PF_6_^–^)_4_ gave no substantial variation in the absorption spectrum confirming that the UV–vis properties (ε_385_ = 17 324 M^–1^ cm^–1^, Figure S34) are not influenced by the anion (Figure S29). When an acetonitrile solution of **V6**(H)_2_^4+^·(Cl^–^)_2_·(I^–^)_2_ was titrated with (*n*Bu)_4_NOH, a color change from yellow to blue (inset in Figure 5, bottom) was observed. Consequently, the original absorption band at 386 nm disappeared, while a new band appeared at 583 nm, with an isosbestic point at 459 nm, attributable to a first deprotonation with formation of betainic trication **V6**(H)_1_^3+^ (Figure 5, bottom). DFT calculations at the TD-CAM-B3LYP/6-31G(d,p) level of theory predicted for **V6**(H)_2_^3+^ a S1 ← S0 transition at 607 nm (experimental: 583 nm) in which the involved HOMOs/LUMOs clearly indicated an intramolecular charge-transfer (CT) transition from the phenoxide to the viologen unit (Figures 6c, S41, and S42).

### Solvatochromic Properties of Betainic Calixarenes **P6**(H)_1_^+^, **V4**(H)_1_^3+^, and **V6**(H)_1_^3+^

**Figure 7:**
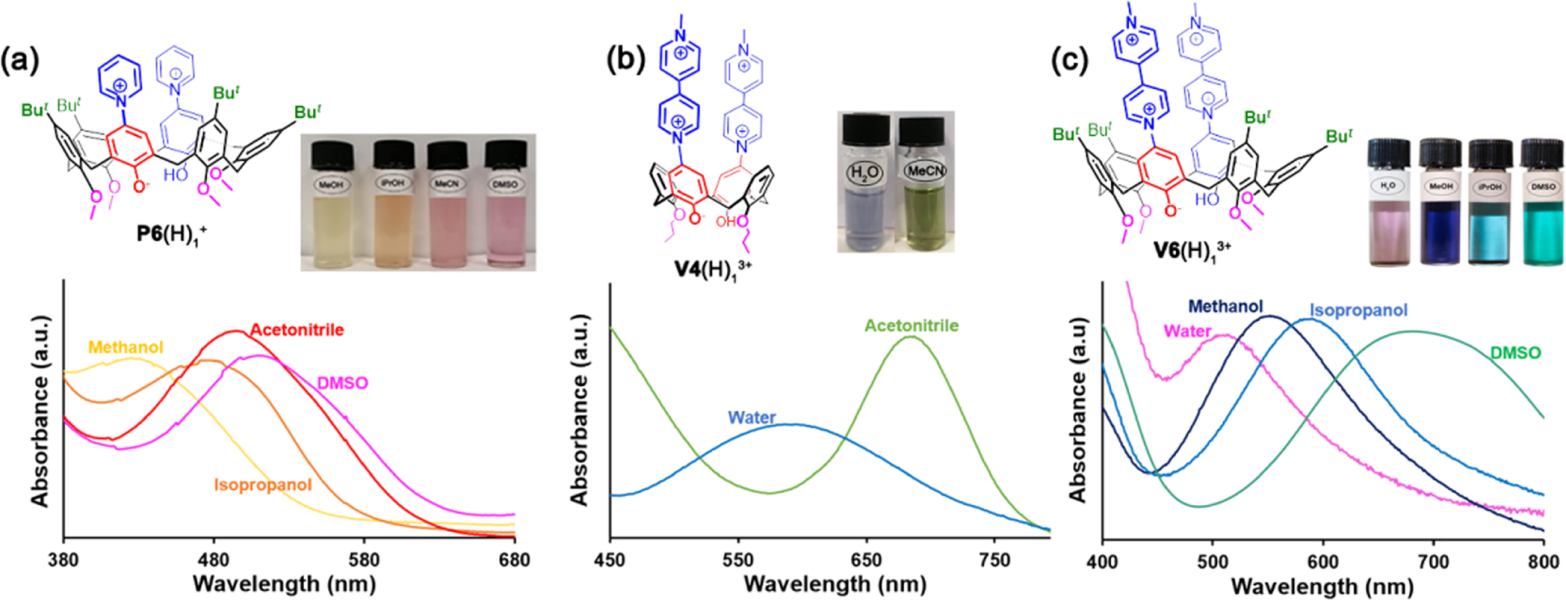
Solvatochromic properties of derivatives (a) **P6**(H)_1_^+^, (b) **V4**(H)_1_^3+^, and (c) **V6**(H)_1_^3+^ upon dissolution in solvents with different polarity and H-bonding donor abilities.

In accord with data previously reported by us for calix[4]arene derivative **P4**(H)_2_^2+^·(Cl^–^)_2_,^11^*p*-pyridiniumcalix[6]arene **P6**(H)_2_^2+^·(Cl^–^)_2_ also showed a negative solvatochromism. The UV–vis spectrum of betainic monocation **P6**(H)_1_^+^ in dimethyl sulfoxide (DMSO) shows a band at λ = 515 nm (Figure 7a). When the solvent was changed to methanol, this band underwent a significant blue shift to 432 nm (Figure 7a).

**Figure 8:**
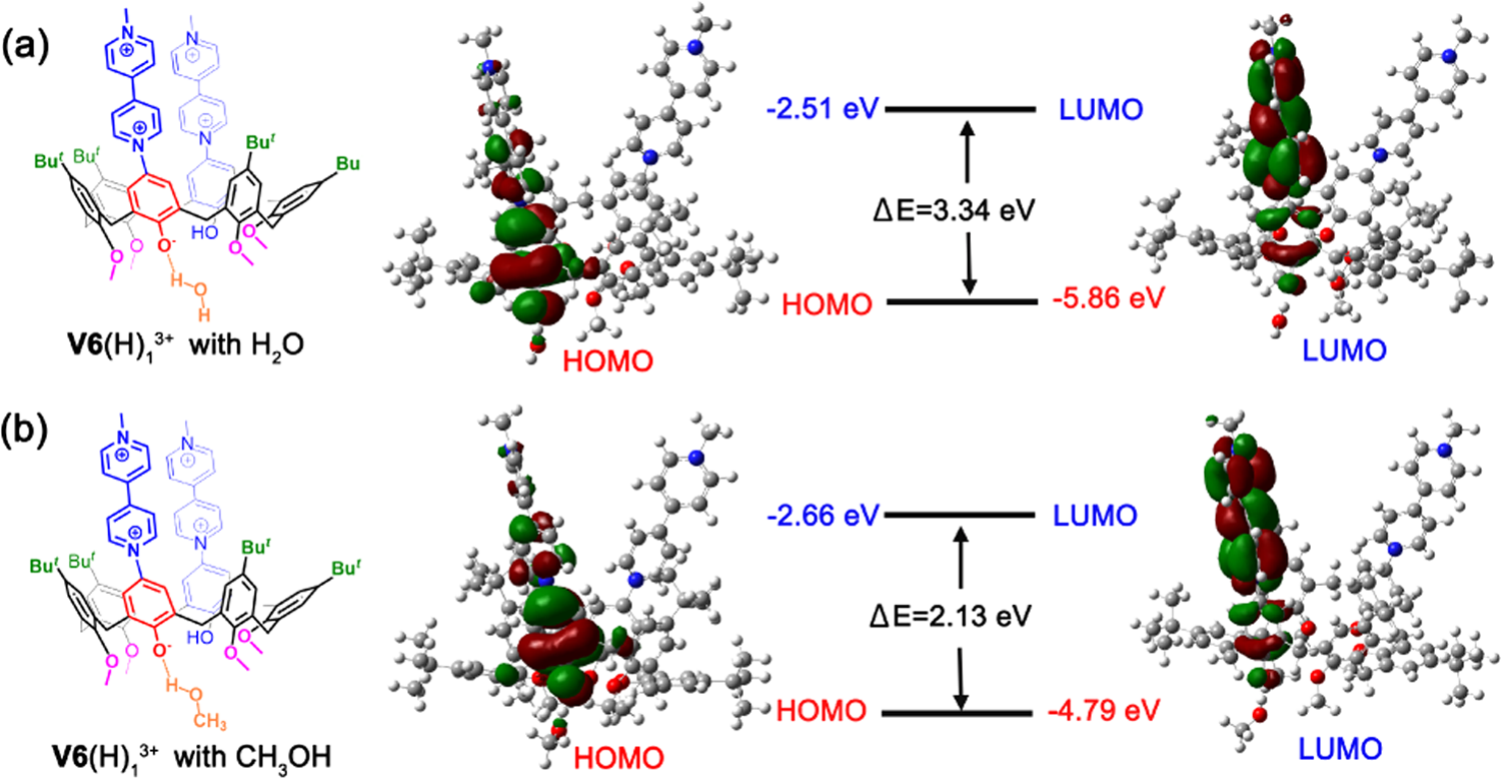
Isodensity surface plots of the frontier Kohn–Sham HOMOs/LUMOs of monodeprotonated betainic form **V6**(H)_1_^3+^ H-bonded to (a) the water molecule and (b) the methanol molecule.

Similar solvatochromic studies on betainic trication **V4**(H)_1_^3+^ evidenced a color change from green in acetonitrile to blue in water (Figure 7b). This corresponds to a blue shift of the absorption band of **V4**(H)_1_^3+^ (686 → 585 nm) as the polarity of the solvent increases (CH_3_CN → H_2_O) (Figure 7b), in accord with a negative solvatochromism. *p*-Viologencalix[6]arene betainic trication **V6**(H)^3+^ shows a band at λ = 680 nm in DMSO, which experienced a hypsochromic shift at 580, 550, and 510 nm in isopropanol, methanol, and water, respectively (Figure 7c). Thus, also in this case, a negative solvatochromism is observed, with a corresponding color change from green to pink (Figure 7c). The observed solvatochromism was studied by DFT calculations at the TD-CAM-B3LYP/6-31G(d,p) level of theory by reproducing the UV–vis spectra of **V6**(H)^3+^. Its simulated UV–vis spectrum shows an absorption band at 607 nm (Figure S42). At this point, we calculated the HOMO/LUMO transition for **V6**(H)^3+^ in which a methanol molecule was H-bonded to its phenoxide group The simulated band was shifted at 563 nm (Figure S43), and the formation of the ArO^–^···HOCH_3_ H-bonding interaction (Figure 8b) induced a stabilization of the HOMO (Figure 8b) of the low-energy excitation (S1 ← S0). Similarly, when a water molecule was H-bonded to the phenoxide group, the simulated absorption band was shifted at 553 nm (Figure S43). In this case, the formation of an ArO^–^···HOH hydrogen bond between the water molecule and the phenoxide group induced a further stabilization of the HOMO (Figure 8a). These results clearly suggested that the ground state of betainic trication **V6**(H)_1_^3+^ is energy-stabilized in polar solvents, such as methanol and water, by H-bonding interactions (Figure 7c).

Finally, calculation of the HOMO–LUMO energy difference associated with the S1 ← S0 transition for **V6**(H)_1_^3+^ indicated a Δ*E* value of 3.34 eV in the presence of a water molecule (Figure 8a), which is significantly higher than that calculated in the presence of a methanol molecule (Δ*E* = 2.13 eV) (Figure 8b), in agreement with the experimentally observed negative solvatochromism (Figure 7c).

### Cation Sensing with Betainic Calixarenes **P6**(H)_1_^+^, **V4**(H)_1_^3+^, and **V6**(H)_1_^3+^

Finally, the so-called true halochromism, as defined by Reichardt and co-workers,^8b^ was also studied for betainic *p*-pyridinium- and *p*-viologen-calixarenes. The UV–vis band of pyridiniumphenoxide derivatives is generated by an intramolecular charge-transfer (CT) transition from the phenoxide to the pyridinium moiety. Consequently, by adding cations to the solutions of betainic **P6**(H)_1_^+^, **V4**(H)_1_^3+^, and **V6**(H)_1_^3+^, a change of this CT band is expected because of the possible O^–^···M^+^ interactions.

**Figure 9:**
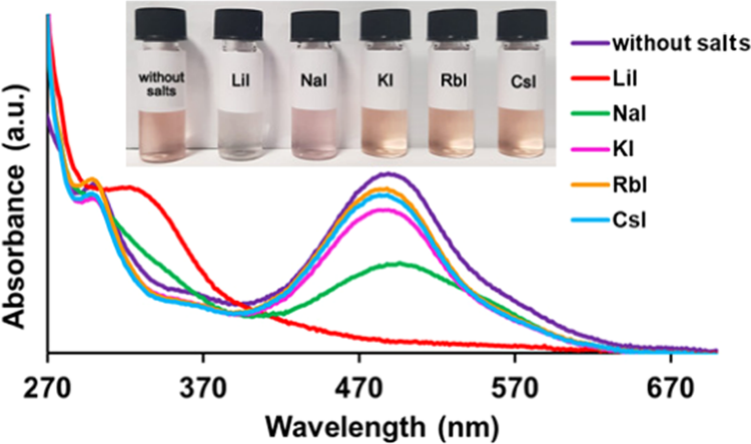
UV–vis spectra of betainic monocation P6(H)_1_^+^ in acetonitrile without a salt (purple), with 10 equiv of LiI (red), with 10 equiv of NaI (green), with 10 equiv of KI (pink), with 10 equiv of RbI (orange), and with 10 equiv of CsI (blue).

Five different alkaline salts were tested with betainic monocation **P6**(H)_1_^+^, namely, LiI, NaI, KI, RbI, and CsI. A chromatic response was observed with a consequent variation of the absorption spectrum (Figure 9).

In detail, a lowering of the intensity of the absorption band at 487 nm of **P6**(H)_1_^+^ was observed, which is correlated with the strength of O^–^···M^+^ association. Thus, the smaller cations Na^+^ and Li^+^ give rise to a stronger lowering of the band at 487 nm with respect to the other bigger alkaline cations. Upon addition of LiI, the acetonitrile solution of **P6**(H)_1_^+^ changed from pink to colorless and the CT band at 487 nm was completely quenched (Figure 9).

Interestingly, the affinities of **P6**(H)_1_^+^ toward Li^+^ and Na^+^ cations were evaluated by determining their association constants through Benesi–Hildebrand plots of UV–vis data (Supporting Information). The association constant for the formation of the Li^+^@**P6**(H)_1_^+^ complex was found to be 1.7±0.2 × 10^3^ M^–1^ (Supporting Information). This value is higher than that measured for the formation of the Na^+^@**P6**(H)_1_^+^ complex of 6.5±0.2 × 10^2^ M^–1^, and consequently, a selectivity ratio S = Li^+^/Na^+^ of 2.6 was calculated for **P6**(H)_1_^+^.

On the other hand, when betainic *p*-viologen-calixarene trications **V4**(H)_1_^3+^ and **V6**(H)_1_^3+^ were tested with the above alkaline salts, no chromatic response was observed (Figure S36). Probably, in this case, the stronger electron-withdrawing effect of the doubly charged viologen unit on the phenoxide group weakens the O^–^···M^+^ interactions.

## Conclusions

In conclusion, we have here reported the synthesis of calix[4]- and -[6]arene derivatives *N*-linked with pyridinium and viologen units at the upper rim [**P6**(H)_2_^2+^·(Cl^–^)_2_, **V4**(H)_2_^4+^·(Cl^–^)_2_·(I^–^)_2_, and **V6**(H)_2_^4+^·(Cl^–^)_2_·(I^–^)_2_] by coupling an appropriate Zincke’s salt to the corresponding diaminocalixarene. The conformational properties of *p*-pyridiniumcalix[6]arene **P6**(H)_2_^2+^ in the presence of chloride and barfate counter anions were studied by ^1^H VT NMR and rationalized by DFT calculations. A peculiar anion templation was observed on the conformation of the **P6**(H)_2_^2+^ skeleton: the chloride anion is able to template the 1,3,5-alternate conformation of the calix[6]arene macrocycle through H-bonding and electrostatic interactions. An energy barrier of 17.4 kcal/mol was calculated by VT NMR studies for the conformational interconversion of **P6**(H)_2_^2+^·(Cl^–^)_2_ by rotation around the ArCH_2_Ar bonds. This barrier dramatically decreases at 10.8 kcal/mol when the noncoordinating barfate anion is associated with **P6**(H)_2_^2+^. UV–vis acid–base titrations evidence that upon addition of a base, a new band appears due to the formation of betainic monodeprotonated species **P6**(H)_1_^+^, **V4**(H)_1_^3+^, and **V6**(H)_1_^3+^ at 487, 686, and 583 nm, respectively. DFT calculations indicate that these new bands are attributable to intramolecular charge-transfer (CT) transitions from the phenoxide to the pyridinium moiety. The band at 487 nm of **P6**(H)_1_^+^ is selectively quenched in the presence of a hard Li^+^ cation by virtue of a strong O^–^···Li^+^ interaction, which switches off the CT transition. Thus, upon addition of LiI, the color of the acetonitrile solution of **P6**(H)_1_^+^ changes from pink to colorless. Regarding *p*-viologencalix[4]- and -[6]arene **V4**(H)_1_^3+^ and **V6**(H)_1_^3+^, the respective bands at 686 and 583 nm are insensible to the presence of Li^+^. Probably, in this case, the stronger electron-withdrawing effect of the doubly charged viologen unit on the phenoxide group weakens the O^–^···M^+^ interactions. Betainic monodeprotonated species **P6**(H)_1_^+^, **V4**(H)_1_^3+^, and **V6**(H)_1_^3+^ show a negative solvatochromism. This behavior has been rationalized by DFT calculations, which indicate an energy stabilization of the ground state in polar solvents, such as methanol and water, by H-bonding interactions. We believe that the results here described could pave the way for the design of novel chromogenic supramolecular hosts based on pyridinium or viologen units for selective molecular recognition and sensing.

## Experimental Section

### General Information

Anhydrous reactions were conducted under an inert atmosphere (nitrogen) using dry solvents. Commercial reagents were purchased from Merck and TCI Chemicals and were used without further purification. The reactions were controlled by thin-layer chromatography (TLC) with Merck plates coated with silica gel (0.25 mm) and fluorescence indicator UV_254_ and visualized using UV light and nebulization with an indicator solution of H_2_SO_4_–Ce(SO_4_)_2_. For reactions that require heating, the heat source used was oil bath. The reaction temperatures were measured externally using electronic thermometers. The reaction products were purified by Macherey–Nagel silica gel chromatography (60, 70-230 mesh). The syntheses of derivatives **2**,^13^**4**,^14^**5**,^26^ and **6**(23) have been reported in the literature. NMR spectra were recorded on a Bruker Avance-600 spectrometer [600 (^1^H) and 150 MHz (^13^C)] and a Bruker Avance-400 spectrometer [400 (^1^H) and 100 MHz (^13^C)]. Chemical shifts are reported relative to the residual solvent peak (CHCl_3_: δ 7.26, CDCl_3_: δ 77.16; CH_3_CN: δ 1.94, CD_3_CN: δ 1.32, 118.26; CH_3_OH: δ 3.31, CD_3_OD: δ 49.00).^27^ Standard pulse programs, provided by the manufacturer, were used for 2D NMR experiments. HR MALDI mass spectra were recorded on a Bruker Solarix FT-ICR mass spectrometer equipped with a 7T magnet. The samples recorded in MALDI were prepared by mixing 10 μL of analyte in chloroform or methanol (1 mg/mL) with 10 μL of a solution of 2,5-dihydroxybenzoic acid (10 mg/mL in methanol). The mass spectra were calibrated externally, and a linear calibration was applied. Optical absorption spectra were measured on a Cary 50 UV–vis spectrophotometer from Varian. The extinction coefficients of derivatives were calculated by measuring the slope of the Lambert–Beer plot, and the data were analyzed by linear regression analysis. Details of DFT calculations are reported in the Supporting Information, page S34.

### Synthesis of Derivative **3**

Derivative **2** (130 mg, 0.129 mmol) was dissolved in 200 mL of DMF. Subsequently, Raney Ni (previously activated) was added to the reaction mixture. Finally, vacuum/H_2_ cycles were carried out. The suspension was stirred for 24 h at room temperature. Afterward, the reaction mixture was filtered on celite, and the solvent was evaporated. Derivative **3** was obtained in 96% yield as an amorphous pink solid (117 mg, 0.124 mmol). ^1^H NMR (CDCl_3_, 300 MHz, 298 K): δ 7.85 (s, −O*H*, 2H), 7.03 (overlapped, Ar*H*, 8H), 6.09 (s, Ar*H*, 4H), 3.96 (s, −ArC*H*_2_Ar-, 4H), 3.81 (s, −ArC*H*_2_Ar–, 8H), 3.18 (s, −OC*H*_3_, 12H), 1.21 (s, −C(C*H*_3_)_3_, 36H). ^13^C{^1^H} NMR (CDCl_3_, 100 MHz, 298 K): δ 153.7, 147.0, 145.2, 138.9, 134.5, 132.4, 129.3, 127.0, 126.7, 115.3, 61.7, 34.6, 31.8, 31.8, 31.3, 30.1; HRMS: *m*/*z* [M + H]^+^ calcd for C_62_H_79_N_2_O_6_^+^ 947.5933, found = 947.5971; [M + Na]^+^ calcd for C_62_H_78_N_2_NaO_6_^+^ 969.5752, found = 969.5818, [M + K]^+^ calcd for C_62_H_78_KN_2_O_6_^+^ 985.5491, found = 985.5531.

### Synthesis of Derivative **P6**(H)_2_^2+^·(Cl^–^)_2_

Derivative **3** (31 mg, 0.033 mmol) and Zincke’s salt **4** (64 mg and 0.26 mmol) were dissolved in a mixture of chloroform, acetonitrile, and water (4/10/1, 15 mL). The solution was stirred for 3 h under microwave irradiation (300 W) at 100°C. After cooling, the solvent was evaporated. The raw product was purified by a chromatography column using as eluents a mixture of CHCl_3_/CH_3_OH (8/2). An amorphous pink solid was obtained in 26% yield (10 mg, 0.009 mmol). ^1^H NMR (CDCl_3_, 400 MHz, 298 K): δ 8.99 (br, py-*H*, 2H), 8.23 (br, py-*H*, 4H), 7.76 (br, py-*H*, 4H), 7.33 (s, Ar*H*, 4H), 6.88 (s, Ar*H*, 4H), 5.91 (s, Ar*H*, 4H), 4.22 (s, −ArC*H*_2_Ar–, 4H), 3.90 and 3.73 (AX system, *J* = 15.9 Hz, −ArC*H*_2_Ar–, 8H), 3.56 (s, −OC*H*_3_, 12H), 1.25 (s, −C(C*H*_3_)_3_, 36H). ^13^C{^1^H} NMR (CDCl_3_, 100 MHz, 298 K): δ 156.1, 155.0, 147.9, 147.4, 140.4, 132.7, 132.3, 130.7, 128.2, 127.4, 126.8, 119.7, 60.0, 34.4, 31.6, 29.8. HRMS: *m*/*z* [M – H]^+^ calcd for C_72_H_83_N_2_O_6_^+^ 1071.6246; found = 1071.6232.

### Synthesis of Derivative **P6**(H)_2_^2+^·(BArF^–^)_2_

**P6**(H)_2_^2+^·(Cl^–^)_2_ (41 mg, 0.036 mmol) was dissolved in 10 mL of methanol, and Na(BArF) (63 mg, 0.072 mmol) was added. The mixture was stirred overnight at room temperature. Subsequently, the solvent was evaporated, and the crude product was triturated with water (10 mL), filtered, and dried under vacuum to give **P6**(H)_2_^2+^·(BArF^–^)_2_ in 80% yield (80 mg, 0.029 mmol). ^1^H NMR (CDCl_3_, 400 MHz, 298 K): δ 9.57 (s, −O*H*, 2H) 8.33 (d, *J* = 6.4 Hz, py-*H*, 4H), 7.98 (t, *J* = 6.4 Hz, py-*H*, 2H), 7.69 (s, BArF-*H*, 16H), 7.55 (t, *J* = 6.4 Hz, py-*H*, 4H), 7.48 (s, BArF-*H*, 8H), 7.04 (s, Ar*H*, 8H), 6.88 (s, Ar*H*, 4H), 3.96–3.93 (overlapped, ArC*H*_2_Ar, 12H), 3.37 (s, −OC*H*_3_, 12H), 1.16 (s, −C(C*H*_3_)_3_, 36H). ^13^C{^1^H} NMR (CDCl_3_, 100 MHz, 298 K): δ 162.1, 161.4, 148.2, 145.4, 143.1, 134.9, 134.3, 134.0, 131.6, 130.1, 129.5, 129.3, 129.1, 128.9, 128.4, 128.0, 127.3, 126.3, 125.5, 123.7, 122.1, 121.9, 117.7, 61.5, 34.4, 31.8, 31.3, 29.9. HRMS: *m*/*z* [M – H]^+^ calcd for C_72_H_83_N_2_O_6_^+^ 1071.6246; found = 1071.6264; [M + BArF]^+^ calcd for C_104_H_96_BF_24_N_2_O_6_^+^ 1935.6973; found = 1935.6895.

### Synthesis of Derivative **V4**(H)_2_^4+^·(Cl^–^)_2_·(I^–^)_2_

Derivative **5**(26) (50 mg, 0.093 mmol) and Zincke’s salt **6** (279 mg, 0.557 mmol) were dissolved in a mixture of ethanol/methanol/tetrahydrofuran (3:1:2, 60 mL). The solution was stirred for 24 h at 70 °C. After cooling the solvent was evaporated. The crude was purified by precipitation from water to give **V4**(H)_2_^4+^·(Cl^–^)_2_·(I^–^)_2_ as a pink amorphous solid in 44% yield (48 mg, 0.041 mmol). ^1^H NMR (600 MHz, CD_3_CN, 298 K): δ 9.48 (s, −O*H*, 2H), 9.16 (d, *J* = 6.9 Hz, bipy-*H*, 4H), 8.93 (d, *J* = 6.8 Hz, bipy-*H*, 4H), 8.59 (d. *J* = 6.9 Hz, bipy-*H*, 4H), 8.53 (d, *J* = 6.8 Hz, bipy-*H*, 4H), 7.71 (s, Ar*H*, 4H), 7.26 (d, *J* = 7.5 Hz, Ar*H*, 4H), 6.95 (t, *J* = 7.5 Hz, Ar*H*, 2H), 4.44 (s, −ArC*H*_2_Ar-, 6H), 4.43 and 3.71 (AX system, *J* = 13.3 Hz, −ArC*H*_2_Ar–, 8H) 4.10 (t, *J* = 5.9 Hz, −OC*H*_2_CH_2_CH_3_,4H), 2.12 (m, −OCH_2_C*H*_2_CH_3_, 4H), 1.39 (t, *J* = 7.4 Hz −OCH_2_CH_2_C*H*_3_, 6H). ^13^C{^1^H} NMR (CD_3_OD, 100 MHz, 298 K): δ 151.0, 148.0, 146.7, 133.6, 131.7, 130.9, 130.3, 128.4, 128.3, 128.2, 128.0, 126.8, 125.3, 124.5, 120.4, 79.9, 49.6, 31.8, 24.6, 11.5. HRMS: *m*/*z* [M – H]^+^ calcd for C_56_H_56_N_4_O_4_^+^ 848.4296; found 848.4318.

### Synthesis of Derivative **V6**(H)_2_^4+^·(Cl^–^)_2_·(I^–^)_2_

Derivative **3** (50 mg, 0.053 mmol) and Zincke’s salt **6** (159 mg, 0.317 mmol) were dissolved in a mixture of ethanol/tetrahydrofuran/methanol (4:2:1 35 mL). The reaction was stirred for 24 h under reflux (70 °C). Then the solvent was removed by a rotary evaporator, and the crude product was triturated with water, filtered, and dried under vacuum to give **V6**(H)_2_^4+^·(Cl^–^)_2_·(I^–^)_2_ in 46% yield (38 mg, 0.024 mmol) as a red amorphous solid. ^1^H NMR (CD_3_OD, 600 MHz, 298 K): δ 9.23 (s, −OH, 2H), 8.99 (overlapped, bipy-H, 8H), 8.75 (overlapped, bipy-H, 8H), 8.17 (d, ArH, 12.0 Hz, 2H), 7.13–6.92 (overlapped, ArH, 12H), 4.55 (s, −NCH_3_, 6H), 4.19–3.86 (overlapped, ArCH_2_Ar, 12H), 3.53 (s, −OCH_3_, 12H), 1.26 (s, C–(CH_3_)_3_ 36H). ^13^C{^1^H} NMR ((CD_3_)_2_SO, 150 MHz, 298 K): δ 155.1, 150.1, 146.6, 135.4, 132.4, 129.6, 129.0, 127.3, 126.7, 126.4, 125.9, 123.7, 120.0, 70.0, 59.9, 34.1, 31.5, 29.1. HRMS: *m*/*z* [M – H]^+^ calcd for C_84_H_95_N_4_O_6_^+^ 1255.7235; found 1255.7243; [M + I]^+^ calcd for C_84_H_96_IN_4_O_6_^+^ 1383.6375; found 1383.6359.

### Synthesis of Derivative **V6**(H)_2_^4+^·(PF_6_^–^)_4_

**V6**(H)_2_^4+^·(Cl^–^)_2_·(I^–^)_2_ (30 mg, 0.019 mmol) was dissolved in 30 mL of acetonitrile, and NaPF_6_ (13 mg, 0.076 mmol) was added. The mixture was stirred for 15 h at room temperature. The formation of a precipitate was observed, and subsequently, it was filtered. The acetonitrile solution was evaporated, and 29 mg (0.016 mmol) of derivative **V6**(H)_2_^4+^·(PF_6_^–^)_4_ was obtained with a yield of 84% as an orange amorphous solid. ^1^H NMR (CD_3_CN, 600 MHz, 298 K): δ 9.49 (s, −OH, 2H), 8.88 (overlapped, bipy-H, 8H), 8,45 (overlapped, bipy-H, 8H), 7.24–7.13 (overlapped, ArH, 12H), 4.43 (s, −NCH_3_, 6H), 4.06–4.01 (overlapped, ArCH_2_Ar, 12H), 3.64 (s, −OCH_3_, 12H), 1.16(s, C–(CH_3_)_3_ 36H). ^13^C{^1^H} NMR (CD_3_CN, 150 MHz, 298 K): δ 125.9, 149.7, 149.2, 147.6, 147.3, 146.6, 144.9, 134.2, 130.3, 130.2, 127.4, 127.0, 126.8, 126.4, 123.2, 61.5, 48.7, 34.0, 30.7, 29.4. HRMS (MALDI): *m*/*z* [M – H]^+^ calcd for C_84_H_95_N_4_O_6_^+^ 1255.7235; found 1255.7244; [M + PF_6_]^+^ calcd for C_84_H_96_PN_4_O_6_F_6_^+^ 1401.6955; found 1401.6916.

## Supporting Information Available

Details of 1D and 2D NMR spectra, HR mass spectra, UV–vis data, and DFT calculations; UV–vis titrations; and details of the calculation of association constants (PDF)

## References

[ref1] aBrooker L. G. S.; Keyes G. H.; Sprague R. H.; VanDyke R. H.; VanLare E.; VanZandt G.; White F. L. Studies in the Cyanine Dye Series. XI. 1 The Merocyanines. J. Am. Chem. Soc. 1951, 73, 5326–5332. 10.1021/ja01155a095.

[ref2] aBicker K. L.; Wiskur S. L.; Lavigne J. J. Colorimetric Sensor Design. In Chemosensors; Wang B.; Anslyn E. V., Eds.; John Wiley & Sons, Inc.: Hoboken, NJ, 2011; pp 275–295.

[ref3] aSun X.; Dahlhauser S. D.; Anslyn E. V. New Autoinductive Cascade for the Optical Sensing of Fluoride: Application in the Detection of Phosphoryl Fluoride Nerve Agents. J. Am. Chem. Soc. 2017, 139, 4635–4638. 10.1021/jacs.7b01008. 28291353

[ref4] aLötzsch D.; Eberhardt V.; Rabe C. Ullmann’s Encyclopedia of Industrial Chemistry; Wiley-VCH Verlag GmbH & Co. KGaA: Weinheim, Germany, 2016.

[ref5] aReichardt C. Solvatochromic Dyes as Solvent Polarity Indicators. Chem. Rev. 1994, 94, 2319–2358. 10.1021/cr00032a005.

[ref6] aCiardelli F.; Bertoldo M.; Bronco S.; Pucci A.; Ruggeri G.; Signori F. The Unique Optical Behaviour of Bio-Related Materials with Organic Chromophores: Optical Behaviour of Bio-Related Materials. Polym. Int. 2013, 62, 22–32. 10.1002/pi.4395.

[ref7] Machado V. G.; Stock R. I.; Reichardt C. Pyridinium-N-Phenolate Betaine Dyes. Chem. Rev. 2014, 114, 10429–10475. 10.1021/cr5001157. 25216276

[ref8] aBaeyer A.; Villiger V. Dibenzalaceton Und Triphenylmethan. Ein Beitrag Zur Farbtheorie. Ber. Dtsch. Chem. Ges. 1902, 35, 1189–1201. 10.1002/cber.190203501197.

[ref9] aGutsche C. D. Calixarenes, An Introduction, 2nd ed.; The Royal Society of Chemistry: Cambridge, 2008.

[ref10] Della Sala P.; Talotta C.; Capobianco A.; Soriente A.; De Rosa M.; Neri P.; Gaeta C. Synthesis, Optoelectronic, and Supramolecular Properties of a Calix[4]Arene–Cycloparaphenylene Hybrid Host. Org. Lett. 2018, 20, 7415–7418. 10.1021/acs.orglett.8b03134. 30431286

[ref11] Iuliano V.; Talotta C.; Gaeta C.; Soriente A.; De Rosa M.; Geremia S.; Hickey N.; Mennucci B.; Neri P. Negative Solvatochromism in a N -Linked p -Pyridiniumcalix[4]Arene Derivative. Org. Lett. 2019, 21, 2704–2707. 10.1021/acs.orglett.9b00683. 30938161

[ref12] aZincke Th.; Heuser G.; Möller W.; Ueber I. Dinitrophenylpyridiniumchlorid und dessen Umwandlungsproducte. Justus Liebigs Ann. Chem. 1904, 333, 296–345. 10.1002/jlac.19043330212.

[ref13] Casnati A.; Domiano L.; Pochini A.; Ungaro R.; Carramolino M.; Oriol Magrans J.; M Nieto P.; López-Prados J.; Prados P.; de Mendoza J.; G Janssen R.; Verboom W.; N Reinhoudt D. Synthesis of Calix[6]Arenes Partially Functionalized at the Upper Rim. Tetrahedron 1995, 51, 12699–12720. 10.1016/0040-4020(95)00826-T.

[ref14] Robertson L.; Hartley R. C. Synthesis of N-arylpyridinium salts bearing a nitrone spin trap as potential mitochondria-targeted antioxidants. Tetrahedron 2009, 65, 5284–5292. 10.1016/j.tet.2009.04.083. 19693262 PMC2722452

[ref15] Bifulco G.; Riccio R.; Gaeta C.; Neri P. Quantum Mechanical Calculations of Conformationally Relevant 1H and 13C NMR Chemical Shifts of N-, O-, and S-Substituted Calixarene Systems. Chem. – Eur. J. 2007, 13, 7185–7194. 10.1002/chem.200700238. 17566131

[ref16] Kurland R. J.; Rubin M. B.; Wise W. B. Inversion Barrier in Singly Bridged Biphenyls. J. Chem. Phys. 1964, 40, 2426–2427. 10.1063/1.1725541.

[ref17] Gaeta C.; Talotta C.; Neri P. Pseudorotaxane orientational stereoisomerism driven by π–electron density. Chem. Commun. 2014, 50, 9917–9920. 10.1039/C4CC04668D. 25033221

[ref18] Frisch M. J. Gaussian 16, revision A.02; Gaussian, Inc.: Wallingford, CT, 2016.

[ref19] aBakić M. T.; Iuliano V.; Talotta C.; Geremia S.; Hickey N.; Spinella A.; De Rosa M.; Soriente A.; Gaeta C.; Neri P. Threading of Conformationally Stable Calix[6]Arene Wheels Substituted at the Methylene Bridges. J. Org. Chem. 2019, 84, 11922–11927. 10.1021/acs.joc.9b01779. 31418261

[ref20] aGaeta C.; Martino M.; Neri P. Conformational Templation in a Singly Bridged Calix[7]Arene Derivative Induced by Alkali Metal Cations. Org. Lett. 2006, 8, 4409–4412. 10.1021/ol061606g. 16986912

[ref21] aLankshear M. D.; Beer P. D. Strategic Anion Templation. Coord. Chem. Rev. 2006, 250, 3142–3160. 10.1016/j.ccr.2006.04.018.

[ref22] Kahlfuss C.; Milet A.; Wytko J.; Weiss J.; Saint-Aman E.; Bucher C. Hydrogen-Bond Controlled π-Dimerization in Viologen-Appended Calixarenes: Revealing a Subtle Balance of Weak Interactions. Org. Lett. 2015, 17, 4058–4061. 10.1021/acs.orglett.5b01982. 26270244

[ref23] Constantin V.-A.; Cao L.; Sadaf S.; Walder L. Oligo-Viologen/SWCNT Nano-Composites: Preparation and Characterization: Oligo-Viologen/SWCNT Nano-Composites. Phys. Status Solidi B 2012, 249, 2395–2398. 10.1002/pssb.201200121.

[ref24] In agreement with literature data (see page 10437 of ref (7)), the synthesized derivatives showed no emission properties

[ref25] aMennucci B. Polarizable Continuum Model: Polarizable Continuum Model. WIREs Comput. Mol. Sci. 2012, 2, 386–404. 10.1002/wcms.1086.

[ref26] Struck O.; Chrisstoffels L. A. J.; Lugtenberg R. J. W.; Verboom W.; van Hummel G. J.; Harkema S.; Reinhoudt D. N. Head-to-Head Linked Double Calix[4]Arenes: Convenient Synthesis and Complexation Properties. J. Org. Chem. 1997, 62, 2487–2493. 10.1021/jo962138z. 11671587

[ref27] Fulmer G. R.; Miller A. J. M.; Sherden N. H.; Gottlieb H. E.; Nudelman A.; Stoltz B. M.; Bercaw J. E.; Goldberg K. I. NMR Chemical Shifts of Trace Impurities: Common Laboratory Solvents, Organics, and Gases in Deuterated Solvents Relevant to the Organometallic Chemist. Organometallics 2010, 29, 2176–2179. 10.1021/om100106e.

