# Supplementary material for: Chromogenic Properties of p-Pyridinium- and p-Viologen-Calixarenes and Their Cation-Sensing Abilities
Source: J Org Chem. 2021 Sep 1;86(18):13001–10. doi: 10.1021/acs.joc.1c01687 (PMC8453632; doi:10.1021/acs.joc.1c01687)
Supplement: Supplementary file 1 — jo1c01687_si_001.pdf [file jo1c01687_si_001.pdf]

Supporting Information

Chromogenic Properties of *p*-Pyridinium and *p*-ViologenCalixarenes and Their Cation-Sensing Abilities

V. Iuliano, P. Della Sala, C. Talotta,* L. Liguori, G. Monaco, E. Tiberio, C. Gaeta and P. Neri*

Department of Chemistry and Biology "A. Zambelli", University of Salerno, Via Giovanni Paolo II 132, I-84084 Fisciano, Salerno, Italy.

Table of Contents

^{1}H NMR spectrum of derivative **3** S4

$^{13}C\{^{1}H\}$ NMR spectrum of derivative **3** S5

HR-MS spectrum of derivative **3** S6

^{1}H NMR spectrum of derivative **P6**$(H)_2^{2+}\cdot(Cl^-)_2$ S7

$^{13}C\{^{1}H\}$ NMR spectrum of derivative **P6**$(H)_2^{2+}\cdot(Cl^-)_2$ S8

HR-MS spectrum of derivative **P6**$(H)_2^{2+}\cdot(Cl^-)_2$ S9

2D COSY spectrum of derivative **P6**$(H)_2^{2+}\cdot(Cl^-)_2$ S10

2D HSQC spectrum of derivative **P6**$(H)_2^{2+}\cdot(Cl^-)_2$ S11

^{1}H NMR spectrum of derivative **P6**$(H)_2^{2+}\cdot(BArF^-)_2$ S12

$^{13}C\{^{1}H\}$ NMR spectrum of derivative **P6**$(H)_2^{2+}\cdot(BArF^-)_2$ S13

HR-MS spectrum of derivative **P6**$(H)_2^{2+}\cdot(BArF^-)_2$ S14

2D HSQC spectrum of derivative **P6**$(H)_2^{2+}\cdot(BArF^-)_2$ S15

HT NMR spectra of derivative **P6**$(H)_2^{2+}\cdot(Cl^-)_2$ S16

LT NMR spectra of derivative **P6**$(H)_2^{2+}\cdot(BArF^-)_2$ S17

^{1}H NMR spectrum of derivative **V4**$(H)_2^{4+}\cdot(Cl^-)_2\cdot(I^-)_2$ S19

$^{13}C\{^{1}H\}$ NMR spectrum of derivative **V4**$(H)_2^{4+}\cdot(Cl^-)_2\cdot(I^-)_2$ S20

HR-MS spectrum of derivative **V4**$(H)_2^{4+}\cdot(Cl^-)_2\cdot(I^-)_2$ S21

2D COSY spectrum of derivative **V4**$(H)_2^{4+}\cdot(Cl^-)_2\cdot(I^-)_2$ S22

2D HSQC spectrum of derivative **V4**$(H)_2^{4+}\cdot(Cl^-)_2\cdot(I^-)_2$ S23

^{1}H NMR spectrum of derivative **V6**$(H)_2^{4+}\cdot(Cl^-)_2\cdot(I^-)_2$ S24

$^{13}C\{^{1}H\}$ NMR spectrum of derivative **V6**$(H)_2^{4+}\cdot(Cl^-)_2\cdot(I^-)_2$ S25

HR-MS spectrum of derivative **V6**$(H)_2^{4+}\cdot(Cl^-)_2\cdot(I^-)_2$ S26

^{1}H NMR spectrum of derivative **V6**$(H)_2^{4+}\cdot(PF_6^-)_4$ S27

$^{13}C\{^{1}H\}$ NMR spectrum of derivative **V6**$(H)_2^{4+}\cdot(PF_6^-)_4$ S28

HR-MS spectrum of derivative **V6**$(H)_2^{4+}\cdot(PF_6^-)_4$ S29

2D HSQC spectrum of derivative **V6**$(H)_2^{4+}\cdot(PF_6^-)_4$ S30

UV-Vis characterization S31

Acid-base UV-VIS titrations S34

Cation Sensing of derivative **V6**$(H)_1^{3+}$ S35

Computational details S35

^{1}H NMR of derivative **P6**$(H)_2^{2+}(Cl^-)_2$ in different solvents S54

^{1}H NMR of derivative **P6**$(H)_2^{2+}(Cl^-)_2$ and $P6(H)_1^+$ in acetonitrile S55

2D COSY of derivative **P6**$(H)_2^{2+}(Cl^-)_2$ in acetonitrile S56

2D COSY of derivative **P6**$(H)_1^+$ in acetonitrile S57

^{1}H NMR spectra of mixtures **P6**$(H)_1^+$/LiI and **P6**$(H)_1^+$/NaI S58

Determination of association constants for the formation of (Li@**P6**$(H)_1)^{2+}$ $\cdot(Cl^-)\cdot(I^-)$ and (Na@**P6**$(H)_1)^{2+}$ $\cdot(Cl^-)\cdot(I^-)$ complexes by UV/Vis titrations using Benesi-Hildebrand equation....................S59

References S61

^{1}H NMR spectrum of derivative 3

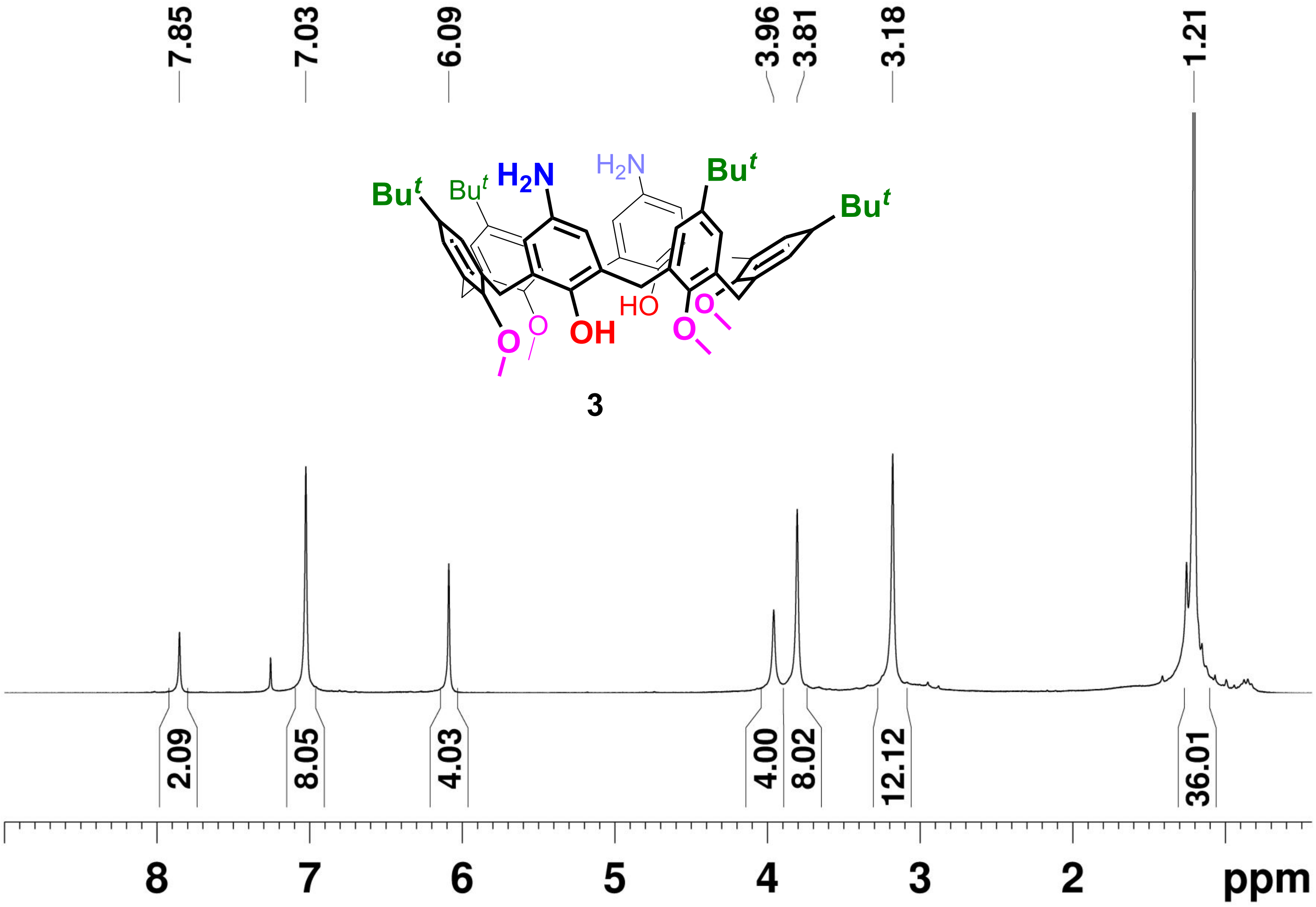

Figure S1. ^{1}H NMR spectrum of derivative **3** (300 MHz, $CDCl_3$, 298 K).

$^{13}C\{^1H\}$ NMR spectrum of derivative 3

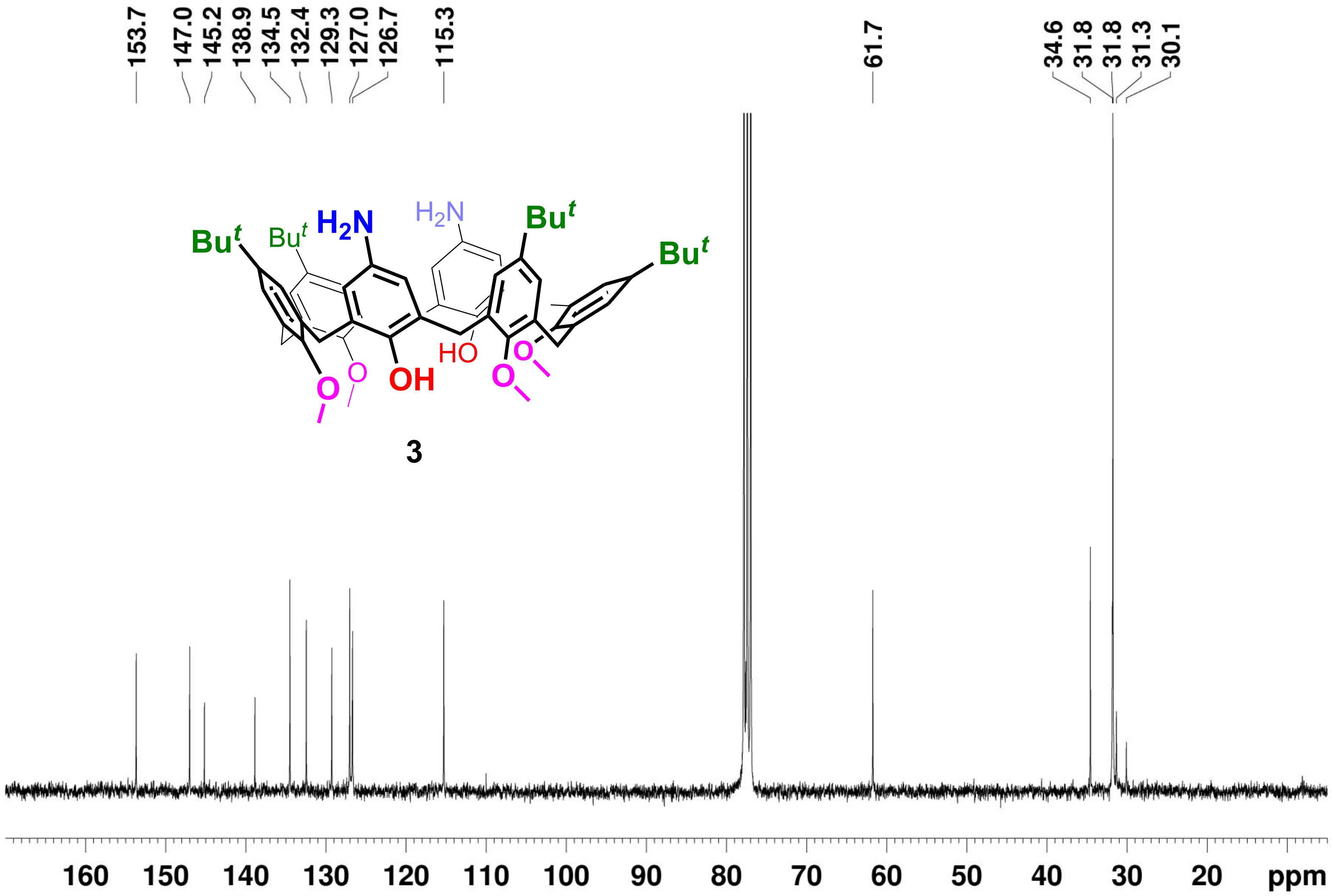

Figure S2. $^{13}C\{^1H\}$ NMR spectrum of derivative **3** (100 MHz, $CDCl_3$, 298 K).

HR-MS spectrum of derivative 3

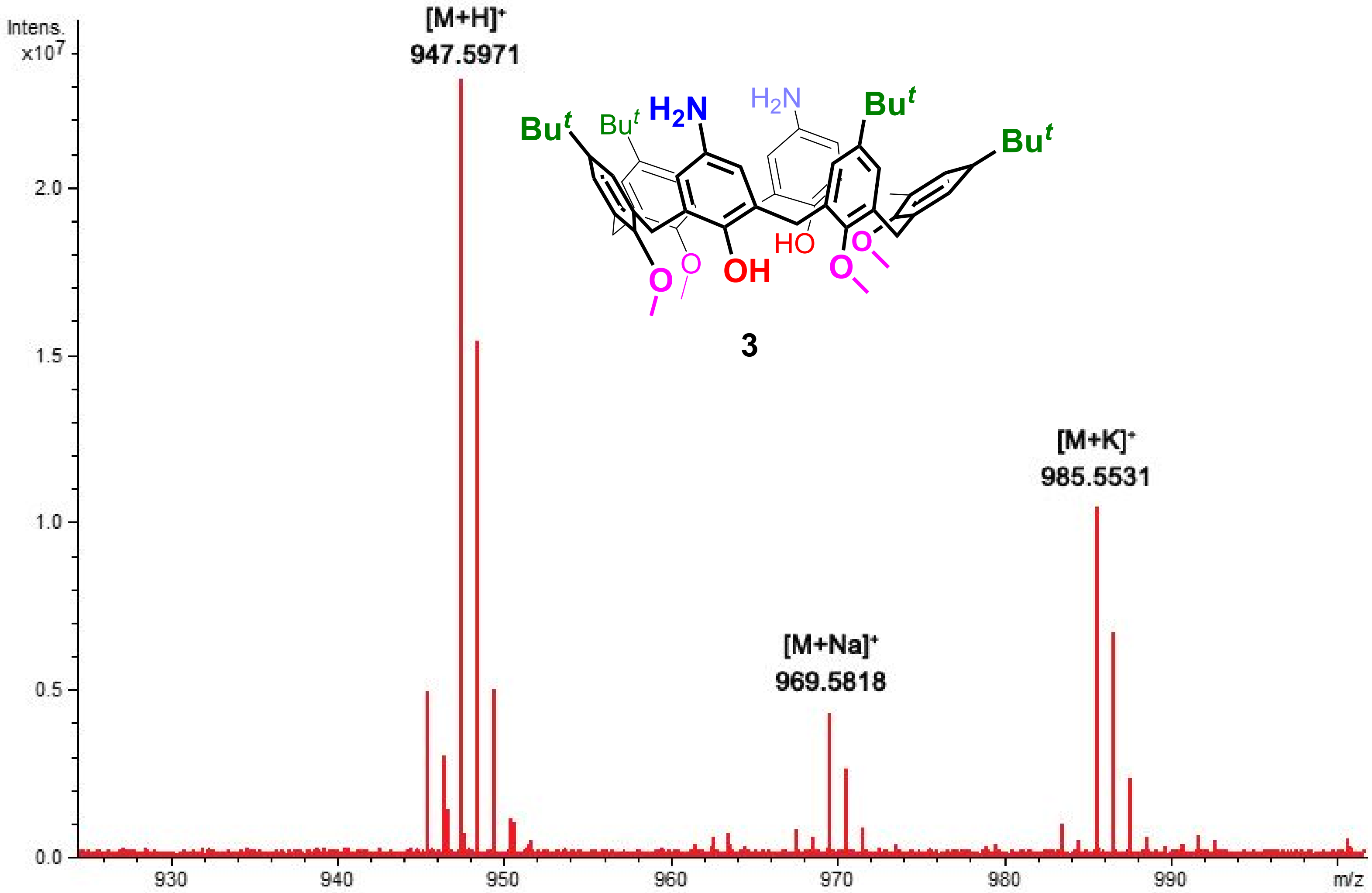

Figure S3. HR-MS spectrum of derivative **3**.

^1H NMR spectrum of derivative $P6(H)_2^{2+}\cdot(Cl^-)_2$

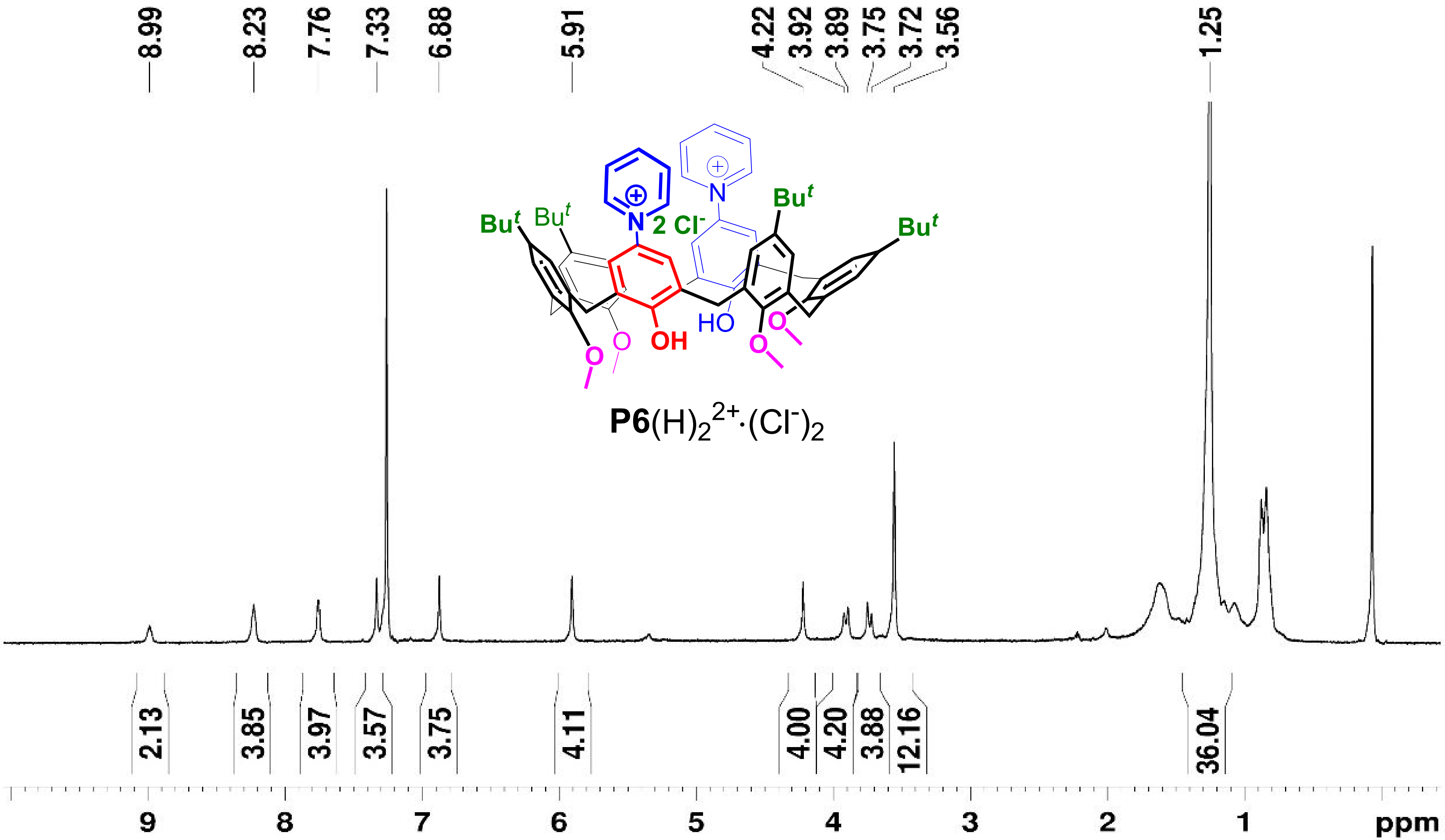

Figure S4. ^1H NMR spectrum of derivative **P6**$(H)_2^{2+}\cdot(Cl^-)_2$ (400 MHz, $CDCl_3$, 298 K).

$^{13}C\{^1H\}$ NMR spectrum of derivative $P6(H)_2^{2+}\cdot(Cl^-)_2$

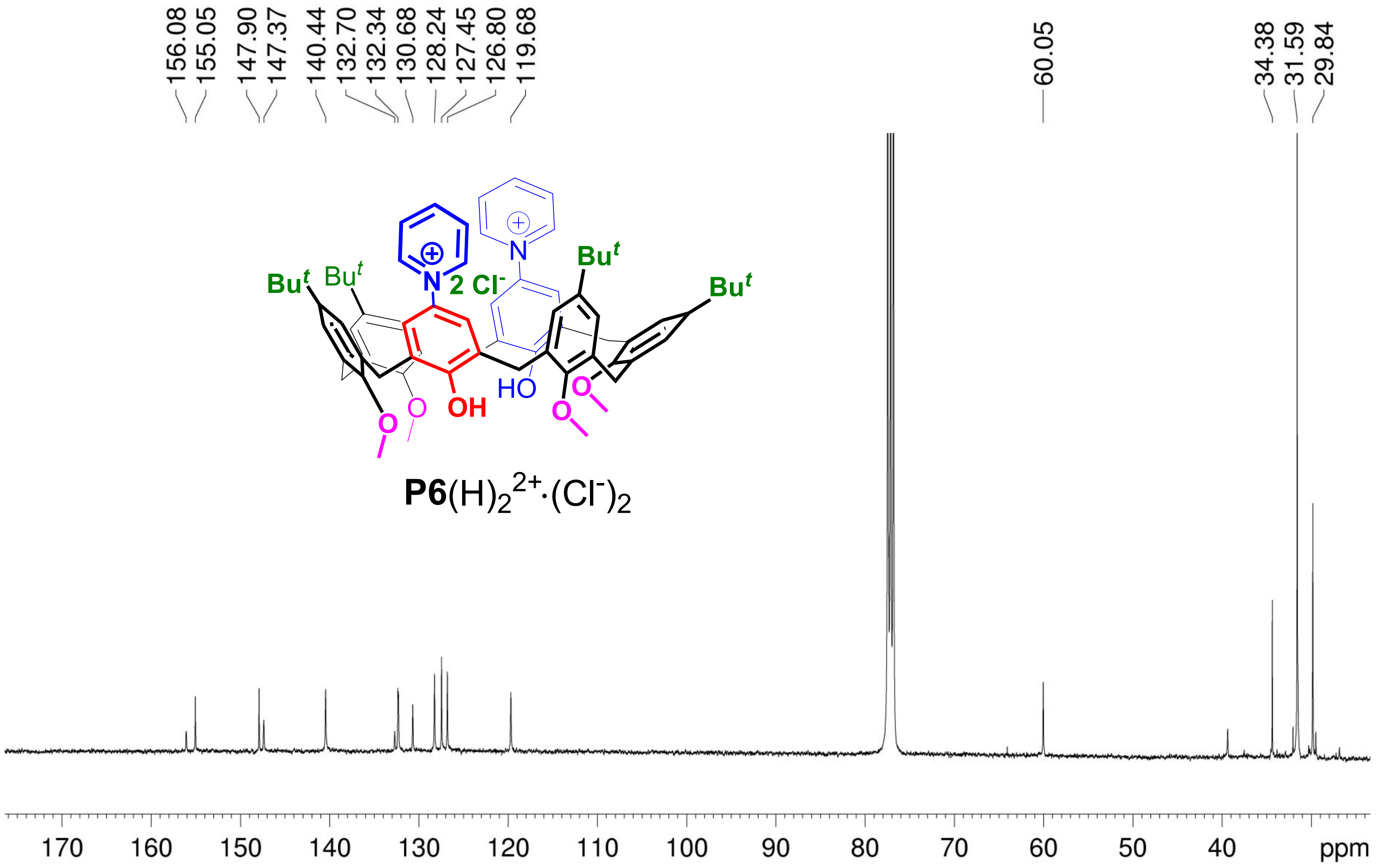

Figure S5. $^{13}C\{^1H\}$ NMR spectrum of derivative **P6**$(H)_2^{2+}\cdot(Cl^-)_2$ (100 MHz, $CDCl_3$, 298 K).

HR-MS spectrum of derivative P6$(H)_2^{2+}\cdot(Cl^-)_2$

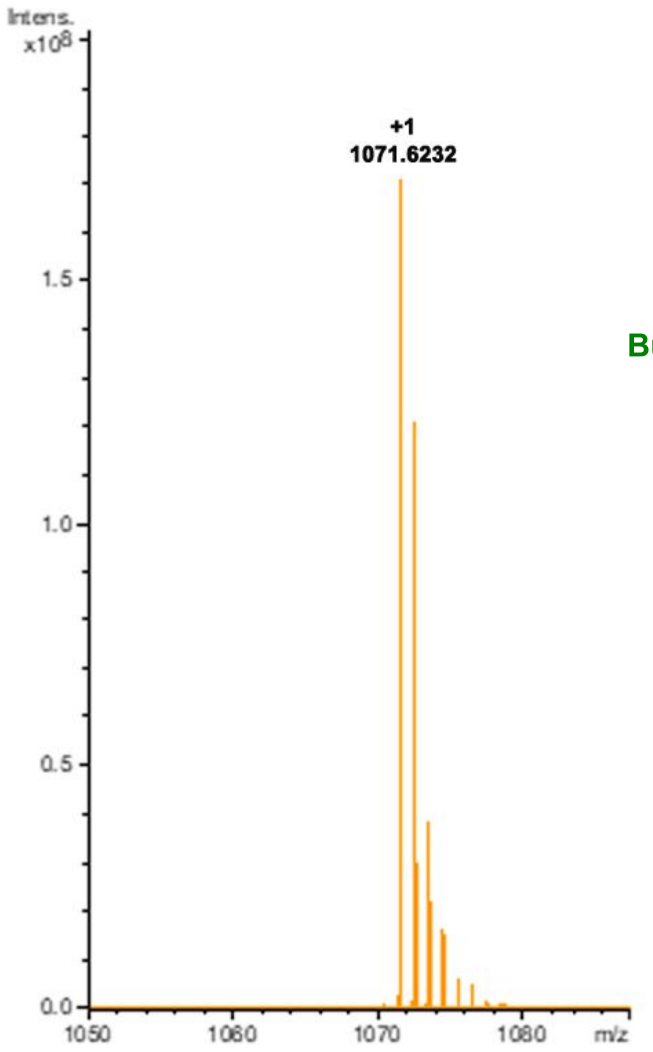

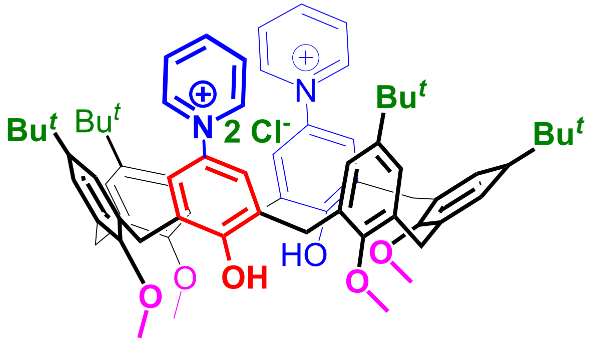

P6$(H)_2^{2+}\cdot(Cl^-)_2$

Figure S6. HR-MS spectrum of derivative **P6**$(H)_2^{2+}\cdot(Cl^-)_2$.

2D COSY spectrum of derivative P6(H)$_2^{2+}$·(Cl$^-$)$_2$

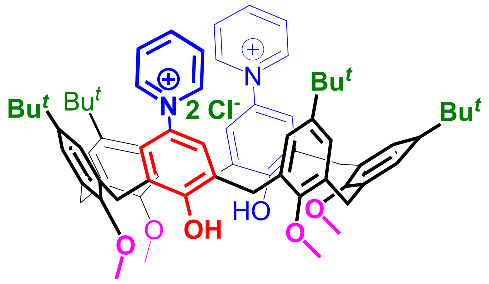

P6(H)$_2^{2+}$·(Cl$^-$)$_2$

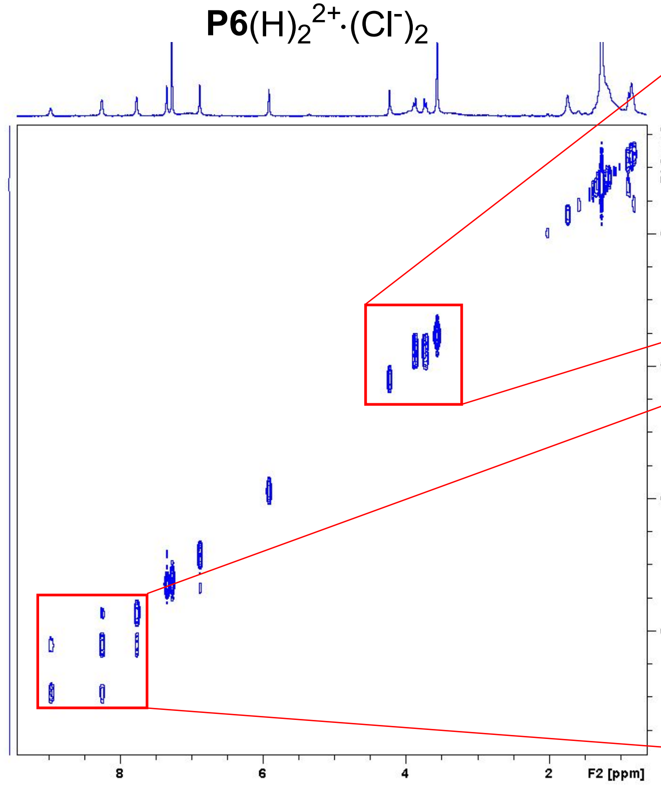

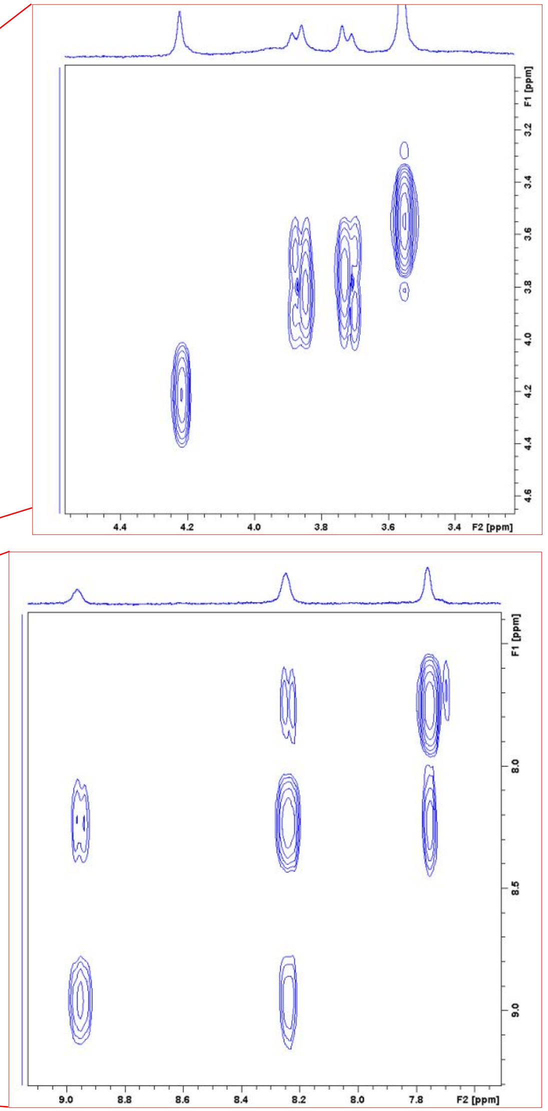

Figure S7. 2D COSY spectrum of derivative **P6**(H)$_2^{2+}$·(Cl$^-$)$_2$ (600 MHz, $CDCl_3$, 298 K).

2D HSQC spectrum of derivative P6(H)$_2^{2+}$·(Cl$^-$)$_2$

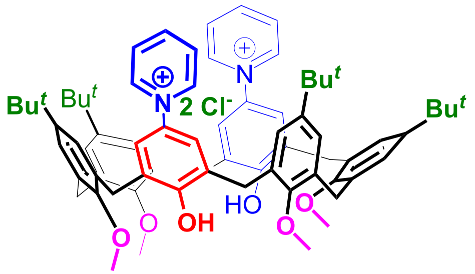

P6(H)$_2^{2+}$·(Cl$^-$)$_2$

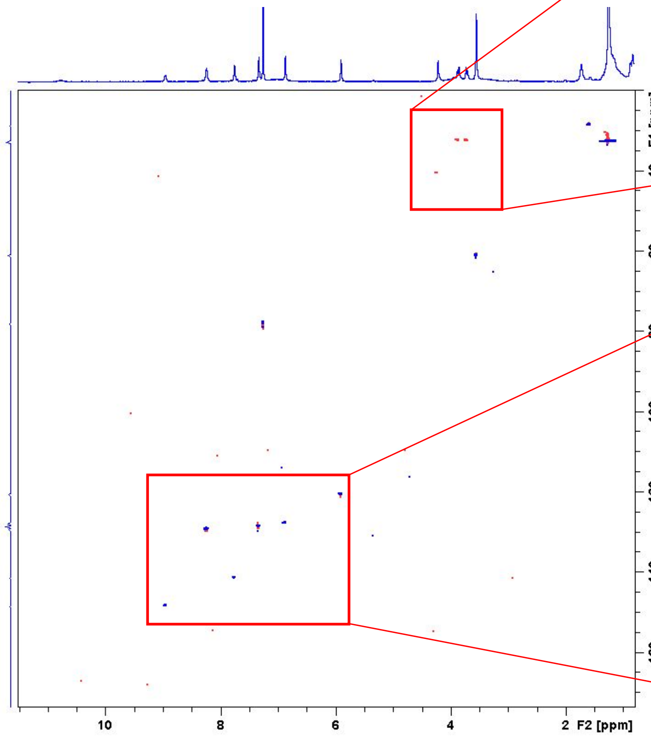
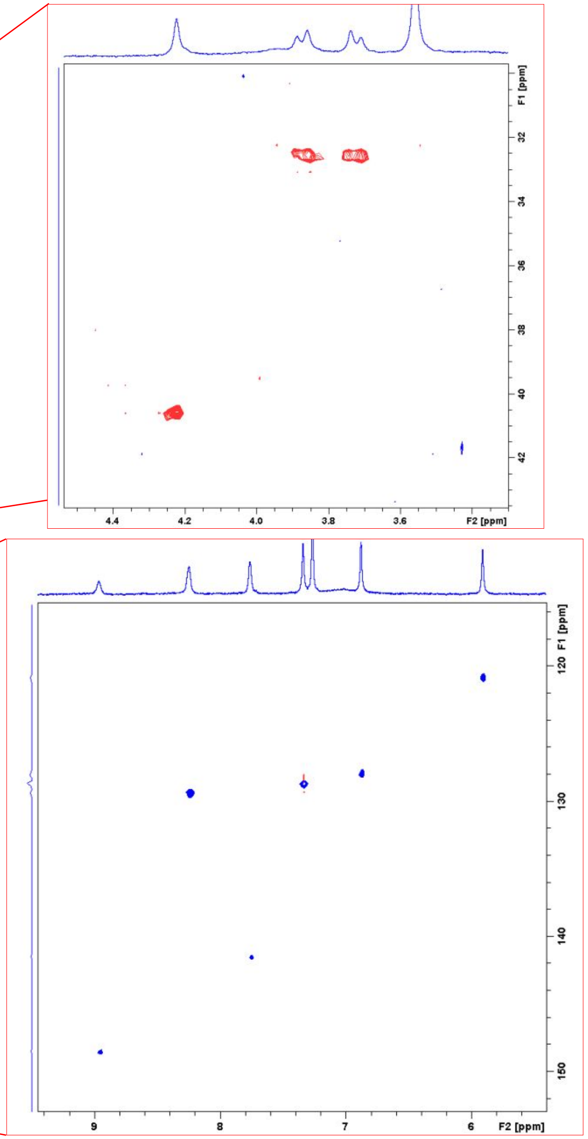

Figure S8. 2D HSQC spectrum of derivative **P6**(H)$_2^{2+}$·(Cl$^-$)$_2$ (600 MHz, $CDCl_3$, 298 K).

^{1}H NMR spectrum of derivative P6(H)$_2^{2+}$·(BArF$^-$)$_2$

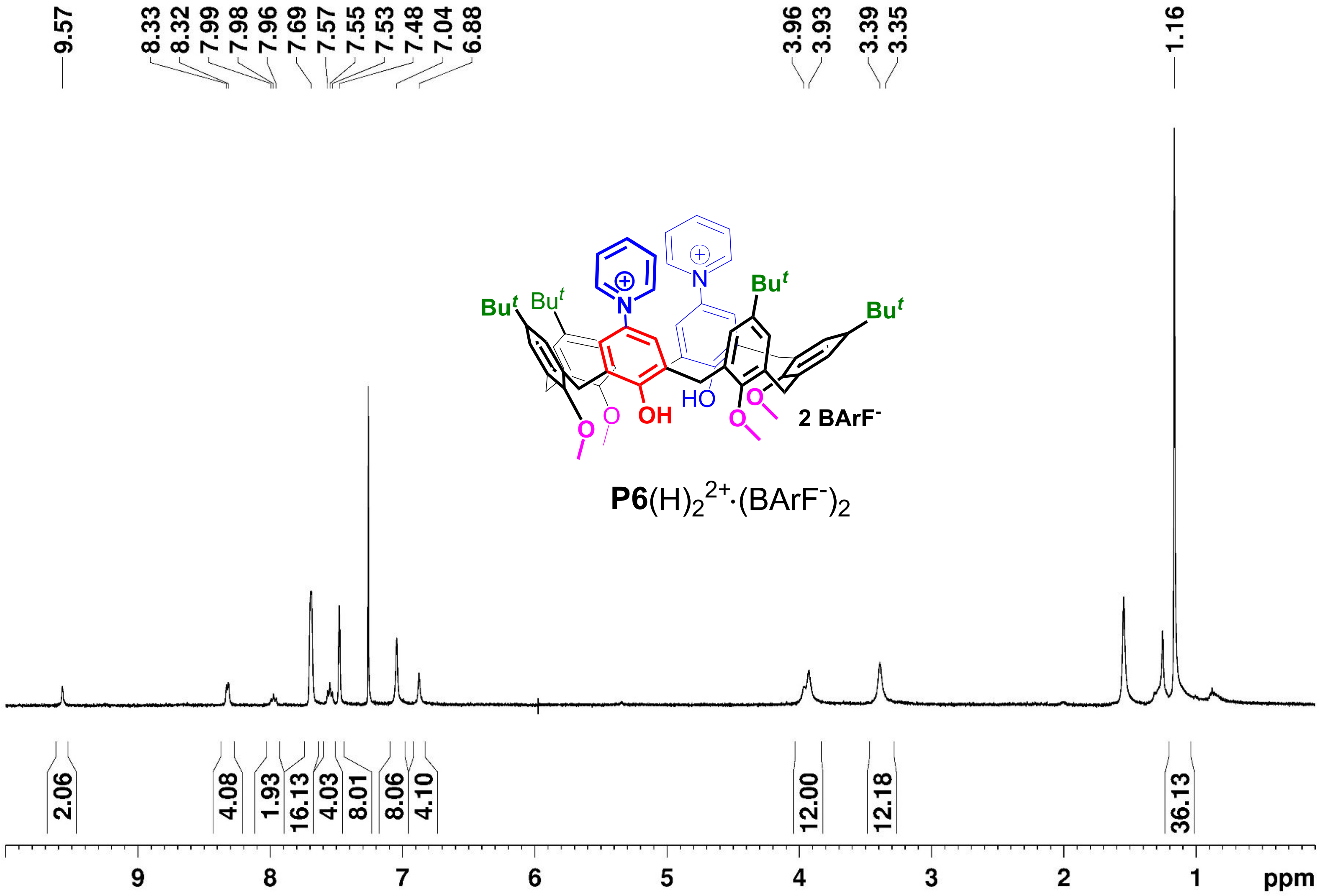

Figure S9. ^{1}H NMR spectrum of derivative **P6**(H)$_2^{2+}$·(BArF$^-$)$_2$ (400 MHz, $CDCl_3$, 298 K).

$^{13}C\{^1H\}$ NMR spectrum of derivative $P6(H)_2^{2+}\cdot(BArF^-)_2$

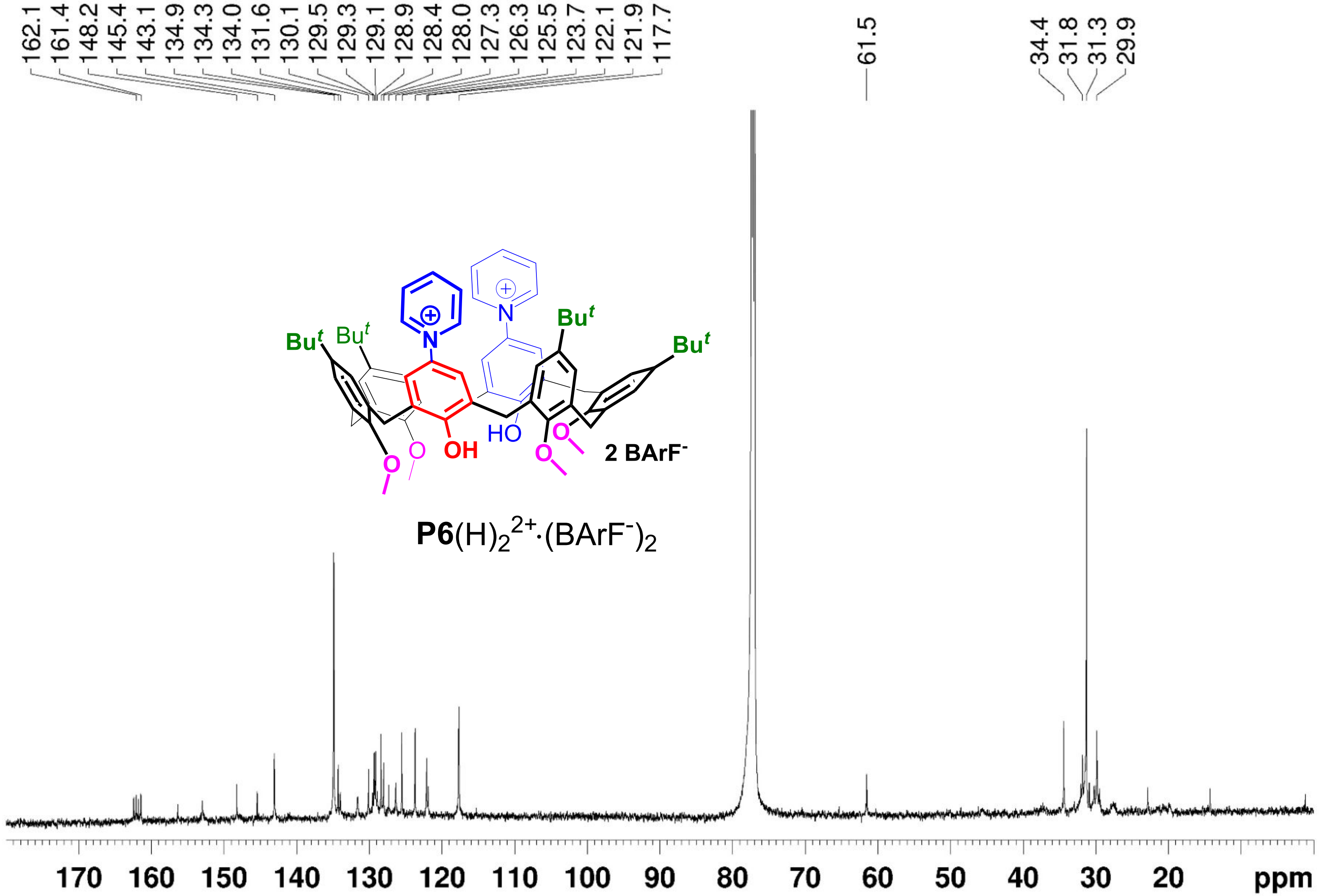

Figure S10. $^{13}C\{^1H\}$ NMR spectrum of derivative **P6**$(H)_2^{2+}\cdot(BArF^-)_2$ (100 MHz, $CDCl_3$, 298 K).

HR-MS spectrum of derivative P6(H)$_2^{2+}$·(BArF^{-})$_2$

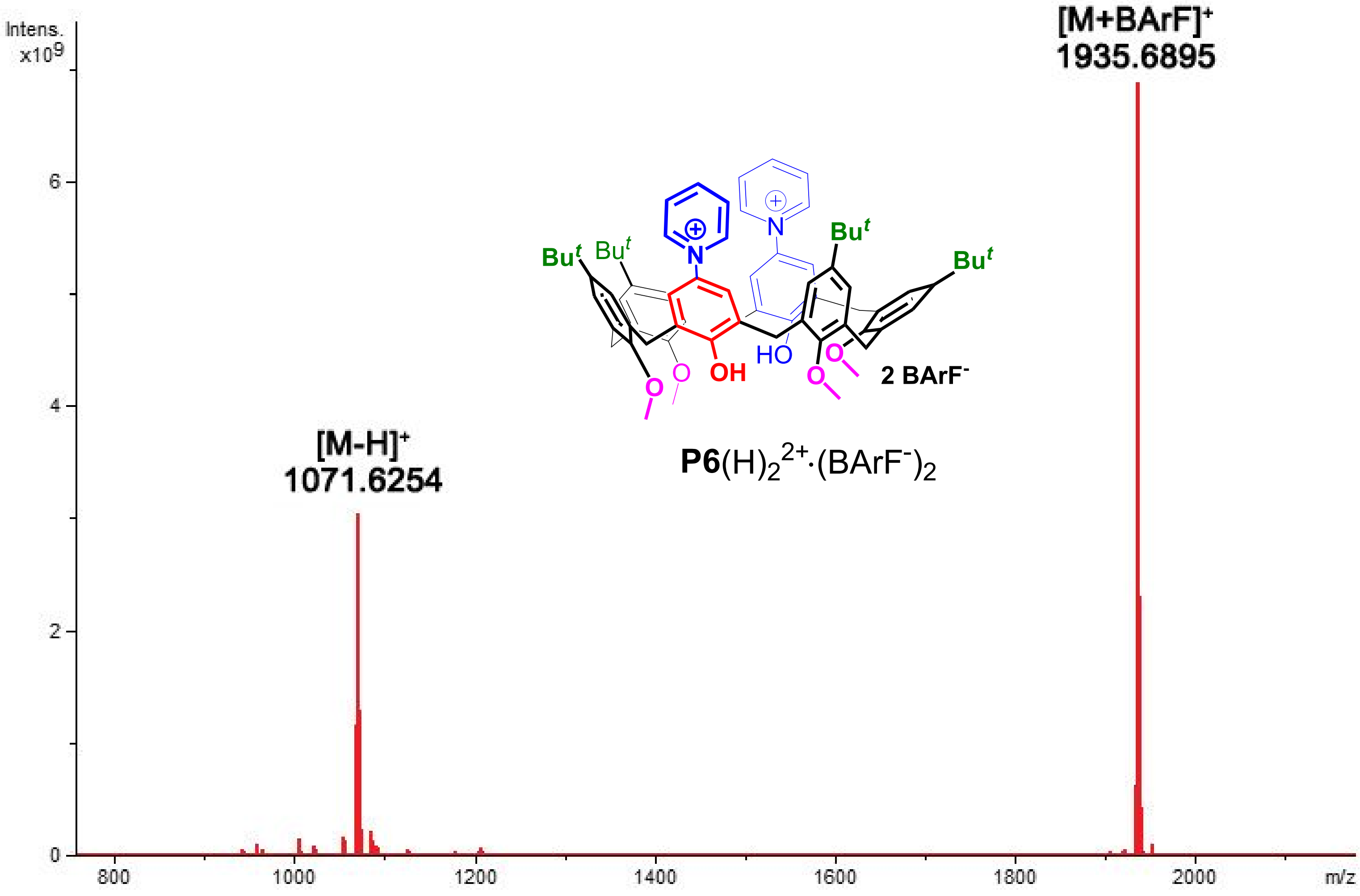

Figure S11. HR-MS spectrum of derivative **P6**(H)$_2^{2+}$·(BArF^{-})$_2$.

2D HSQC spectrum of derivative P6(H)$_2^{2+}$·(BArF$^-$)$_2$

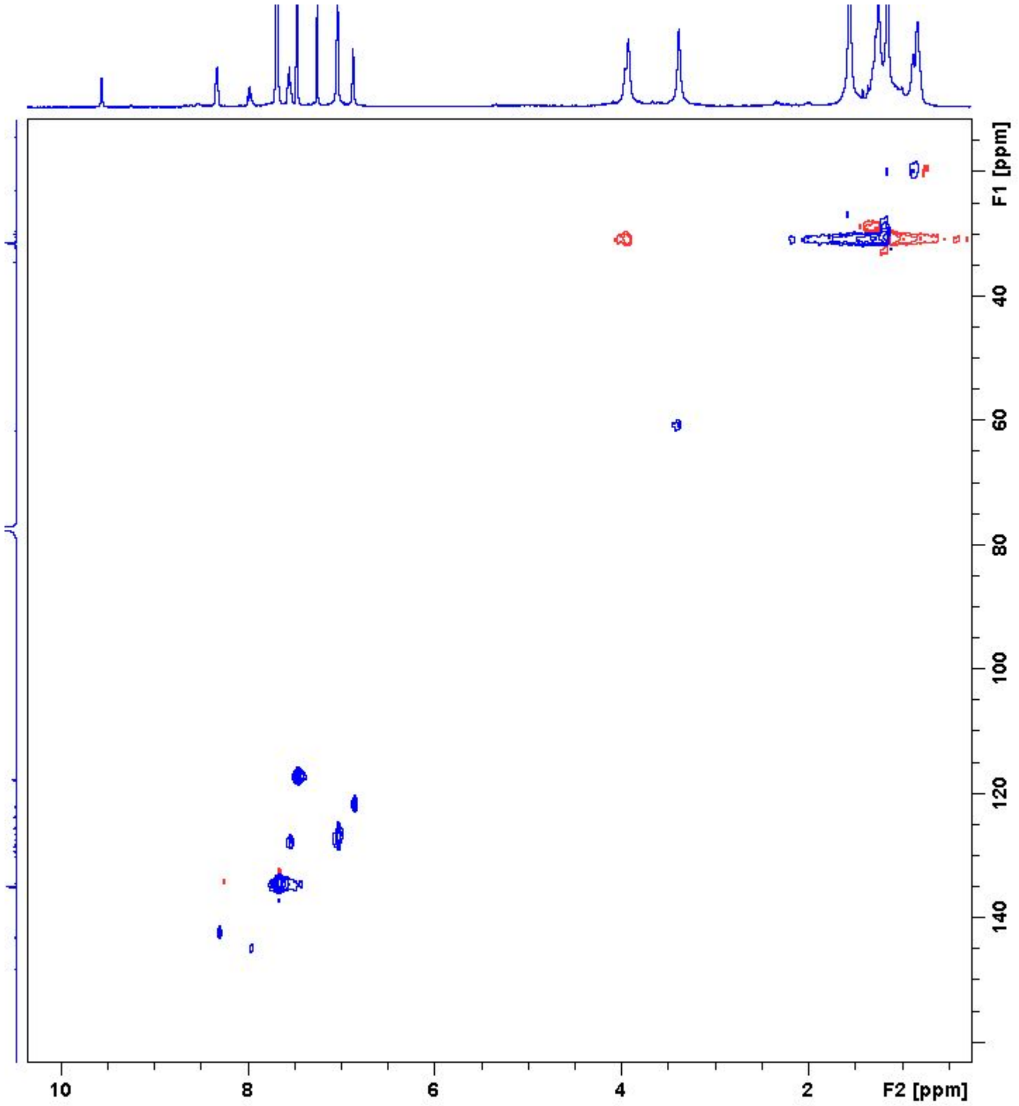

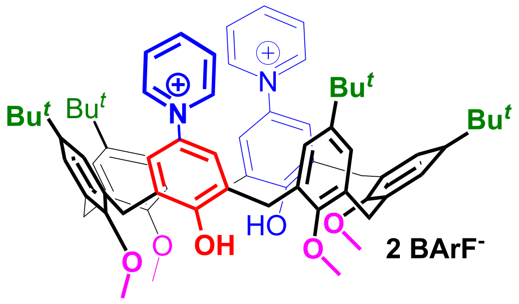

P6(H)$_2^{2+}$·(BArF$^-$)$_2$

Figure S12. 2D HSQC of derivative **P6**(H)$_2^{2+}$·(BArF$^-$)$_2$ (600 MHz, 298 K, $CDCl_3$)

HT NMR spectra of derivative P6(H)$_2^{2+}$·(Cl$^-$)$_2$

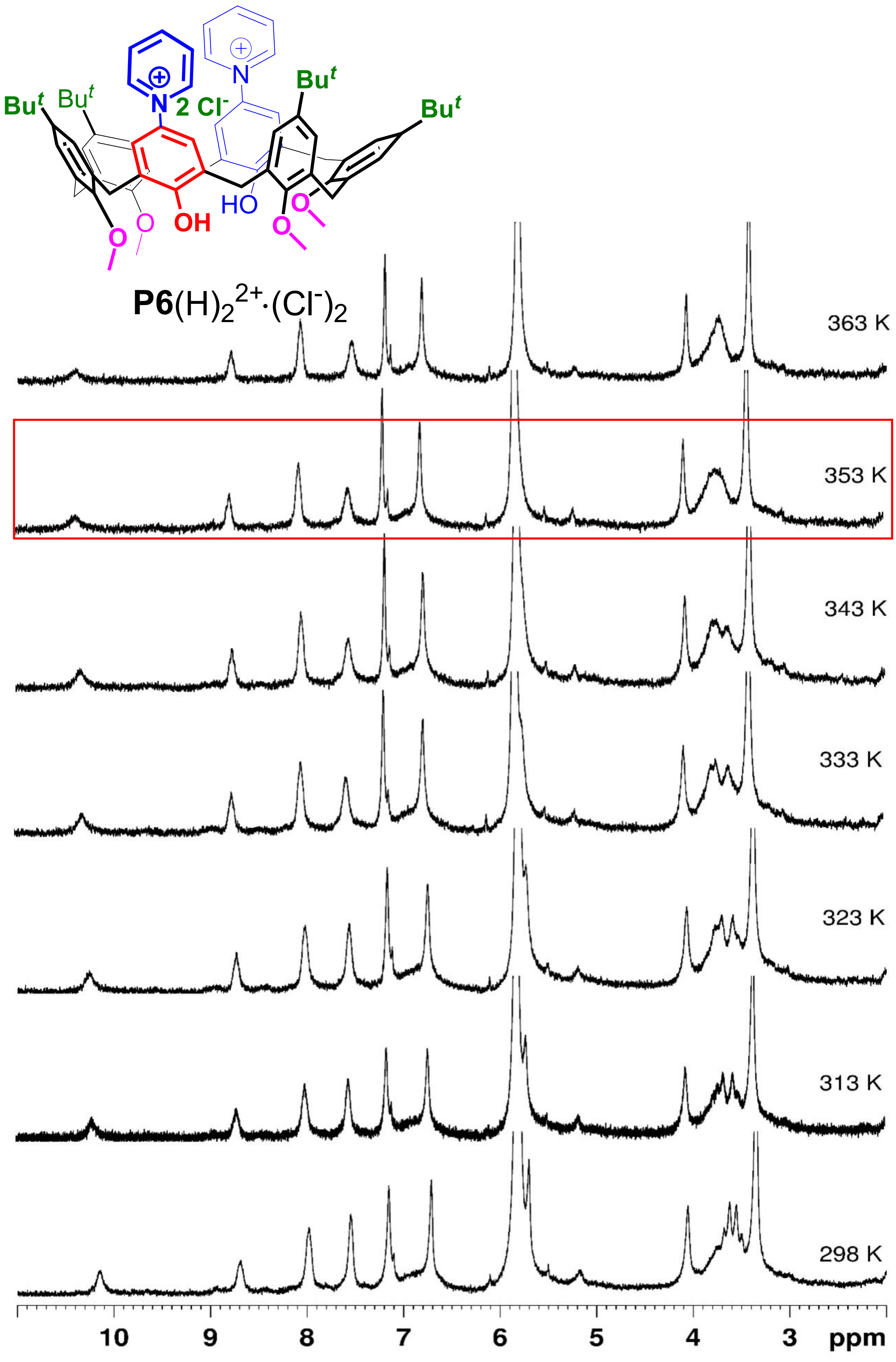

Figure S13. ^{1}H NMR spectra of derivative **P6**(H)$_2^{2+}$·(Cl$^-$)$_2$ (300 MHz, TCDE).

LT NMR spectra of derivative P6(H)$_2^{2+}$·(BArF$^-$)$_2$

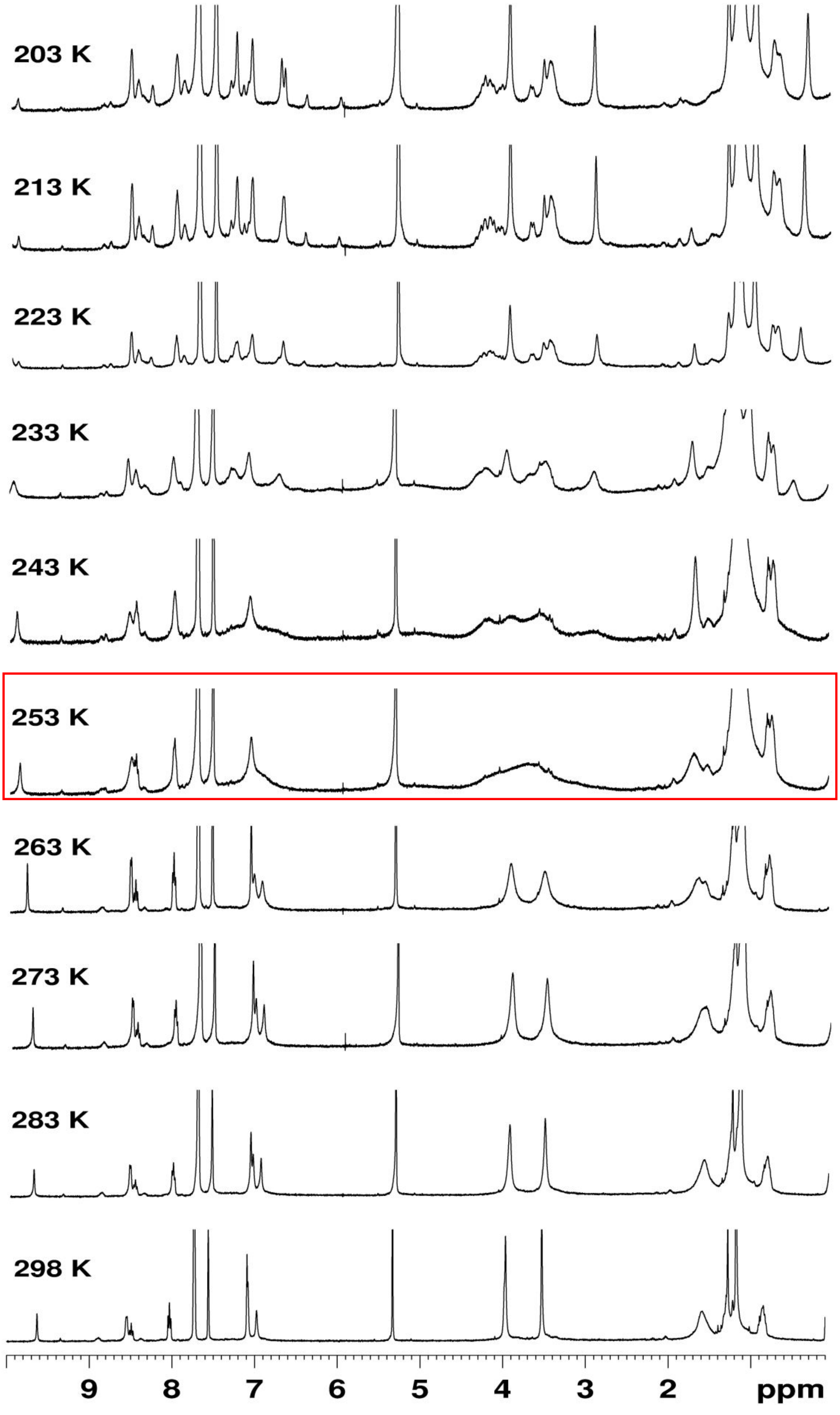

Figure S14. ^{1}H NMR spectra of derivative **P6**(H)$_2^{2+}$·(BArF$^-$)$_2$ (400 MHz, CD_2Cl_2).

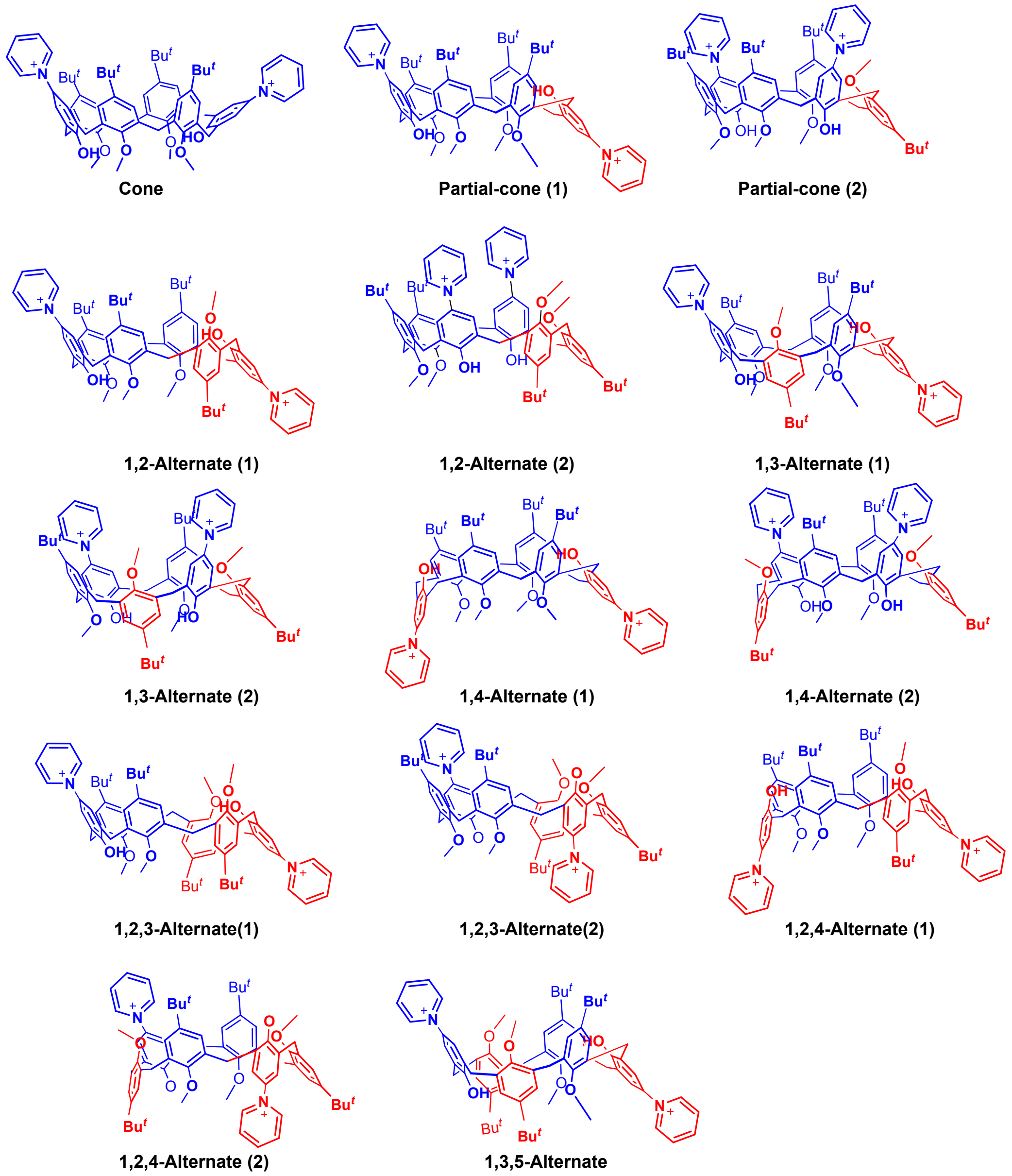

Figure S15. Possible discrete conformations of calix[6]arene **P6**$(H)_2^{2+}$

^{1}H NMR spectrum of derivative $V4(H)_2^{4+}\cdot(Cl^-)_2\cdot(I^-)_2$

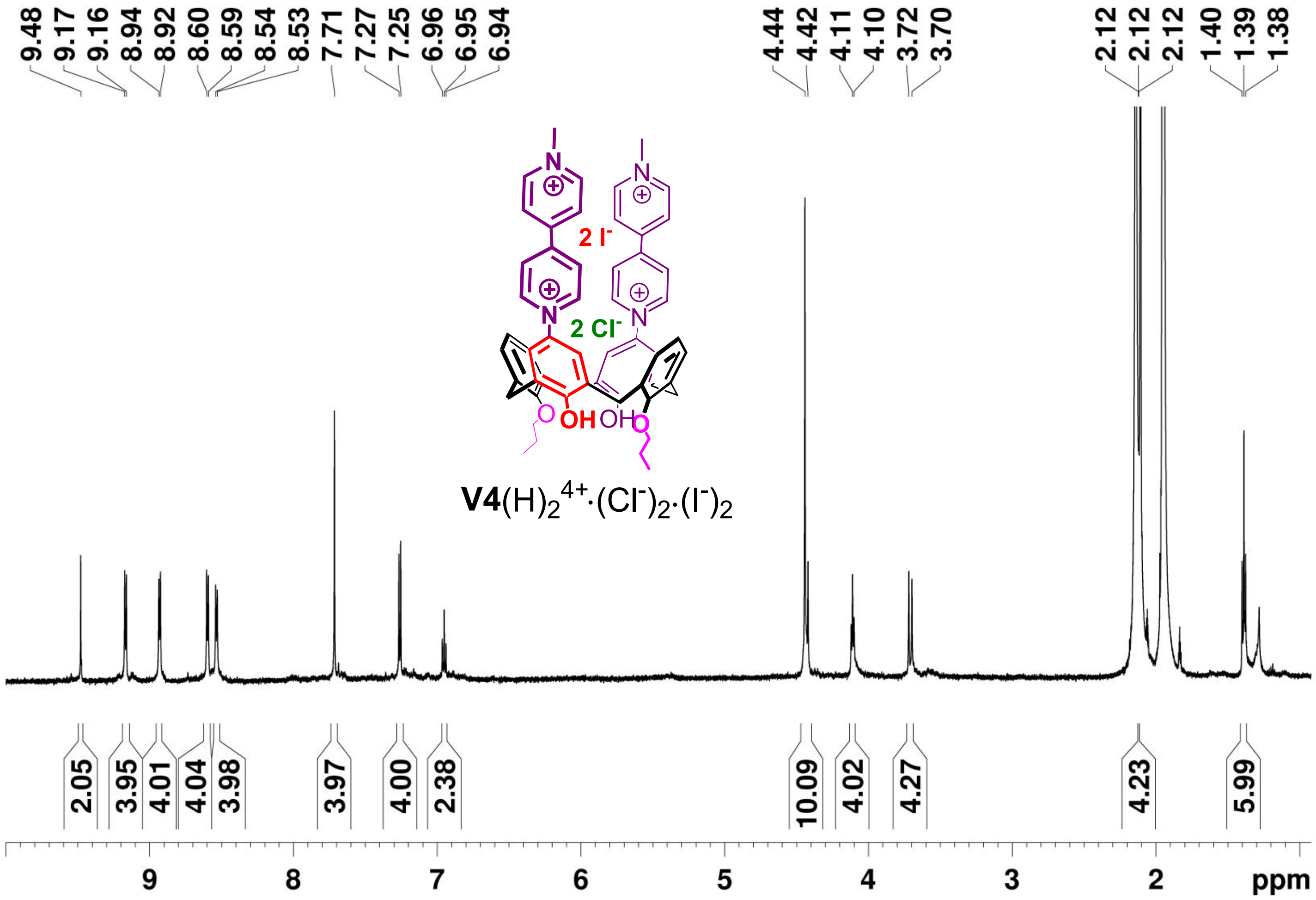

Figure S16. ^{1}H NMR spectrum of derivative **V4**$(H)_2^{4+}\cdot(Cl^-)_2\cdot(I^-)_2$ (600 MHz, CD_3CN, 298 K).

$^{13}C\{^1H\}$ NMR spectrum of derivative V4$(H)_2^{4+}\cdot(Cl^-)_2\cdot(I^-)_2$

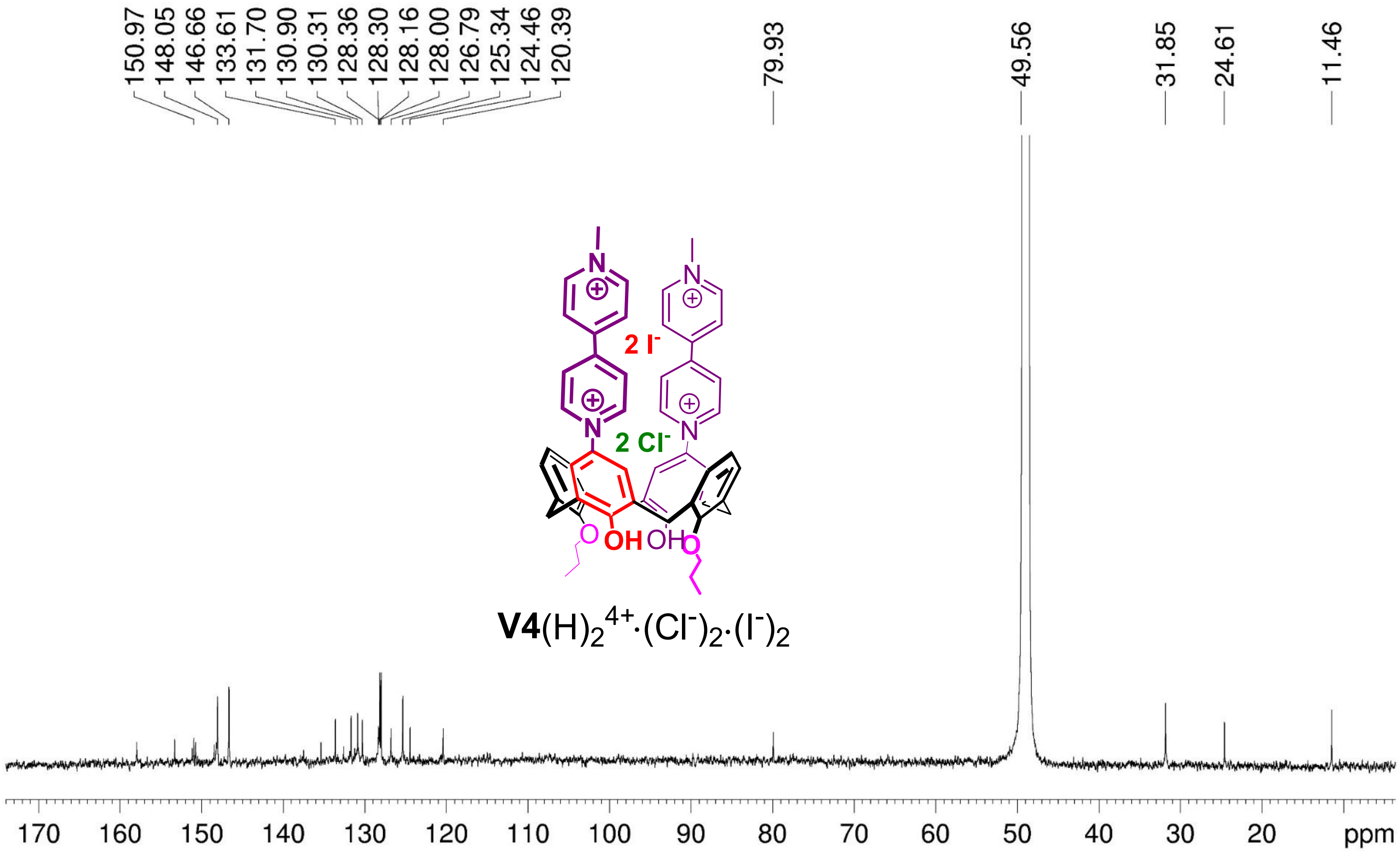

Figure S17. $^{13}C\{^1H\}$ NMR spectrum of derivative **V4**$(H)_2^{4+}\cdot(Cl^-)_2\cdot(I^-)_2$ (150 MHz, CD_3OD, 298 K).

HR-MS spectrum of derivative V4(H)$_2^{4+}$·(Cl$^-$)$_2$·(I$^-$)$_2$

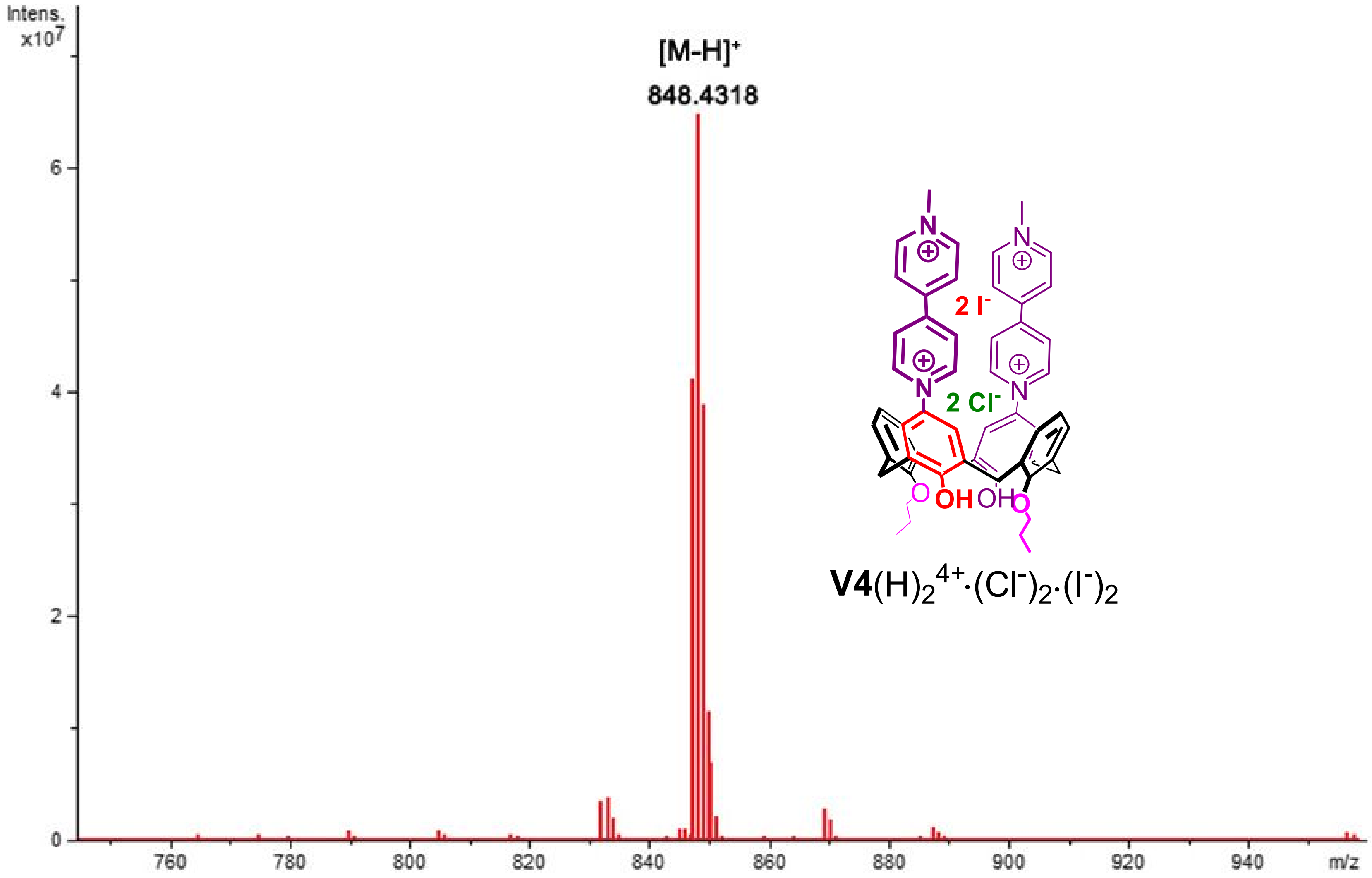

Figure S18. HR-MS spectrum of derivative **V4**(H)$_2^{4+}$·(Cl$^-$)$_2$·(I$^-$)$_2$.

2D COSY spectrum of derivative $V4(H)_2^{4+}\cdot(Cl^-)_2\cdot(I^-)_2$

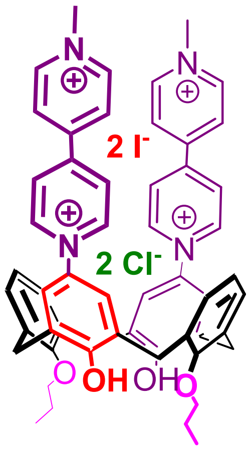

V4$(H)_2^{4+}\cdot(Cl^-)_2\cdot(I^-)_2$

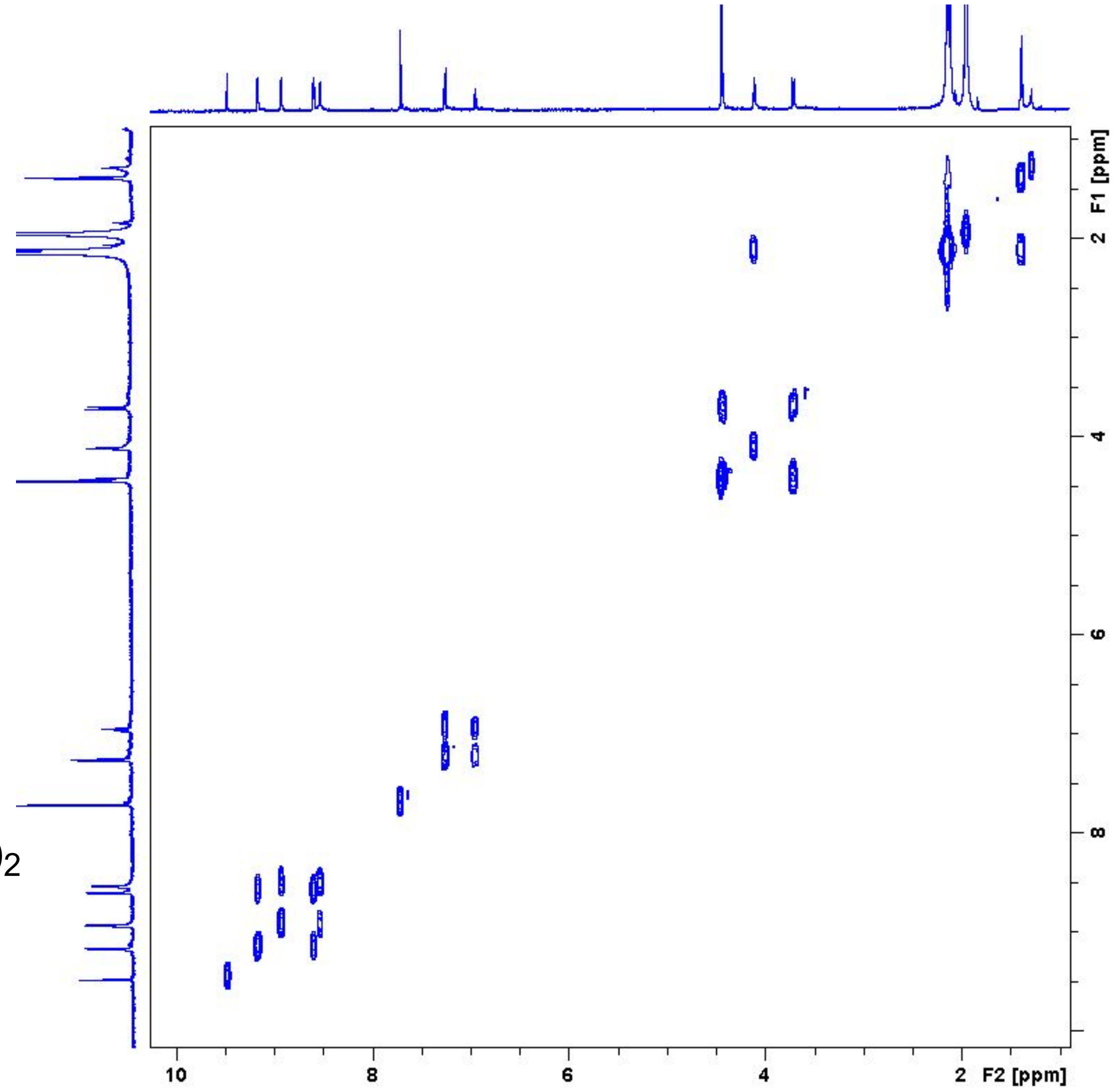

Figure S19. 2D COSY spectrum of derivative **V4**$(H)_2^{4+}\cdot(Cl^-)_2\cdot(I^-)_2$ (600 MHz, CD_3CN, 298 K).

2D HSQC spectrum of derivative $V4(H)_2^{4+}{\cdot}(Cl^-)_2{\cdot}(I^-)_2$

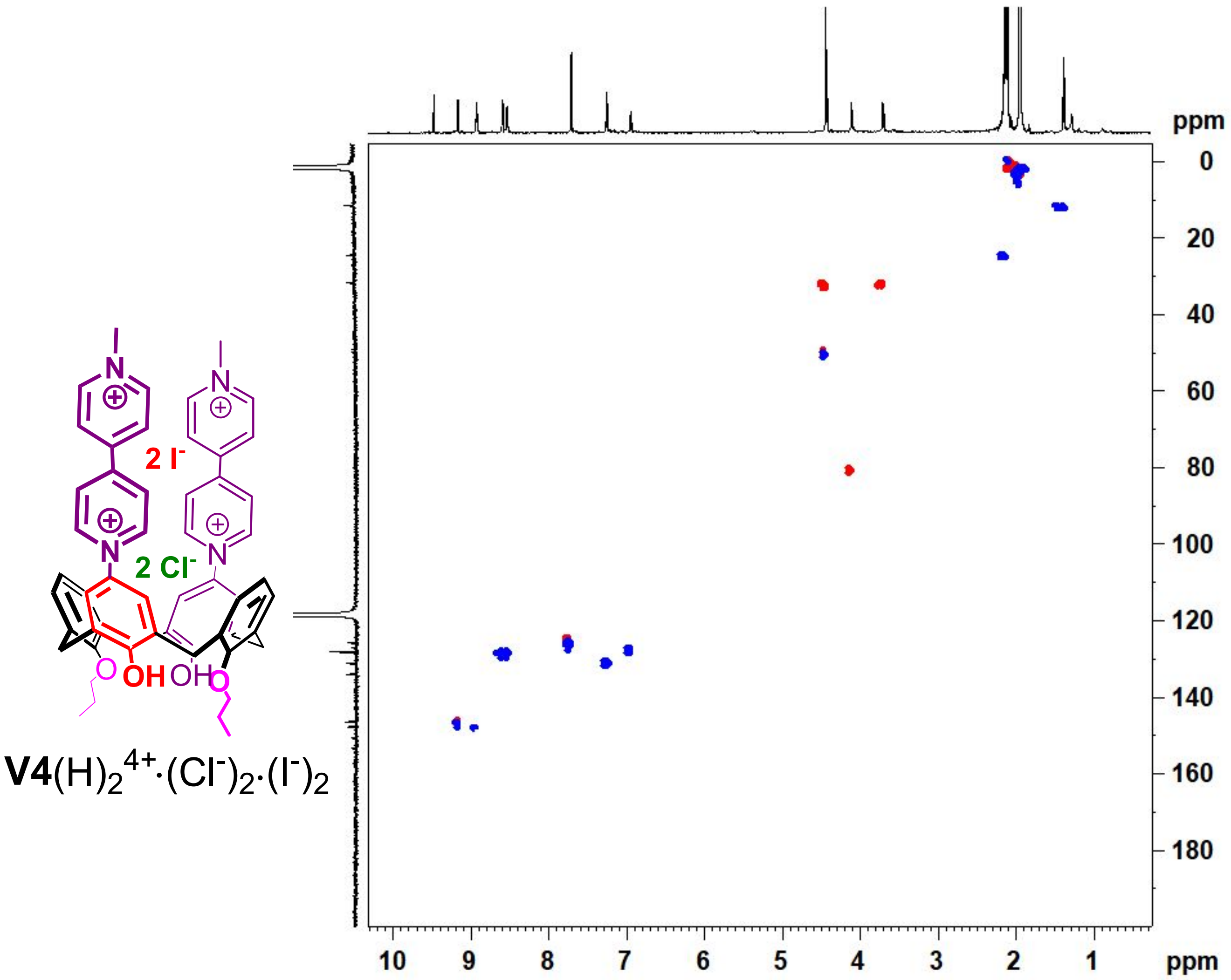

Figure S20. 2D HSQC spectrum of derivative **V4**$(H)_2^{4+}{\cdot}(Cl^-)_2{\cdot}(I^-)_2$ (600 MHz, CD_3CN, 298 K).

^{1}H NMR spectrum of derivative V6(H)$_2^{4+}$·(Cl$^-$)$_2$·(I$^-$)$_2$

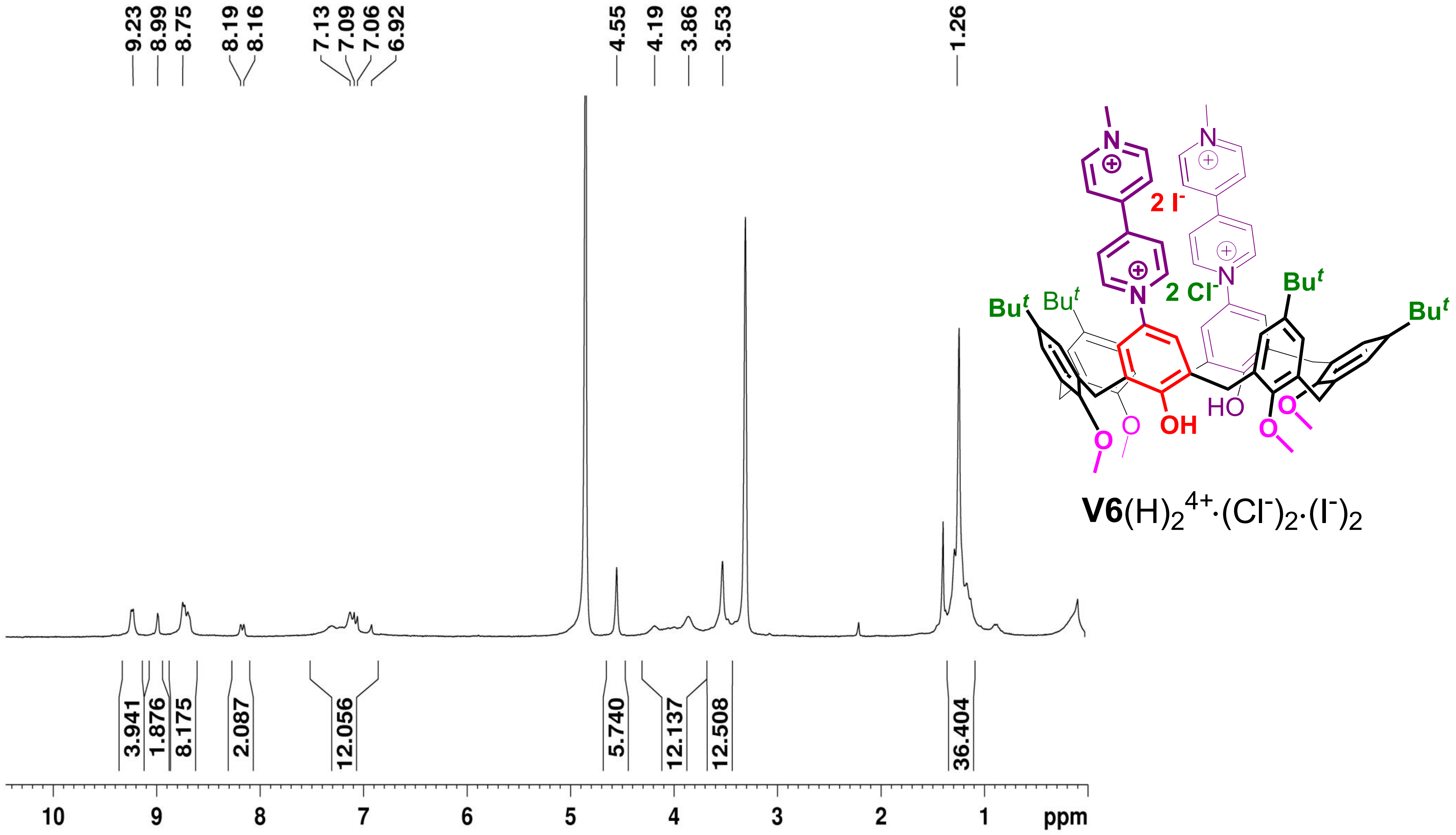

Figure S21. ^{1}H NMR spectrum of derivative **V6**(H)$_2^{4+}$·(Cl$^-$)$_2$·(I$^-$)$_2$ (400 MHz, CD_3OD, 298 K).

$^{13}C\{^1H\}$ NMR spectrum of derivative V6$(H)_2^{4+}\cdot(Cl^-)_2\cdot(I^-)_2$

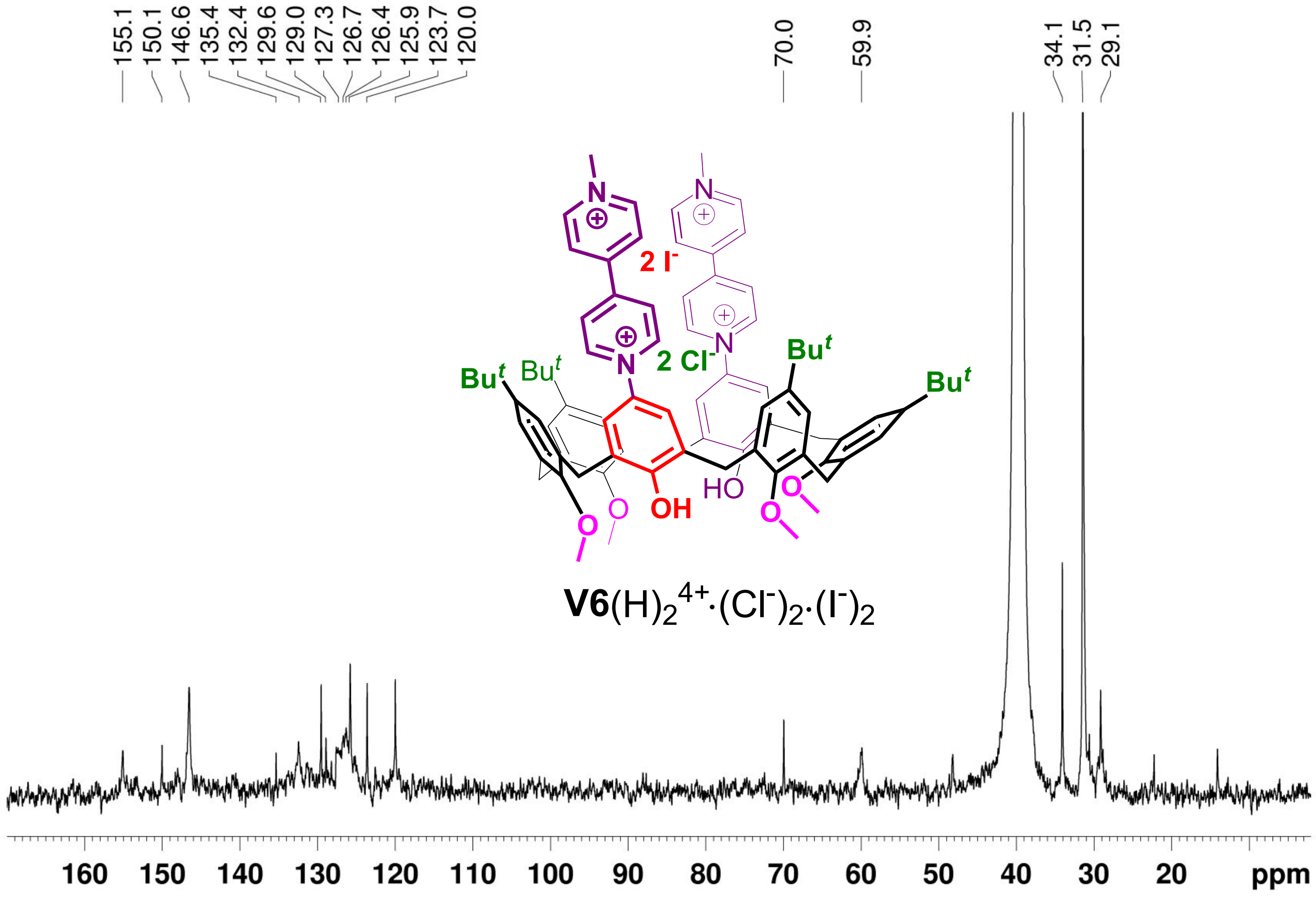

Figure S22. $^{13}C\{^1H\}$ NMR spectrum of derivative **V6**$(H)_2^{4+}\cdot(Cl^-)_2\cdot(I^-)_2$ (150 MHz, $(CD_3)_2SO$, 298 K).

HR-MS spectrum of derivative $V6(H)_2^{4+}{\cdot}(Cl^-)_2{\cdot}(I^-)_2$

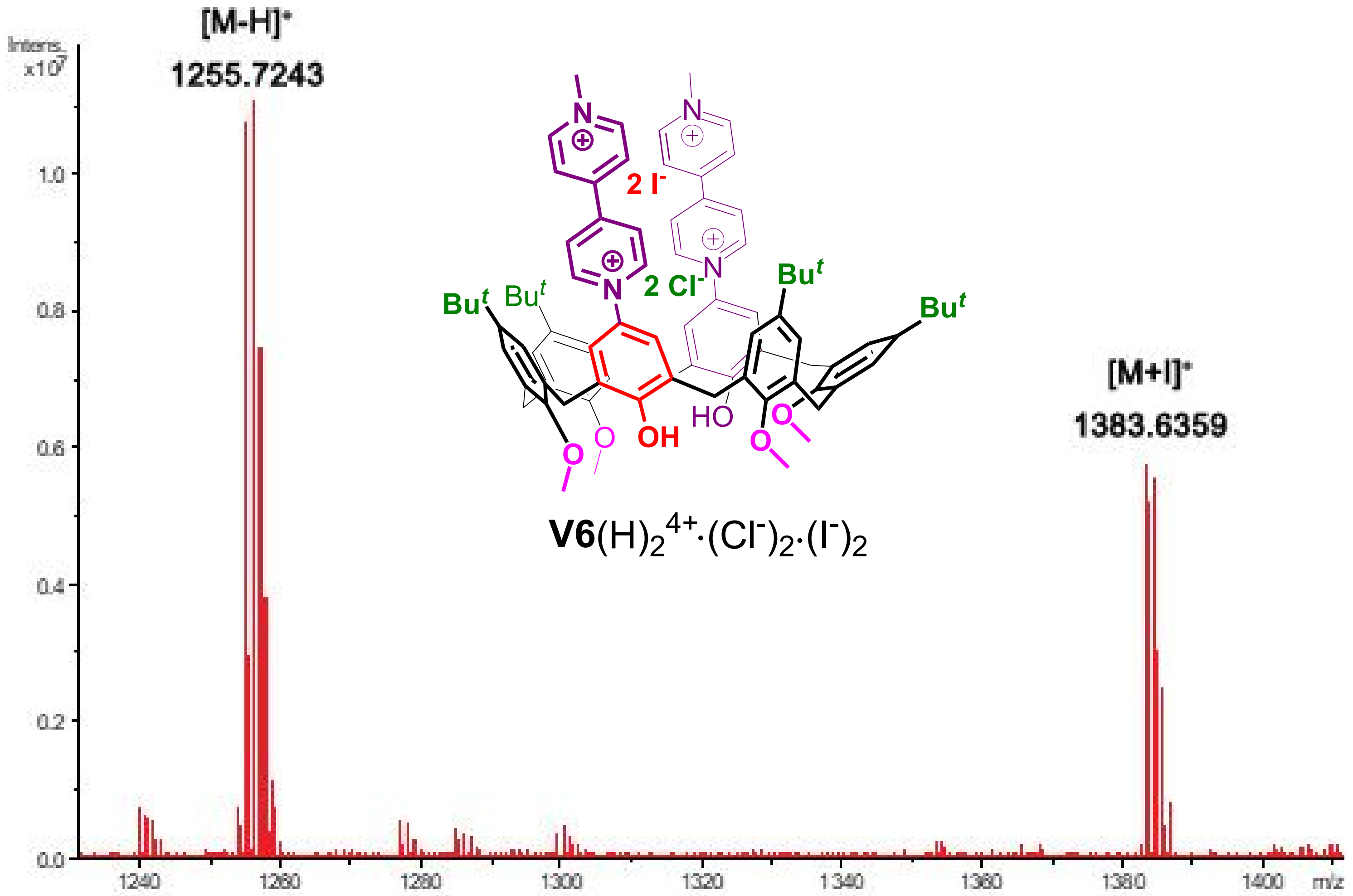

Figure S23. HR-MS spectrum of derivative **V6**$(H)_2^{4+}{\cdot}(Cl^-)_2{\cdot}(I^-)_2$.

^{1}H NMR spectrum of derivative $V6(H)_2^{4+}\cdot(PF_6^-)_4$

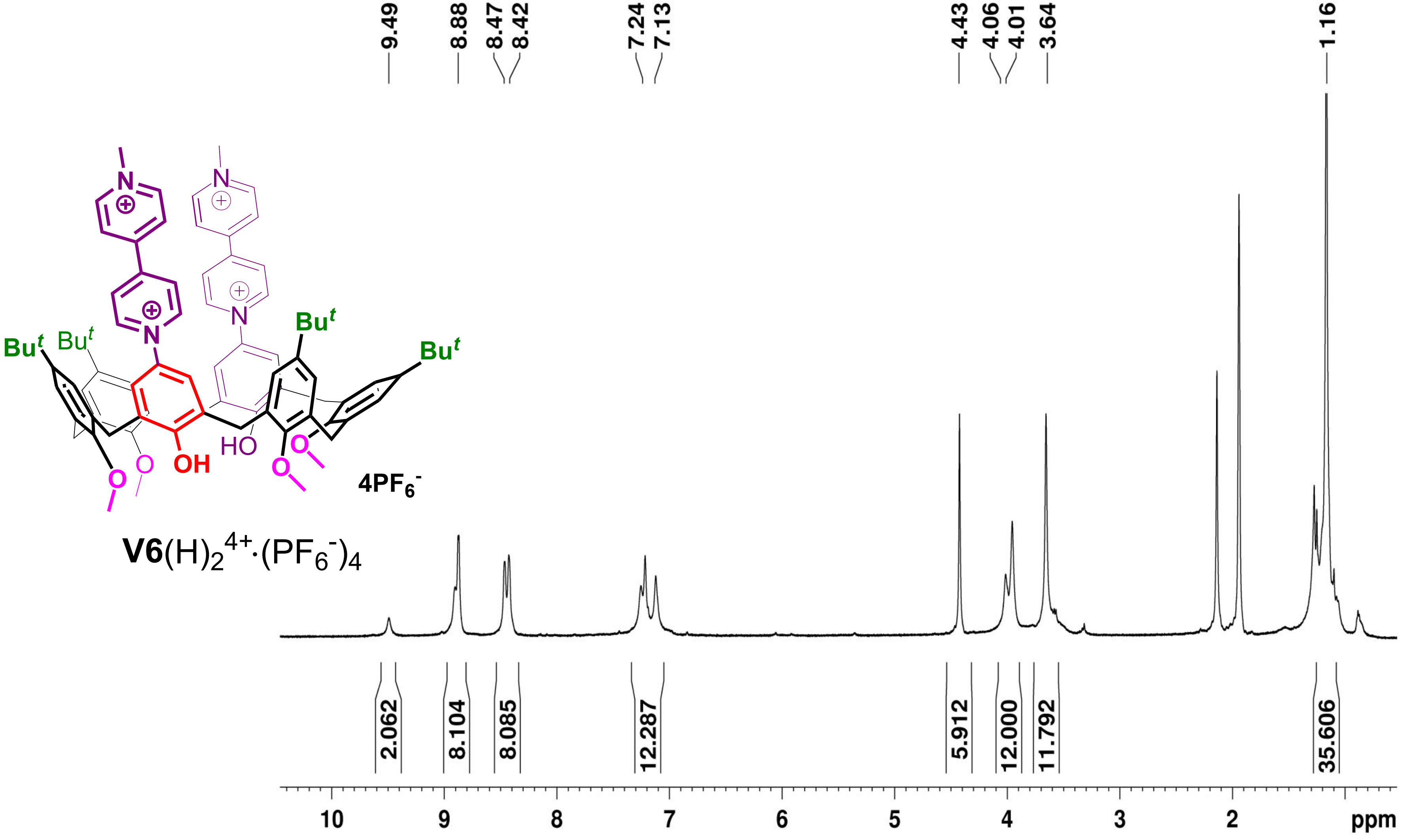

Figure S24. ^{1}H NMR spectrum of derivative **V6**$(H)_2^{4+}\cdot(PF_6^-)_4$ (600 MHz, CD_3CN, 298 K).

$^{13}C\{^1H\}$ NMR spectrum of derivative V6$(H)_2^{4+}\cdot(PF_6^-)_4$

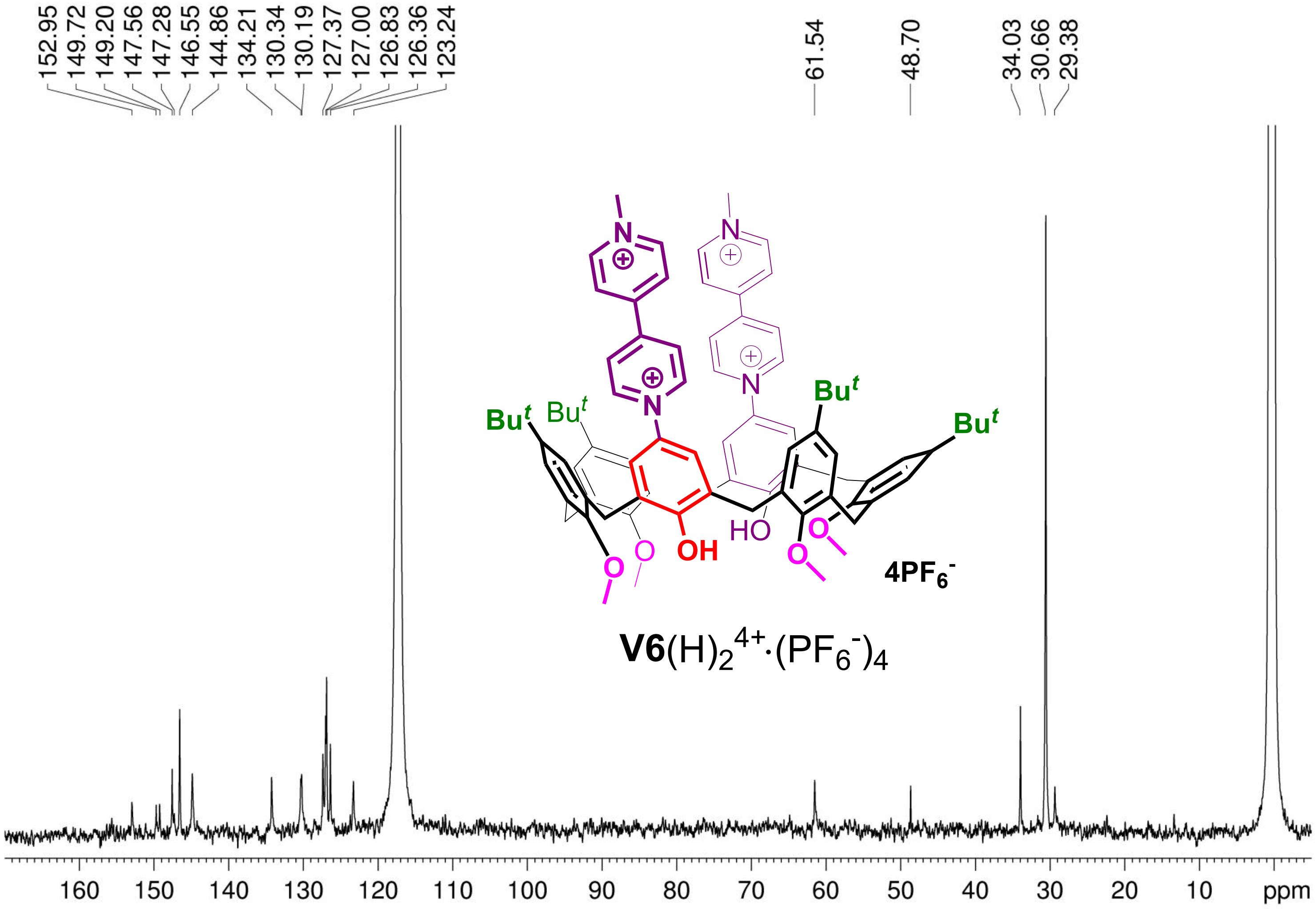

Figure S25. $^{13}C\{^1H\}$ NMR spectrum of derivative **V6**$(H)_2^{4+}\cdot(PF_6^-)_4$ (150 MHz, CD_3CN, 298 K).

HR-MS spectrum of derivative $V6(H)_2^{4+}\cdot(PF_6^-)_4$

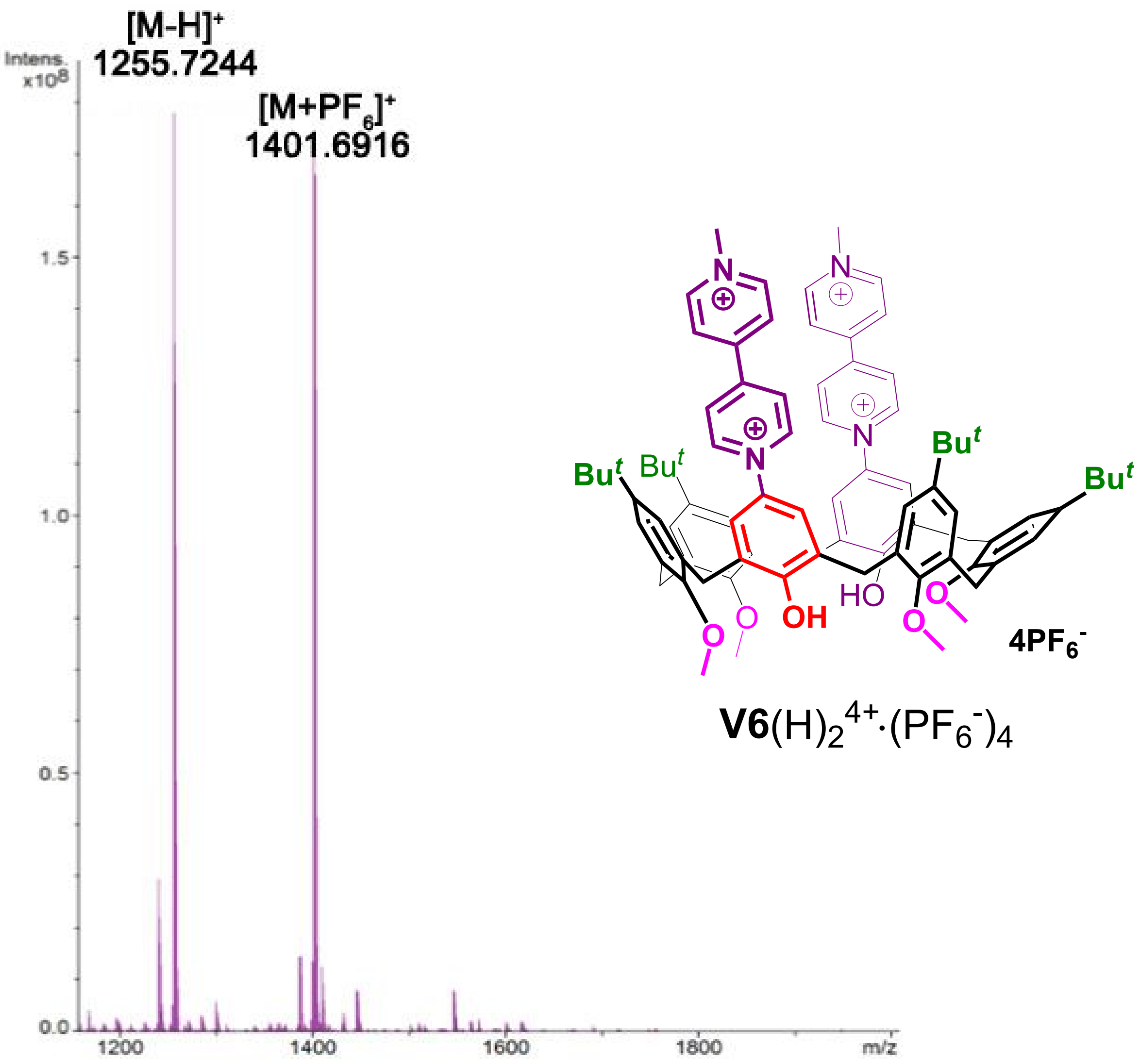

Figure S26. HR-MS spectrum of derivative **V6**$(H)_2^{4+}\cdot(PF_6^-)_4$.

2D HSQC spectrum of derivative V6(H)$_2^{4+}$·(PF$_6^-$)$_4$

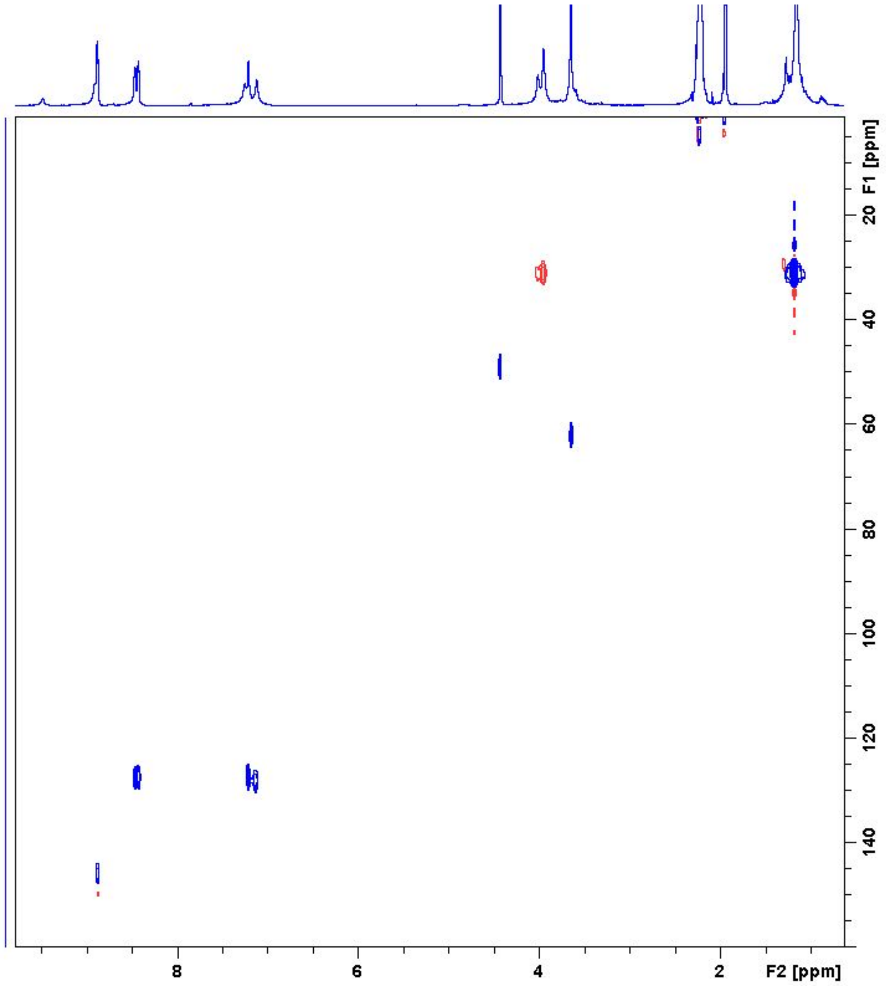

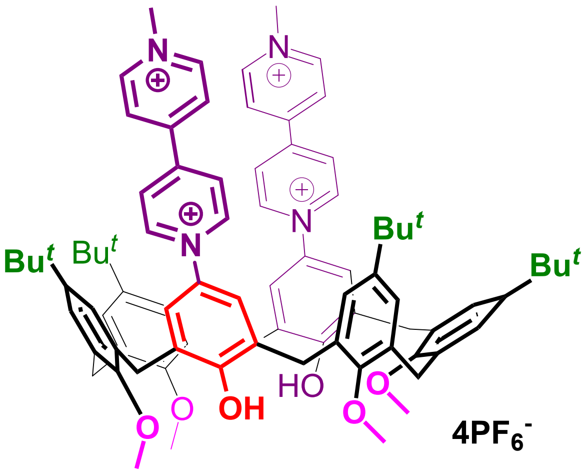

V6(H)$_2^{4+}$·(PF$_6^-$)$_4$

Figure S27. 2D HSQC spectrum of derivative **V6**(H)$_2^{4+}$·(PF$_6^-$)$_4$ (600 MHz, CD$_3$CN, 298 K).

UV-Vis characterization

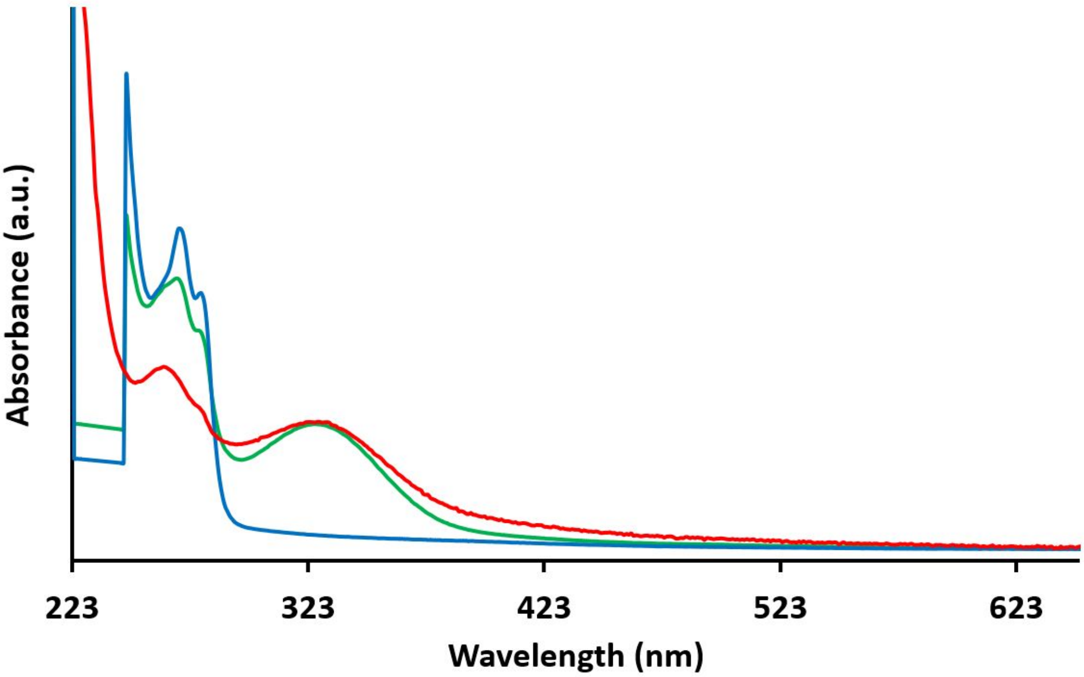

Figure S28. UV/Vis spectra of derivative **P6**$(H)_2^{2+}\cdot(Cl^-)_2$ (red), **P6**$(H)_2^{2+}\cdot(BArF^-)_2$ (green) and NaBArF (blue) in acetonitrile.

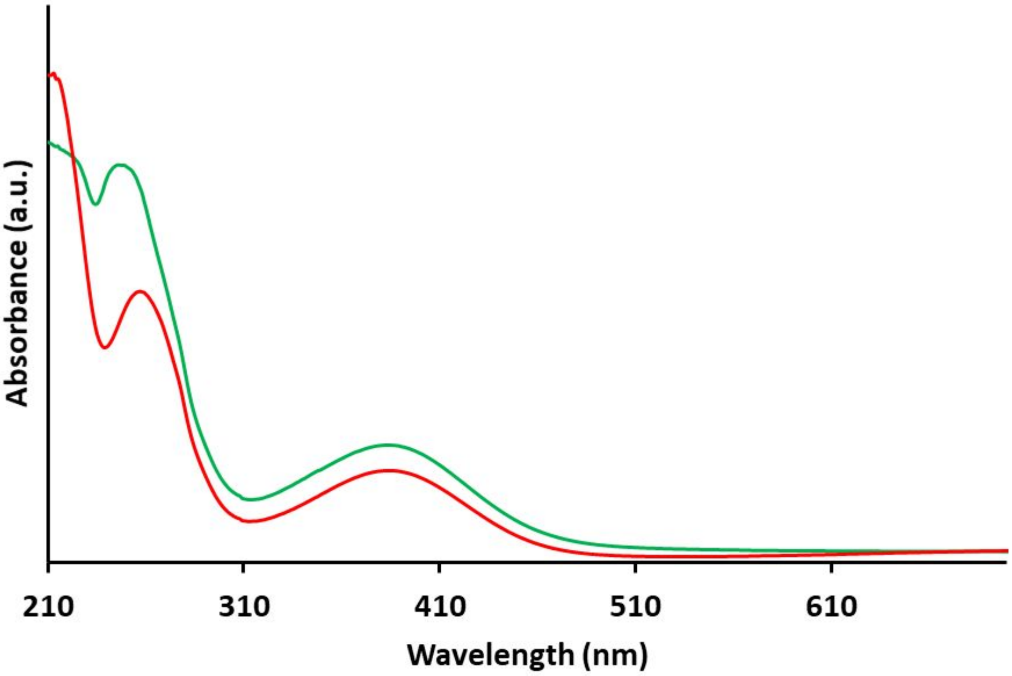

Figure S29. UV/Vis spectra of derivative **V6**$(H)_2^{4+}\cdot(Cl^-)_2\cdot(I^-)_2$ (green) and **V6**$(H)_2^{4+}\cdot(PF_6^-)_4$ (red) in acetonitrile**.**

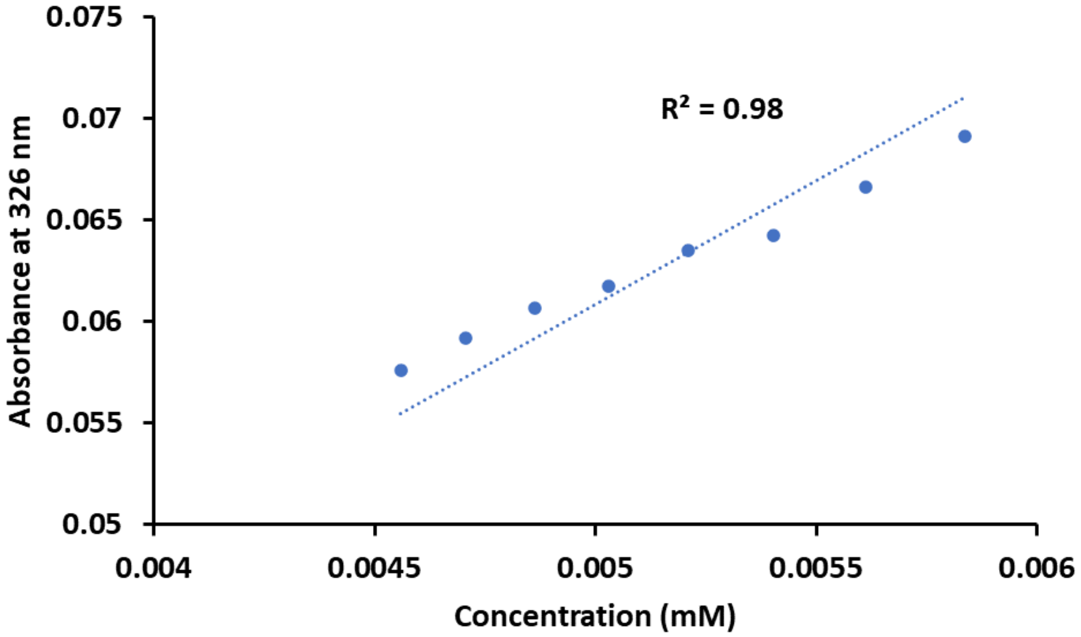

Figure S30. P6$(H)_2^{2+}\cdot(Cl^-)_2$ in acetonitrile, e_{326}=12171 $M^{-1}cm^{-1}$.

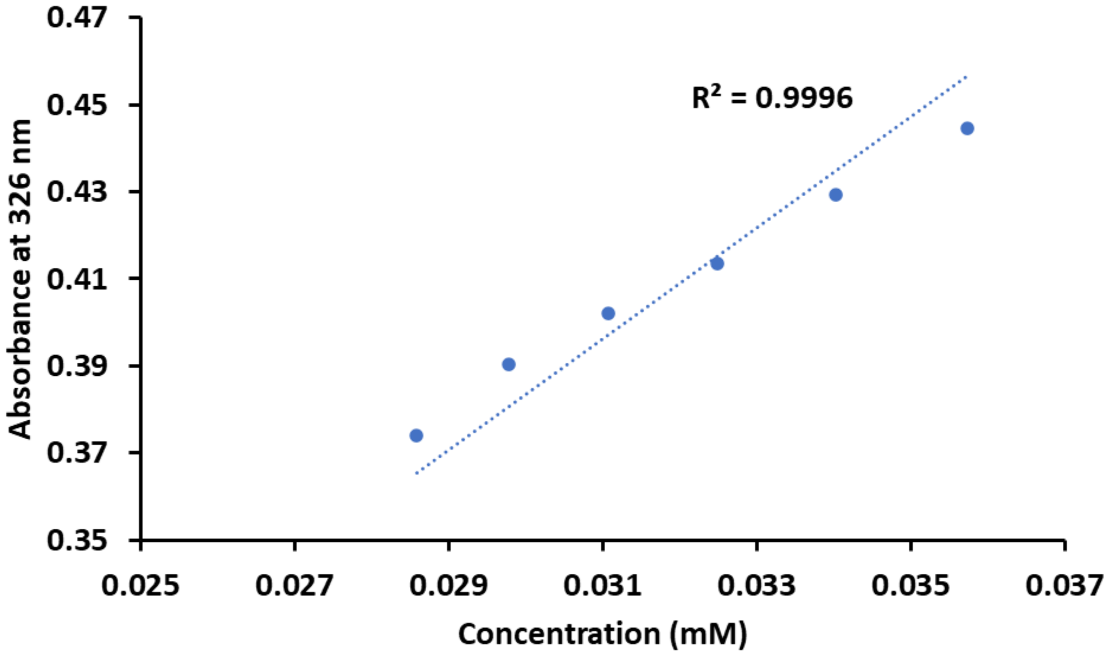

Figure S31. P6$(H)_2^{2+}\cdot(BArF^-)_2$ in acetonitrile, e_{326}=12787 $M^{-1}cm^{-1}$.

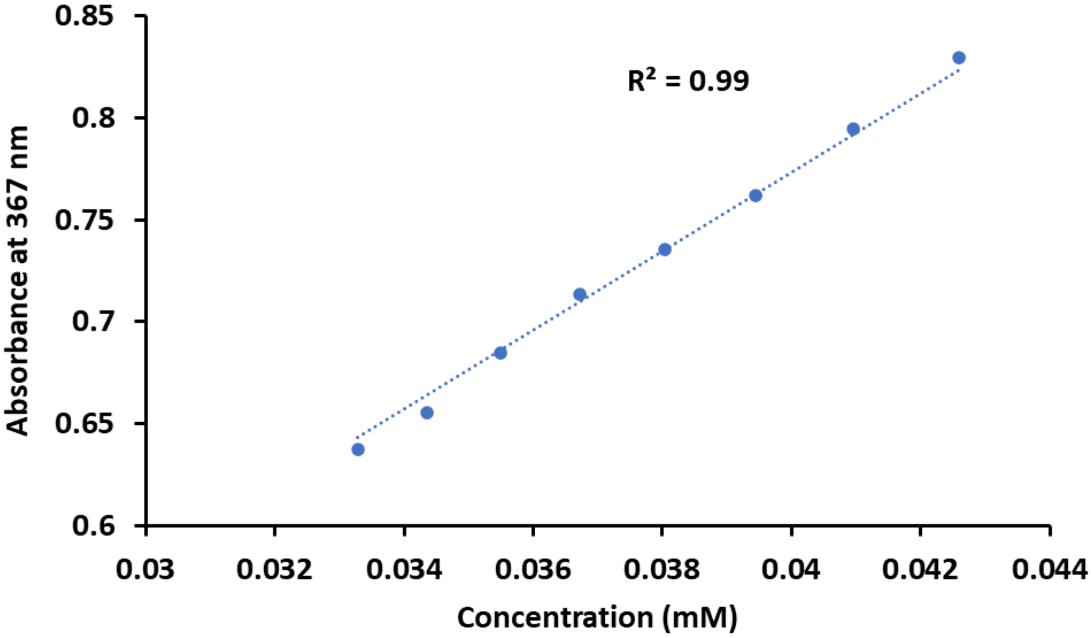

Figure S32. V4$(H)_2^{4+}\cdot(Cl^-)_2\cdot(I^-)_2$ in acetonitrile, e_{367}=19326 $M^{-1}cm^{-1}$.

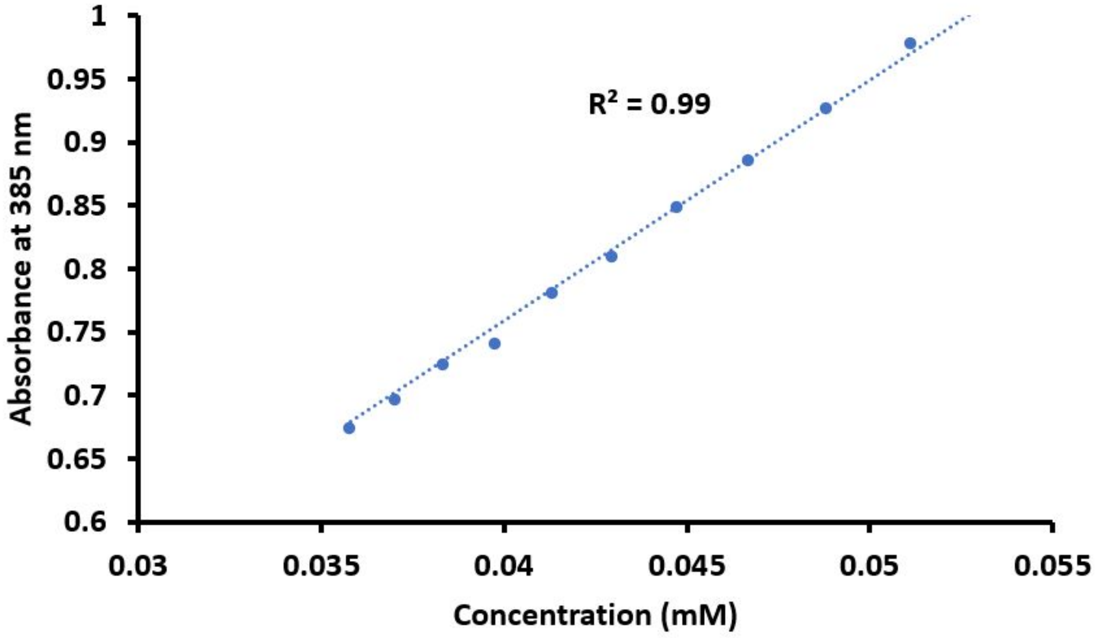

Figure S33. V6$(H)_2^{4+}\cdot(Cl^-)_2\cdot(I^-)_2$ in acetonitrile, e_{385}=18975 $M^{-1}cm^{-1}$.

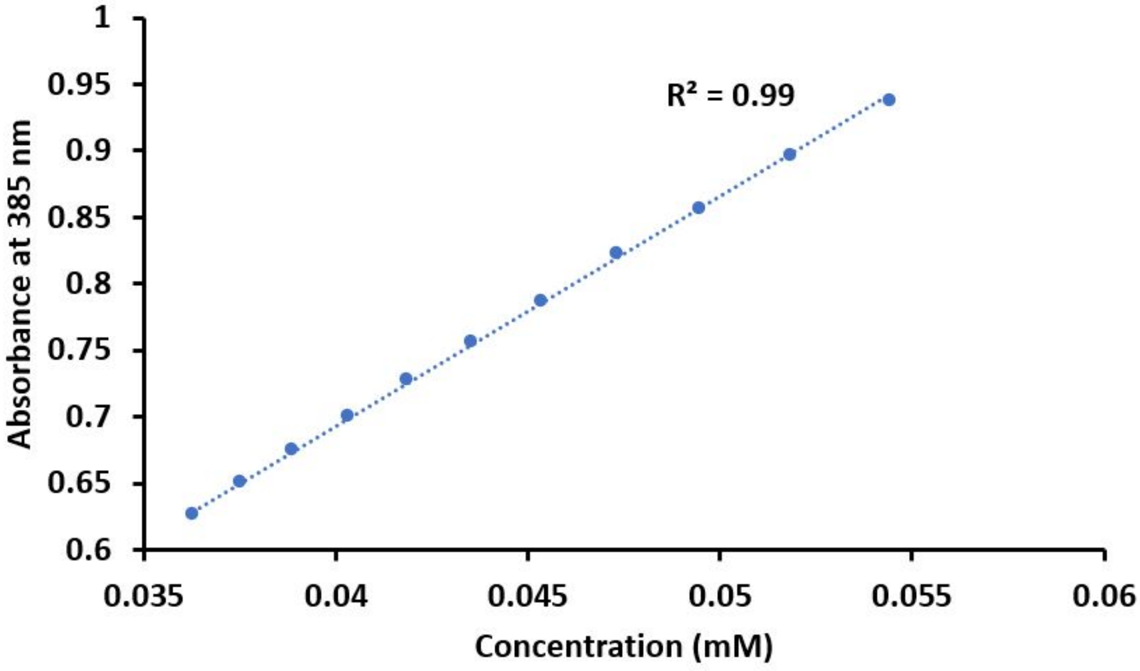

Figure S34. **V6**$(H)_2^{4+}\cdot(PF_6^-)_4$ in acetonitrile, e_{385}= 17324 $M^{-1}cm^{-1}$.

Acid-base UV-VIS titrations

Acetonitrile solutions of **P6**$(H)_2^{2+}\cdot(Cl^-)_2$, **V4**$(H)_2^{4+}\cdot(Cl^-)_2\cdot(I^-)_2$ and **V6**$(H)_2^{4+}\cdot(Cl^-)_2\cdot(I^-)_2$, were prepared in 0.004 mM, concentration, and were titrated with an acetonitrile solution of (n-Bu)$_4$NOH (0.4 mM, 5 ml each point).

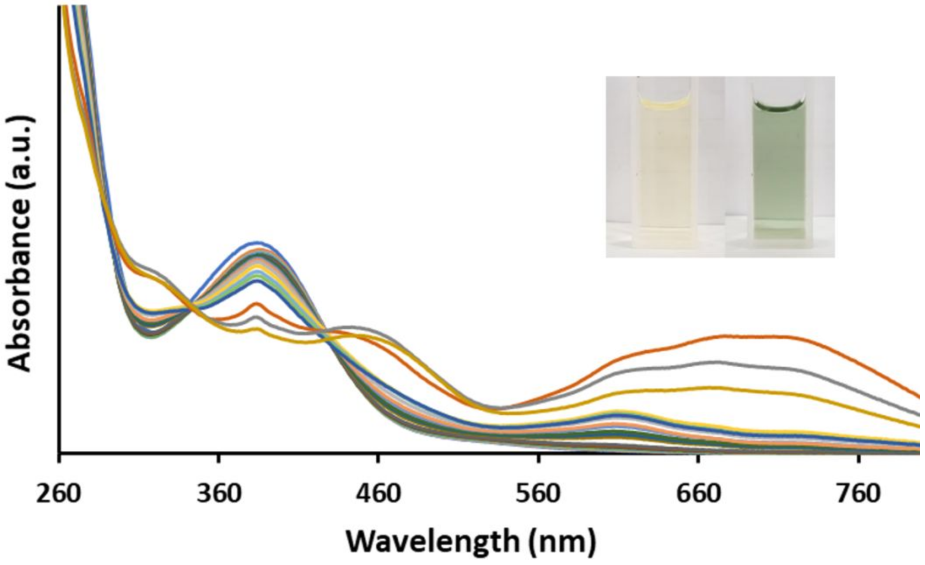

Figure S35. The colour changes of derivative **V4**$(H)_2^{4+}\cdot(Cl^-)_2\cdot(I^-)_2$ upon addition of (nBu)$_4$NOH in acetonitrile (from yellow to green); UV-Vis titration of derivative **V4**$(H)_2^{4+}\cdot(Cl^-)_2\cdot(I^-)_2$ with (n-Bu)$_4$NOH in acetonitrile.

Cation Sensing of derivative $V6(H)_1^{3+}$

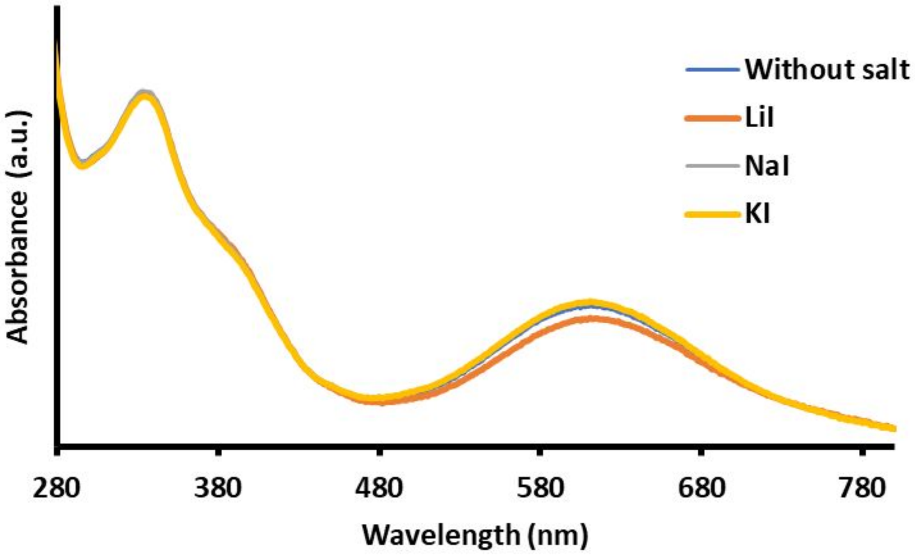

Figure S36. UV/vis spectra of derivative **V6**$(H)_1^{3+}$ in acetonitrile without salt (blue); upon addition of 10 equiv of LiI (orange); upon addition of 10 equiv of NaI (grey); upon addition of 10 equiv of KI (yellow).

Computational details

The lowest energy structure of derivatives **P6**$(H)_2^{2+}\cdot(Cl^-)_2$, **P6**$(H)_2^{2+}$/**P6**$(H)_1^{+}$, **V4**$(H)_2^{4+}$/**V4**$(H)_1^{3+}$ and **V6**$(H)_2^{4+}$/**V6**$(H)_1^{3+}$ was obtained by molecular mechanics calculations performed with YASARA program[1] and AMBER force field.[2] Geometry optimization was carried out at the density functional level of theory (DFT), by using the B3LYP functional in conjunction with the density fitting approximation and the 6-31G(d,p) basis set. For derivatives **P6**$(H)_2^{2+}$/**P6**$(H)_1^{+}$, **V4**$(H)_2^{4+}$/**V4**$(H)_1^{3+}$ and **V6**$(H)_2^{4+}$/**V6**$(H)_1^{3+}$ effects due to solvent (acetonitrile, methanol, and water) polarization were included in computations by the polarizable continuum model (PCM).[3] In addition, for methanol and water solvent one explicit coordinated molecule was included in the starting structure of **V6**$(H)_1^{3+}$. Time dependent (TD) DFT computations were carried out by using the CAM-B3LYP range-separated hybrid functional[4] as previously reported by us.[5] The 6-31G(d,p) basis set was adopted for all the computations employing the CAM-B3LYP functional. DFT and TDDFT computations were carried out by using the Gaussian package (G16).[6]

Cartesian Coordinates of Derivative P6(H)$_2$$^{2+}$·(Cl$^-$)$_2$,

Cartesian Coordinates of the DFT-optimized structure of **P6**(H)$_2$$^{2+}$·(Cl$^-$)$_2$, at the B3lyp/6-31G(d,p) IOp(3/124=3) level of theory

N	-0.0454	3.0989	1.6422
C	-1.2437	3.7216	1.8482
C	-0.2352	5.8825	1.3761
C	1.0577	3.8178	1.2866
C	0.9897	5.224	1.1606
C	-1.3684	5.1201	1.7126
O	-0.3055	1.6669	-4.2708
O	-4.3408	-1.5852	-2.291
O	4.377	-0.9038	-2.3238
O	4.5313	1.1721	1.95
O	0.458	-2.0828	3.8512
O	-4.2461	0.4635	1.9244
C	-4.4494	-0.229	-2.1121
C	1.3561	1.2112	2.491
C	-5.3053	0.2948	-1.113
C	-1.2328	-1.6003	-2.8728
C	1.4875	-0.0633	3.077
C	-5.5043	1.6864	-1.0186
C	0.3383	-0.8664	3.2512
C	-3.7014	0.6547	-2.925
C	4.5863	-0.1855	1.7555
C	3.3106	1.2459	-2.6436
C	-3.9047	2.0476	-2.8032
C	-3.1639	-1.6784	2.243
C	1.0719	-0.0161	-3.2419
C	3.93	-2.4448	2.4127
C	-1.359	-0.3225	-3.4542
C	-0.1982	0.4594	-3.6518
C	0.0222	-2.0962	-2.4349
C	-4.1485	-0.872	1.6224
C	1.1581	-1.2558	-2.5706
C	3.8081	-1.047	2.5629
C	3.3023	2.6361	-2.3939
C	-0.933	-0.4	2.8376
C	4.8098	-3.0095	1.4587
C	-4.8382	2.5832	-1.884
C	4.2856	0.4314	-2.0183
C	5.3991	-0.734	0.734
C	-5.0447	-1.4579	0.6967
C	-1.027	0.8406	2.1682
C	5.1775	1.0083	-1.0826
C	5.5114	-2.1324	0.6043
C	-5.1227	4.0988	-1.8639
C	5.1787	2.4018	-0.8813
C	-5.0396	-2.8523	0.502
C	0.1011	1.6937	2.0362
C	-4.1103	-3.6837	1.1616
C	4.2611	3.2418	-1.5462
C	-3.1468	-3.069	1.9971
C	-4.2047	-5.2126	1.0021
C	5.5474	1.6584	2.8137
C	-5.3314	-2.1223	-3.1545
C	-6.1145	4.5139	-0.7545
C	-3.825	4.8913	-1.6375
C	4.9932	-4.5304	1.3011
C	-5.7316	4.5106	-3.2178
C	-5.3886	-5.7164	1.847
C	-4.4533	-5.6005	-0.4726
C	4.3935	-4.9733	-0.0452
C	6.4943	-4.8884	1.334
C	-2.9343	-5.9416	1.4881
C	4.3124	-5.3395	2.4262
C	4.3628	4.7697	-1.3777
C	3.1042	5.5101	-1.8763
C	4.5904	5.1504	0.1021
C	5.5619	5.2699	-2.203
C	-5.2109	0.7545	2.926
C	5.3444	-1.1963	-3.3223
N	0.1581	-3.5056	-2.0579
C	-0.9419	-4.212	-1.6702
C	0.3157	-6.2947	-1.8363
C	1.3375	-4.1431	-2.3187
C	1.4458	-5.545	-2.2083
C	-0.8893	-5.6208	-1.5648
H	-0.3472	2.3543	-3.5859
H	0.5165	-2.7643	3.1622
H	2.2447	1.8182	2.4616
H	-2.1206	-2.2067	-2.8385
H	-6.1863	2.0475	-0.267
H	-3.3409	2.7135	-3.4409
H	3.3168	-3.0749	3.0393
H	2.1284	-1.5764	-2.2344
H	2.5617	3.2321	-2.9068
H	-1.9997	1.1461	1.826
H	6.1359	-2.5361	-0.1765
H	5.9051	2.8212	-0.2049
H	-5.7707	-3.2795	-0.1644
H	-2.3983	-3.6583	2.5065
H	5.4482	2.7386	2.9182
H	5.4717	1.2161	3.8075
H	6.5447	1.4513	2.4267
H	-5.1809	-3.197	-3.254
H	-6.3381	-1.9618	-2.7701
H	-5.2756	-1.6814	-4.1501

H	-6.2885	5.5904	-0.7634
H	-7.0867	4.0369	-0.8825
H	-5.7355	4.2597	0.2358
H	-4.0096	5.9625	-1.5602
H	-3.3207	4.5738	-0.7247
H	-3.1203	4.7559	-2.4575
H	-5.9627	5.5757	-3.2456
H	-5.0499	4.3087	-4.0445
H	-6.6549	3.9658	-3.4167
H	-5.5013	-6.7985	1.7746
H	-5.2506	-5.4701	2.9007
H	-6.3297	-5.2659	1.5304
H	-4.377	-6.6783	-0.6192
H	-5.4472	-5.3154	-0.8151
H	-3.7364	-5.1201	-1.135
H	4.4892	-6.049	-0.1917
H	3.3314	-4.7306	-0.0832
H	4.8734	-4.4782	-0.8888
H	6.6463	-5.9662	1.2701
H	7.0495	-4.4436	0.5094
H	6.9555	-4.548	2.2619
H	-2.9835	-7.0134	1.2969
H	-2.0398	-5.5623	0.997
H	-2.7824	-5.8262	2.5617
H	4.4925	-6.4086	2.3149
H	4.6798	-5.0483	3.4105
H	3.2301	-5.2086	2.4217
H	3.1623	6.5816	-1.6856
H	2.9601	5.395	-2.9509
H	2.2029	5.1407	-1.3903
H	4.5297	6.2291	0.249
H	3.8514	4.6829	0.7491
H	5.5723	4.8477	0.4636
H	5.6804	6.3509	-2.1242
H	6.4954	4.812	-1.8748
H	5.4375	5.0291	-3.2596
H	-6.1657	0.2607	2.7456
H	-4.86	0.4499	3.9116
H	-5.3911	1.8289	2.9534

H	5.5012	-2.2737	-3.3694
H	5.0103	-0.8642	-4.3048
H	6.3078	-0.7275	-3.1221
H	-0.3116	6.9534	1.2463
H	0.3802	-7.3684	-1.7242
C	6.1311	0.1532	-0.2613
H	6.837	0.783	0.2768
H	6.7394	-0.4537	-0.9289
C	2.8514	-0.4996	3.6149
H	3.3137	0.3494	4.1147
H	2.7267	-1.2434	4.4021
C	-2.2096	-1.1181	3.2934
H	-2.759	-0.4147	3.9148
H	-1.9633	-1.9257	3.9821
C	-6.0137	-0.6124	-0.1169
H	-6.628	-0.0215	0.5593
H	-6.7131	-1.2496	-0.6548
C	-2.7251	0.1337	-3.9776
H	-2.599	0.8958	-4.7466
H	-3.1842	-0.7022	-4.5019
C	2.3546	0.6929	-3.6973
H	2.9	-0.0143	-4.3179
H	2.1151	1.5031	-4.3852
H	1.8546	5.7908	0.8525
H	1.9576	3.272	1.0526
H	-1.7492	-6.1811	-1.233
H	-1.8249	-3.6538	-1.4012
H	2.1785	-3.5598	-2.6497
H	2.3879	-6.037	-2.4019
H	-2.3249	5.5999	1.8606
H	-2.0896	3.1314	2.1522
Cl	0.6172	-3.9854	1.3357
Cl	-0.4119	3.5359	-1.7529

Energy = -4275.628208 a.u.

0 imaginary frequency

Derivatives P6(H)$_2^{2+}$/P6(H)$_1^{+}$

TD-DFT calculations on derivative **P6**(H)$_2^{2+}$ predicted the S1 ← S0 transition at **288 nm** (experimental data = **326 nm**).

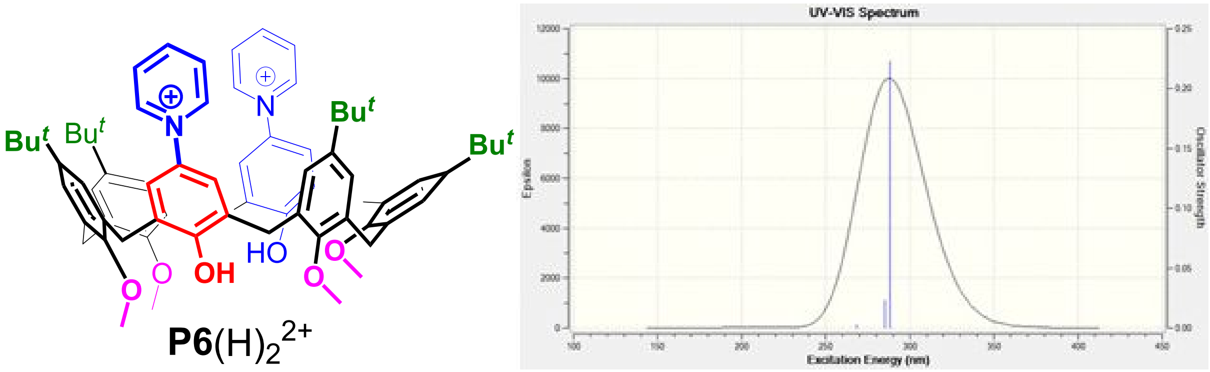

Figure S37. TD-DFT calculated UV-VIS spectrum of derivative **P6(H)$_2^{2+}$** in acetonitrile**.**

TD-DFT calculations on derivative **P6**(H)$_2^{1+}$ predicted the S1 ← S0 transition at **437 nm** (experimental data = **487 nm**).

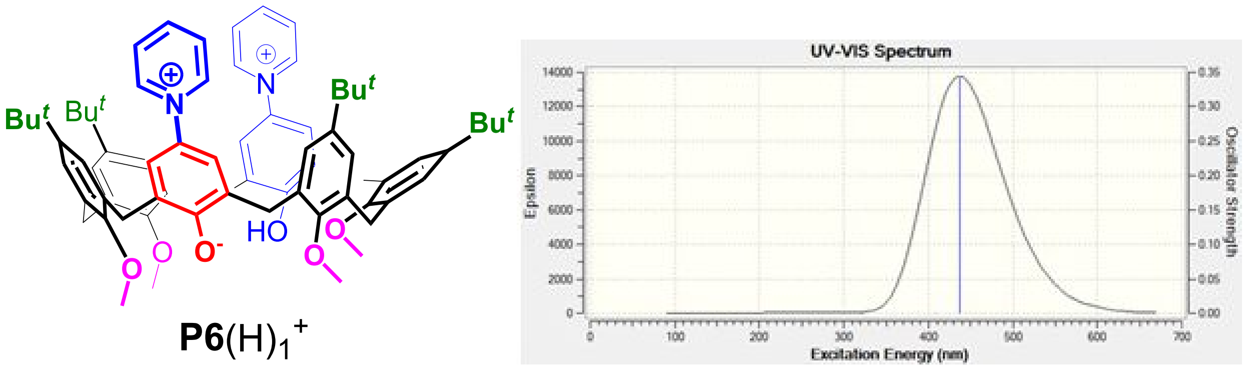

Figure S38. TD-DFT calculated UV-VIS spectrum of derivative **P6**(H)$_1^+$ in acetonitrile**.**

Atomic coordinate of derivative P6(H)$_2^{2+}$ in acetonitrile

N	0.10573400	2.83202200	3.51885600
C	0.96552200	3.50881700	4.31203900
C	-0.02105700	5.57538500	3.63395400
C	-0.81734000	3.48980300	2.78330800
C	-0.90721100	4.86314700	2.83441100
C	0.92933500	4.88399800	4.37554200
O	-0.48439400	2.12952000	-4.64784100
O	-4.41546700	-0.94513600	-2.35181500
O	4.34042500	-0.27212400	-2.76853400
O	4.22507700	0.77904000	2.16543600
O	0.36249900	-2.71992700	3.36876300
O	-3.92435600	0.45685700	2.43858800
C	-4.24951000	0.38125100	-1.99101600
C	1.38698100	0.74782000	3.38549300
C	-4.79101600	0.83615800	-0.78916300
C	-1.27081900	-1.01939900	-3.06253400
C	1.45301300	-0.63948200	3.38458400
C	-4.62817300	2.18199400	-0.45548200
C	0.24978600	-1.36447000	3.41896900
C	-3.50857200	1.23219200	-2.81716400
C	4.25694200	-0.47772200	1.58346300
C	3.02193900	1.73474900	-2.86369900
C	-3.37739600	2.56636500	-2.44873800
C	-3.13597600	-1.81034800	2.55806800
C	1.01729900	0.46126300	-3.73151600
C	3.69459200	-2.80934200	1.62364600
C	-1.41719200	0.19006500	-3.72535600
C	-0.26792000	0.93533400	-4.03797800
C	-0.00263300	-1.46687400	-2.73015400
C	-3.92664300	-0.83609400	1.94412500

C	1.13452300	-0.75434200	-3.06087800
C	3.57936100	-1.53661800	2.18869100
C	2.69450100	2.96759900	-2.30960700
C	-0.99485400	-0.72397300	3.54473100
C	4.45477500	-3.04771400	0.48000300
C	-3.94665200	3.07639600	-1.27898100
C	4.00358500	0.96081200	-2.23813600
C	4.96447000	-0.65926700	0.39071100
C	-4.71804700	-1.14602800	0.83273100
C	-1.01920000	0.66534700	3.57278600
C	4.60909800	1.38654000	-1.05603000
C	5.05338600	-1.94407500	-0.13209600
C	-3.81825400	4.57090000	-0.96007800
C	4.24893900	2.62910400	-0.53490000
C	-4.74052200	-2.46402900	0.38919900
C	0.15911900	1.38514900	3.47262500
C	-3.99961500	-3.47850100	0.99737500
C	3.30353200	3.44858400	-1.14796600
C	-3.18784700	-3.11729800	2.07039500
C	-4.13632900	-4.92203400	0.49805200
C	5.31302900	1.02712200	3.05542500
C	-5.55915200	-1.18554700	-3.16897000
C	-4.45025200	4.93407000	0.38840800
C	-2.33303200	4.96955600	-0.92422700
C	4.68128900	-4.44832100	-0.10339300
C	-4.53021200	5.38243300	-2.05773000
C	-5.59196900	-5.38459300	0.69272200
C	-3.78181100	-4.99225800	-0.99724300
C	4.14917500	-4.50558200	-1.54576800
C	6.19120100	-4.75099100	-0.11335800
C	-3.22285300	-5.88908800	1.25937700
C	3.98833200	-5.54059000	0.71851900
C	2.91505500	4.82478800	-0.59427000
C	1.40414900	4.85223000	-0.30774800
C	3.65376100	5.15737500	0.70729300
C	3.25585300	5.90694400	-1.63410900
C	-4.94463100	0.71127800	3.40457700
C	5.41021300	-0.23255300	-3.71125200
H	1.65200600	2.91067600	4.89416500
H	-1.65893500	5.36366600	2.23834000
H	-1.45569700	2.87679900	2.16185800
H	1.63086300	5.39816900	5.01913900
N	0.12121800	-2.70785700	-1.98275100
C	0.30026800	-2.64901600	-0.64696500
C	0.27634200	-5.03203400	-0.54511200
C	0.02197700	-3.88939000	-2.62654500
C	0.09912600	-5.07261600	-1.92440200
C	0.38032900	-3.80419800	0.10037900
H	0.36481200	-1.65777900	-0.21893900
H	0.01851400	-6.01041100	-2.45808100
H	-0.11660900	-3.83782700	-3.69793000
H	0.51584000	-3.72246400	1.17227400
H	0.34917000	2.57309600	-4.84408800
H	-0.49740100	-3.13858200	3.50020600
H	2.30833300	1.31065400	3.30064200
H	-2.15392100	-1.58575200	-2.78625200
H	-5.06424200	2.52269400	0.47556600
H	-2.80902500	3.21775900	-3.10540700
H	3.19359500	-3.62825600	2.12666500
H	2.12088300	-1.12941100	-2.80791600
H	1.93703800	3.56698600	-2.80623400
H	-1.97043700	1.16788000	3.69365500
H	5.62218800	-2.07788100	-1.04560000
H	4.72733900	2.94290200	0.38387800
H	-5.37426400	-2.69784500	-0.45868500
H	-2.60204000	-3.87522100	2.57865100
H	5.18653700	2.04247000	3.43208500
H	5.30966800	0.32527200	3.89555900
H	6.27546100	0.94975300	2.54069300
H	-5.59549800	-2.25968600	-3.35304100
H	-6.47960400	-0.87261100	-2.66620900
H	-5.48603800	-0.65632800	-4.12471500
H	-4.32078200	6.00320700	0.57794100
H	-5.52334100	4.72430000	0.40396300
H	-3.98828700	4.38908400	1.21698100
H	-2.22974300	6.03067600	-0.67693200
H	-1.78813200	4.38867400	-0.17466600
H	-1.84319300	4.80606300	-1.88722500
H	-4.44381800	6.45430700	-1.85390400
H	-4.09746900	5.19256500	-3.04317300
H	-5.59347000	5.12959100	-2.10217800
H	-5.71198400	-6.41387700	0.34074500
H	-5.87426000	-5.35151000	1.74898100
H	-6.29295600	-4.75698100	0.13681400
H	-3.88345800	-6.01884600	-1.36209400
H	-4.43574900	-4.35861900	-1.60126800
H	-2.75143800	-4.67172600	-1.17109500
H	4.32611300	-5.49581100	-1.97666000
H	3.07365300	-4.31246800	-1.57538300
H	4.63895300	-3.76950400	-2.18812400
H	6.37306700	-5.74989100	-0.52178600
H	6.74509900	-4.03471000	-0.72491300
H	6.60233400	-4.71831800	0.89974200
H	-3.34155900	-6.89869800	0.85723200
H	-2.16862400	-5.61521500	1.16287000
H	-3.46890300	-5.92727600	2.32429400
H	4.18149000	-6.51734600	0.26687700
H	4.36063600	-5.57039100	1.74637000
H	2.90488500	-5.40224100	0.75391700
H	1.11200300	5.82416000	0.10188500
H	0.81373700	4.68150100	-1.21097900
H	1.13440100	4.08152600	0.42015300
H	3.34241200	6.14402200	1.06111400
H	3.42870200	4.43543900	1.49798900
H	4.73771800	5.18392900	0.56550900
H	2.97473600	6.89567800	-1.25847700
H	4.32831000	5.91763700	-1.84913800
H	2.72532100	5.74425100	-2.57574300

H	-5.94203000	0.54440400	2.98765900
H	-4.82714200	0.07566800	4.28806200
H	-4.84702900	1.75702800	3.69699100
H	5.60075600	-1.26227100	-4.01452800
H	5.14474600	0.36221100	-4.59147800
H	6.31666500	0.18855400	-3.26550900
H	-0.07269500	6.65657700	3.68044100
H	0.33334400	-5.95425500	0.02154500
C	5.60978700	0.50930900	-0.32915600
H	6.16031000	1.13391300	0.37687100
H	6.34403200	0.11205100	-1.03315300
C	2.79680000	-1.34366200	3.47484800
H	3.40658500	-0.77427400	4.18088200
H	2.63487200	-2.32395900	3.92441300
C	-2.28864400	-1.48817000	3.77672200
H	-2.87944800	-0.90163400	4.48307700
H	-2.08135900	-2.42523000	4.30524300
C	-5.55836800	-0.09459400	0.13103900
H	-6.07946200	0.51957400	0.86745300
H	-6.33392100	-0.61434200	-0.43707000
C	-2.80421400	0.70722500	-4.05248100
H	-2.73007400	1.50198400	-4.79414400
H	-3.37873000	-0.10588700	-4.49849000
C	2.28711800	1.21826900	-4.08485300
H	2.93096400	0.54454300	-4.65175000
H	2.08621500	2.05706300	-4.75686100

Energy = -3353.096264 a.u.

0 imaginary frequency

Atomic coordinate of derivative P6(H)$_1^+$ in acetonitrile

N	-0.11672600	2.77240800	-3.44639500
C	-1.05687400	3.46082200	-4.13685500
C	-0.00232500	5.53336000	-3.57684100
C	0.87917600	3.44208300	-2.81883000
C	0.96836900	4.81520400	-2.88951300
C	-1.03272700	4.83667800	-4.19644800
O	0.47363800	2.12870200	4.67877100
O	4.44826500	-0.93252500	2.37719700
O	-4.37450200	-0.23379000	2.76501600
O	-4.26015400	0.72096800	-2.16297100
O	-0.24904000	-2.77195000	-3.29889300
O	3.99641300	0.40697600	-2.40597600
C	4.28942700	0.38751200	1.99106100
C	-1.38265500	0.66972800	-3.32879500
C	4.85037200	0.82235400	0.79051700
C	1.26548200	-0.93879600	2.94096000
C	-1.43856300	-0.70946900	-3.31387000
C	4.68986500	2.16210200	0.43221900
C	-0.22593300	-1.49923500	-3.33725000
C	3.53080200	1.24933200	2.78882900
C	-4.28530800	-0.52649300	-1.55920800
C	-3.04473000	1.76565800	2.80978300
C	3.40311000	2.57699000	2.39645400
C	3.16277400	-1.85171600	-2.49095300
C	-1.02415300	0.51404500	3.66420900
C	-3.71455000	-2.85152100	-1.53561500
C	1.40993000	0.23935500	3.65903900
C	0.25976100	0.96847600	4.00436100
C	-0.00156200	-1.37300100	2.58757000
C	3.97777600	-0.88338600	-1.89924900
C	-1.13884900	-0.67301000	2.94367000
C	-3.58983100	-1.59287000	-2.13195100
C	-2.71461700	2.98621100	2.23051400
C	1.01657700	-0.76818200	-3.45704600
C	-4.49539300	-3.06892800	-0.40148300
C	3.99127700	3.06844600	1.22826100
C	-4.03774600	0.98755100	2.20840400
C	-5.00895900	-0.68291900	-0.37236200
C	4.77645600	-1.18585900	-0.79068300
C	1.02915300	0.60936300	-3.48442500
C	-4.65084700	1.39398100	1.02307000
C	-5.10691700	-1.95518200	0.17815600
C	3.85973400	4.55599300	0.87910400
C	-4.28756200	2.62462000	0.47656200
C	4.79174000	-2.49721300	-0.32808800
C	-0.15885800	1.33683600	-3.39745700
C	4.02793300	-3.50850600	-0.91369300
C	-3.33137800	3.44936700	1.06606600
C	3.20129200	-3.15019600	-1.97675000
C	4.15123600	-4.94642200	-0.39355900
C	-5.32208600	0.92698900	-3.09016500
C	5.57809000	-1.15975500	3.21544100
C	4.51047400	4.89748100	-0.46626400
C	2.37294300	4.94587100	0.81316300
C	-4.72900300	-4.45787700	0.20687300
C	4.55054900	5.39210700	1.97180000
C	5.60729000	-5.41941800	-0.55796300
C	3.77232000	-4.99756400	1.09664700
C	-4.23263900	-4.48261000	1.66289000
C	-6.23717400	-4.76862100	0.18642100
C	3.24822000	-5.92039900	-1.15825800
C	-4.01005100	-5.56377200	-0.57319700
C	-2.93886500	4.81290200	0.48424300
C	-1.42760400	4.83368400	0.19744700
C	-3.67715100	5.12026600	-0.82371600
C	-3.27811200	5.91609400	1.50238900
C	4.99272000	0.62061800	-3.40145500
C	-5.43556800	-0.17173000	3.71512100
H	-1.79692500	2.86632000	-4.65170300
H	1.78568100	5.31190500	-2.38273000
H	1.57491700	2.83476500	-2.25758800
H	-1.80626200	5.34770100	-4.75506300

N	-0.12604000	-2.58604500	1.79584400
C	-0.20011900	-2.48718500	0.45205300
C	-0.29472800	-4.85909700	0.28539200
C	-0.13697400	-3.78606800	2.41280500
C	-0.22208800	-4.94489100	1.67291600
C	-0.28614800	-3.61511500	-0.33756500
H	-0.18150700	-1.48355900	0.04852000
H	-0.22914100	-5.89885900	2.18389300
H	-0.07465200	-3.76758800	3.49232500
H	-0.33456400	-3.48765200	-1.42142500
H	-0.36178500	2.55434300	4.90420200
H	-2.31097400	1.22452000	-3.25058000
H	2.14864600	-1.49613200	2.64649400
H	5.13712900	2.48701500	-0.49910600
H	2.81998900	3.23756800	3.03090800
H	-3.19490500	-3.67777700	-2.00625300
H	-2.12327200	-1.03700400	2.66851400
H	-1.94668100	3.58801400	2.70748200
H	1.97809200	1.11863800	-3.61147700
H	-5.68951600	-2.06984300	1.08574400
H	-4.76944300	2.92260600	-0.44560300
H	5.43157800	-2.72642800	0.51693300
H	2.56810400	-3.88910700	-2.45267300
H	-5.20655200	1.93737500	-3.48432500
H	-5.27735900	0.20940600	-3.91587500
H	-6.29949900	0.83906100	-2.60446000
H	5.61154100	-2.23083200	3.41759800
H	6.50673800	-0.85522800	2.72237400
H	5.49007000	-0.61515200	4.16149800
H	4.37861100	5.96232700	-0.67752100
H	5.58476700	4.69317200	-0.46147100
H	4.06428300	4.33478600	-1.29159300
H	2.26862200	6.00124700	0.54244300
H	1.84154000	4.34733600	0.06803800
H	1.87045800	4.80011300	1.77246900
H	4.46105000	6.45962100	1.74717800
H	4.10425500	5.21760800	2.95408500
H	5.61454400	5.14666400	2.03677300
H	5.71604100	-6.44580000	-0.19343300
H	5.90752900	-5.39912800	-1.60970400
H	6.30319700	-4.79038600	0.00261000
H	3.87408600	-6.01771600	1.48004300
H	4.41229300	-4.34899400	1.70022900
H	2.73697900	-4.68105100	1.24842700
H	-4.41562200	-5.46472000	2.11009900
H	-3.15923400	-4.28256500	1.71527600
H	-4.74193900	-3.73601100	2.27744800
H	-6.42541700	-5.75906500	0.61263100
H	-6.80907700	-4.04097500	0.76738600
H	-6.62320700	-4.76059600	-0.83709100
H	3.36643300	-6.92824600	-0.75072200
H	2.19279900	-5.64946000	-1.07312200
H	3.50346100	-5.95962800	-2.22090300
H	-4.20788100	-6.53186200	-0.10476500
H	-4.35650700	-5.61850100	-1.60902600
H	-2.92737700	-5.41693900	-0.58508600
H	-1.13626100	5.79681700	-0.23299900
H	-0.83712400	4.68333500	1.10435500
H	-1.15588700	4.04789400	-0.51309900
H	-3.36157100	6.09734400	-1.19985600
H	-3.45594700	4.37968000	-1.59792100
H	-4.76089500	5.15502100	-0.68162300
H	-2.99500800	6.89696400	1.10789900
H	-4.35065600	5.93307200	1.71692800
H	-2.74819500	5.77023100	2.44719700
H	5.99766700	0.42807800	-3.01188400
H	4.83088900	-0.01894800	-4.27518300
H	4.91974900	1.66616400	-3.70347500
H	-5.62636200	-1.19426600	4.04201600
H	-5.16125300	0.44074600	4.58059700
H	-6.34516600	0.24202200	3.26854500
H	0.04445600	6.61427400	-3.63011800
H	-0.35793300	-5.76457300	-0.30770800
C	-5.65514500	0.50445300	0.31564600
H	-6.20024400	1.11407600	-0.40783600
H	-6.39279700	0.12756100	1.02762900
C	-2.77261600	-1.43012800	-3.40207500
H	-3.38747200	-0.91690500	-4.14827800
H	-2.55915000	-2.42824600	-3.78902900
C	2.29493300	-1.54448100	-3.69874600
H	2.89440100	-0.99817400	-4.43285600
H	2.01155800	-2.49585300	-4.15347500
C	5.62237800	-0.12690500	-0.10695100
H	6.14845600	0.47216300	-0.85275600
H	6.39262300	-0.63865900	0.47594300
C	2.79699000	0.74027300	4.01329500
H	2.71763200	1.53906700	4.74984400
H	3.35290000	-0.07774400	4.47415200
C	-2.29549500	1.26438100	4.02857100
H	-2.92980400	0.59241300	4.60851600
H	-2.09380700	2.11355300	4.68701300

Energy = -3352.623317 a.u.

0 imaginary frequency

Derivatives $V4(H)_2^{4+}/V4(H)_1^{3+}$

TD-DFT calculations on derivative **V4**$(H)_2^{4+}$ predicted the S1 ← S0 transition at **355 nm** (experimental data = **367 nm**).

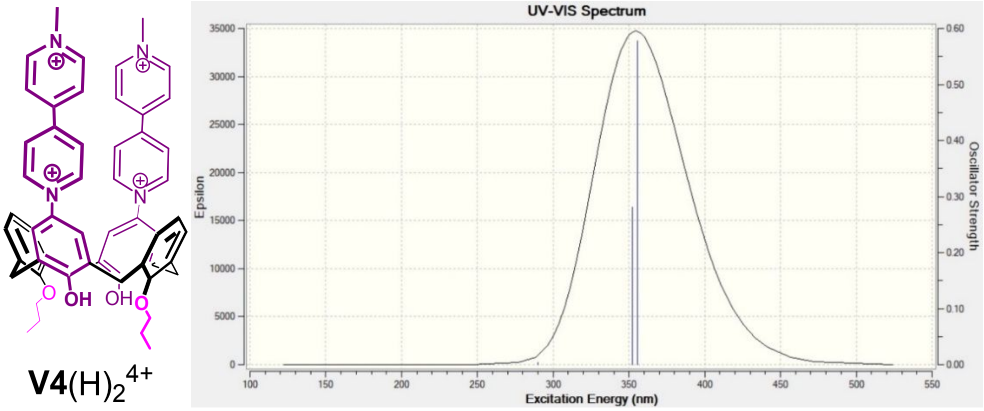

V4$(H)_2^{4+}$

Figure S39. TD-DFT calculated UV-VIS spectrum of derivative **V4**$(H)_2^{4+}$ in acetonitrile.

TD-DFT calculations on derivative **V4**$(H)_1^{1+}$ predicted the S1 ← S0 transition at **594 nm** (experimental data = **686 nm**).

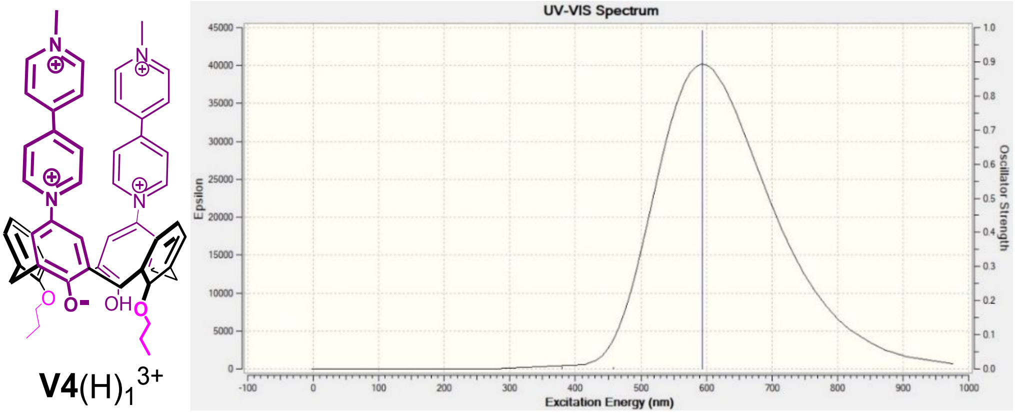

V4$(H)_1^{3+}$

Figure S40. TD-DFT calculated UV-VIS spectrum of derivative **V4**$(H)_1^{3+}$ in acetonitrile.

Atomic coordinate of derivative $V4(H)_2^{4+}$

O	7.1725	7.8151	8.8095
O	3.5723	6.4946	7.7176
O	5.0754	9.0892	8.0778
H	5.8897	8.6058	8.3221
O	5.6736	5.3172	8.5385
H	4.861	5.75	8.2092
N	5.1966	2.7273	13.5234
C	2.5252	6.4697	8.6025
N	2.6814	11.6324	12.4963
C	5.5317	4.7132	9.7568
C	2.2026	8.9882	8.2923
H	2.7058	8.8675	7.3327
H	1.3251	9.5923	8.0602
C	1.7687	7.6286	8.8452
C	3.1291	4.0516	9.1418
H	2.5475	3.1411	9.2982
H	3.4885	3.9557	8.1151
C	4.2188	3.435	11.3405
H	3.2941	2.9188	11.5584
C	2.5221	10.4135	10.3493
H	1.4466	10.3636	10.4382
C	4.5068	9.7426	9.1361
C	2.2187	5.2734	9.2694
C	6.4993	4.0557	11.8758
H	7.3462	4.0952	12.5456
C	5.314	10.3838	10.0934

C	3.1762	6.2611	6.362
H	2.4537	7.0067	6.0229
H	2.6908	5.2876	6.2657
C	3.1086	9.7417	9.2741
C	8.0045	6.8573	10.8659
C	4.7123	11.037	11.1756
H	5.3535	11.5433	11.884
C	0.5996	7.5325	9.6072
H	-0.0335	8.3965	9.7477
C	6.6138	4.7214	10.6522
C	7.4941	9.2337	10.7081
C	3.3145	11.0464	11.3197
C	1.051	5.1981	10.0361
H	0.76	4.2683	10.5042
C	7.9306	5.4324	10.309
H	8.0705	5.4406	9.2282
H	8.7753	4.8491	10.6773
C	7.5581	7.9621	10.1178
C	6.8423	10.4029	9.9718
H	7.138	10.397	8.9206
H	7.2365	11.3493	10.3481
C	5.3048	3.4076	12.2345
C	8.0426	9.4287	11.9795
H	8.0645	10.4146	12.4225
C	0.2324	6.3177	10.1844
H	-0.6855	6.2456	10.7518
C	8.549	7.072	12.1369
H	8.9596	6.2478	12.7017
C	1.352	12.5194	14.8077
C	8.5875	8.3548	12.6818
H	9.0373	8.517	13.6518
C	0.7757	12.6654	13.5378
H	-0.2109	13.0985	13.4268
C	4.4243	6.3062	5.475
H	5.1441	5.564	5.8217
H	4.9037	7.2795	5.5819
C	4.3212	4.0889	10.1071
C	6.2906	2.1393	14.0838
H	7.1988	2.1403	13.4941
C	5.0133	1.5107	16.0542
C	1.4542	12.2137	12.3988
H	1.0479	12.3162	11.3998
C	3.2895	11.5436	13.7103
H	4.2604	11.0668	13.7335
C	4.0071	2.6963	14.1853
H	3.1732	3.1929	13.7086
C	8.2594	7.8793	7.8809
H	9.0543	7.179	8.1443
H	8.7065	8.8751	7.8803
C	7.7222	7.5454	6.4848
H	6.9587	8.2726	6.2051
H	7.2283	6.5731	6.5165
C	3.8959	2.1024	15.4496
H	2.9366	2.1001	15.9521
C	6.2202	1.5242	15.3404
H	7.1121	1.0717	15.7569
C	4.1243	6.0609	3.9884
H	3.6759	5.0802	3.8218
H	5.0392	6.1058	3.3951
H	3.4419	6.8128	3.5897
C	2.6414	11.9716	14.8759
H	3.1407	11.8631	15.8308
C	8.8095	7.517	5.3992
H	9.3069	8.4826	5.3026
H	8.3805	7.2727	4.4264
H	9.5717	6.7674	5.615
C	4.9242	0.903	17.4154
C	4.3892	1.6268	18.4946
C	4.3228	1.0506	19.7695
N	4.7649	-0.2171	19.9626
C	5.295	-0.9491	18.9502
C	5.3811	-0.4017	17.6625
H	4.043	2.6441	18.3556
H	3.9375	1.5703	20.6411
C	4.7037	-0.7899	21.3253
H	5.637	-1.949	19.1972
H	5.7937	-1.0012	16.8584
C	0.4631	11.984	17.0998
C	0.6041	12.8985	16.0428
C	-0.0001	14.1595	16.1807
C	-0.7247	14.4717	17.3401
N	-0.8523	13.5505	18.3279
C	-0.2716	12.3278	18.2413
H	0.9022	10.9961	17.032
H	0.0967	14.9041	15.3977
H	-1.2063	15.4308	17.5048
C	-1.6229	13.9088	19.5391
H	-0.4159	11.6565	19.0809
H	4.7302	-1.8813	21.2774
H	5.5625	-0.4155	21.8885
H	3.7684	-0.4869	21.8039
H	-1.8575	13.0155	20.1233
H	-1.0185	14.5969	20.1357
H	-2.5601	14.3825	19.2351

Energy = -2687.081760 a.u.

0 imaginary frequency

Atomic coordinate of derivative V4(H)$_1^{3+}$

O	-0.02562900	-3.37227900	2.57742300
O	-0.09509500	-3.07191300	-2.49128300
O	1.63495800	-2.74511300	-0.02991900
O	-1.66318200	-2.84368700	-0.26561400
H	-1.19729800	-2.98494100	-1.11731100
N	-6.03588000	0.56163400	-0.12375000

C	0.02145800	-1.71831300	-2.76588200
N	6.04896800	0.53572500	0.07624800
C	-2.68768100	-1.98154200	-0.29853700
C	2.56351400	-1.88231200	-2.52177700
H	2.33269000	-2.94721900	-2.47323800
H	3.25376400	-1.71781700	-3.35529900
C	1.28111600	-1.11297400	-2.78892100
C	-2.51908600	-1.66225100	-2.82306500
H	-3.17504900	-1.28103700	-3.60876800
H	-2.42774100	-2.73748600	-2.99001300
C	-4.29071400	-0.57330500	-1.40736900
H	-4.70782100	-0.15672400	-2.31811300
C	4.34356800	-0.68713200	-1.18153000
H	4.77418300	-0.32264200	-2.11030900
C	2.60967800	-1.96246200	-0.00348100
C	-1.16061400	-0.99230900	-2.94606600
C	-4.37005800	-0.84170700	0.99554100
H	-4.81120200	-0.58238800	1.95238400
C	3.17269400	-1.48874100	1.25001100
C	-0.04988000	-3.93947100	-3.63427000
H	0.89780400	-3.78004400	-4.16132900
H	-0.86474500	-3.68507800	-4.32395700
C	3.22789500	-1.48152300	-1.22648500
C	-1.31993500	-1.35948600	2.49315600
C	4.28036000	-0.68216600	1.25641500
H	4.71825000	-0.39343400	2.20794900
C	1.32989900	0.25783900	-3.04037500
H	2.29699900	0.75165800	-3.05979700
C	-3.25880800	-1.66733900	0.95267200
C	1.11436600	-1.25946600	2.63905200
C	4.89302100	-0.28245600	0.05117700
C	-1.06702200	0.37517700	-3.19307400
H	-1.97477900	0.95371300	-3.33784800
C	-2.59140400	-2.14852600	2.22174200
H	-2.33631000	-3.20431100	2.13259700
H	-3.29150700	-2.03930500	3.05553700
C	-0.07570000	-1.99562000	2.57907000
C	2.47428300	-1.92400000	2.51623800
H	2.33154100	-3.00553900	2.49870900
H	3.09289600	-1.67238200	3.38397000
C	-4.88201600	-0.30873400	-0.18198200
C	1.02829900	0.12826700	2.72712500
H	1.94585200	0.70792900	2.77466400
C	0.17324300	0.99721000	-3.25091300
H	0.23810200	2.06172000	-3.45062500
C	-1.36416900	0.03094600	2.58608200
H	-2.32373100	0.53606900	2.52741100
C	8.31969100	2.16881700	0.10831700
C	-0.20104300	0.77398900	2.73179600
H	-0.25069900	1.85498800	2.81416900
C	8.13652300	1.19260700	-0.87457100
H	8.86122200	1.03977300	-1.66404300
C	-0.16905700	-5.36856600	-3.14874800
H	-1.10725400	-5.47515600	-2.59345600

H	0.64247200	-5.56427200	-2.44068500
C	-3.18075600	-1.40651400	-1.48386900
C	-7.14055700	0.16894300	0.54550100
H	-7.10810100	-0.81554000	0.99069500
C	-8.22728600	2.23620000	0.00297700
C	7.02325500	0.38915200	-0.86219400
H	6.88458900	-0.41067400	-1.57357000
C	6.20584000	1.48861700	1.03476200
H	5.39158600	1.60175700	1.73401800
C	-5.99543500	1.76542500	-0.73345300
H	-5.07071000	2.02147500	-1.23120100
C	-0.08886000	-3.94863400	3.88109600
H	-1.01995700	-3.64486100	4.38008300
H	0.74761700	-3.58046500	4.49139900
C	-0.02538800	-5.45594700	3.74059800
H	0.89777400	-5.71936600	3.21361500
H	-0.85562900	-5.78364200	3.10590200
C	-7.07780400	2.61333200	-0.69228900
H	-7.01133200	3.56259100	-1.20772300
C	-8.24217200	0.98928900	0.62899400
H	-9.10151700	0.64688900	1.19072900
C	-0.12240900	-6.36702800	-4.30108200
H	-0.94047400	-6.19755100	-5.00758400
H	-0.20894300	-7.39104100	-3.93113800
H	0.81821700	-6.29091700	-4.85462500
C	7.32014000	2.28872700	1.07797100
H	7.39079300	3.01326200	1.87923000
C	-0.08282900	-6.16122000	5.09184300
H	0.75315800	-5.86243100	5.73164100
H	-0.03647000	-7.24583700	4.96953200
H	-1.00957700	-5.92559400	5.62397200
C	-9.39718200	3.14058500	0.07972300
C	-9.22403800	4.51550500	0.23625000
C	-10.32601100	5.33834200	0.30902400
N	-11.56900200	4.83246900	0.22446000
C	-11.76448300	3.50936300	0.07086300
C	-10.69872400	2.64209100	-0.00073100
H	-8.24026300	4.95791900	0.32302000
H	-10.24324500	6.40886200	0.43771000
C	-12.74018300	5.72504600	0.33905600
H	-12.79180800	3.17653800	0.00664400
H	-10.89696100	1.58780200	-0.14265000
C	9.44082300	4.35267400	0.58791700
C	9.50916500	3.03756500	0.11756800
C	10.74627900	2.58032800	-0.35103500
C	11.83731600	3.41524500	-0.33949300
N	11.73803500	4.67913300	0.11695300
C	10.56281200	5.14771900	0.57605000
H	8.51204600	4.78182800	0.93975100
H	10.87778300	1.56812000	-0.70965300
H	12.81331200	3.10134300	-0.68551400
C	12.93880100	5.53577100	0.14454600
H	10.55051200	6.17245700	0.92154900
H	-13.49297700	5.41317900	-0.38144600

H	-13.13637900	5.65758500	1.35186300
H	-12.42901700	6.74445300	0.12672500
H	12.62892400	6.57599800	0.20183000
H	13.53992200	5.27667600	1.01596600
H	13.50983600	5.37415900	-0.76724100
H	-8.39989000	-2.63264600	-3.46434800
H	-9.20230800	-3.31200500	-2.04257400
H	-10.43806600	-0.66334200	-1.07478500
H	-9.67957700	-1.84512900	-0.00522600
H	-9.14607500	-0.15508500	0.01537000
H	5.52772300	0.31005400	3.97834200
H	5.22725100	-1.42791500	3.90998100
H	4.65038000	-0.37504600	2.60714100
H	3.74169700	1.94454200	4.38014500
H	2.76633500	1.29415900	3.05170500
H	2.07159100	1.44680100	4.67058900
H	-9.26353200	0.31968500	-2.99855300
H	-7.89261700	0.81466400	-1.99188100
H	-7.62340000	-0.17046300	-3.43592000
H	4.55613000	0.43061400	6.23683600
H	2.90327500	-0.10103400	6.56580500
H	4.16140300	-1.29612100	6.22722800
H	8.13442200	-3.88163700	2.72534300
H	7.10393900	-2.68464300	1.92338100

H	6.41680700	-3.78580500	3.12495500
H	7.89442900	-6.34622700	2.19268400
H	6.16855800	-6.29145000	2.56757100
H	6.70963100	-6.89596400	0.99622300
H	8.91889300	-4.78222900	0.58580400
H	7.81543800	-5.32174100	-0.68110400
H	7.96910800	-3.59481600	-0.31134600
H	-3.95163900	-4.63989500	1.15450200
H	-3.16819500	-4.85422900	-0.43011000
H	-2.20428700	-4.35994400	0.99503300
H	1.46019500	-5.48956700	-2.08137800
H	0.66042200	-5.62380600	-0.49595100
H	-0.04227900	-4.59277700	-1.77265200
H	0.93777300	-4.53052400	1.36704100
H	1.98493800	-5.28436400	2.54096700
H	3.08078500	-4.46072100	-3.15223800
H	4.71374600	-3.83362200	-3.22153200

Energy = -2685.139238 a.u.

0 imaginary frequency

Derivatives V6(H)$_2^{4+}$/V6(H)$_1^{3+}$

TD-DFT calculations on derivative **V6**(H)$_2^{4+}$ predicted the S1 ← S0 transition at **357 nm** (experimental data = **386 nm**).

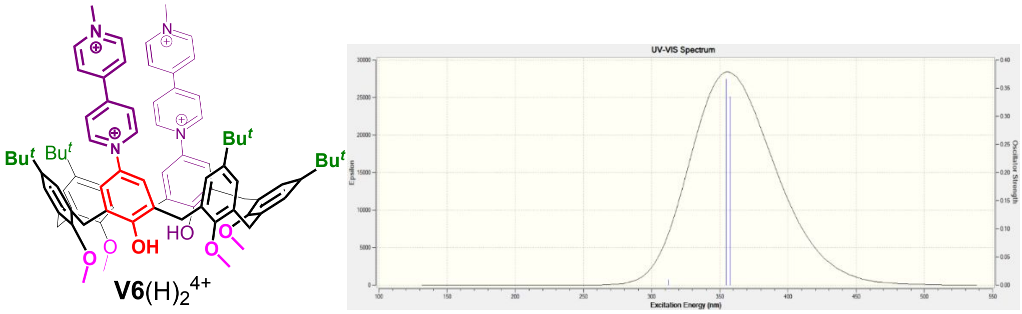

Figure S41. TD-DFT calculated UV-VIS spectrum of derivative **V6(H)$_2^{4+}$** in acetonitrile**.**

TD-DFT calculations on derivative **V6**(H)$_1^{3+}$ predicted the S1 ← S0 transition at **607 nm** (experimental data = **583 nm**).

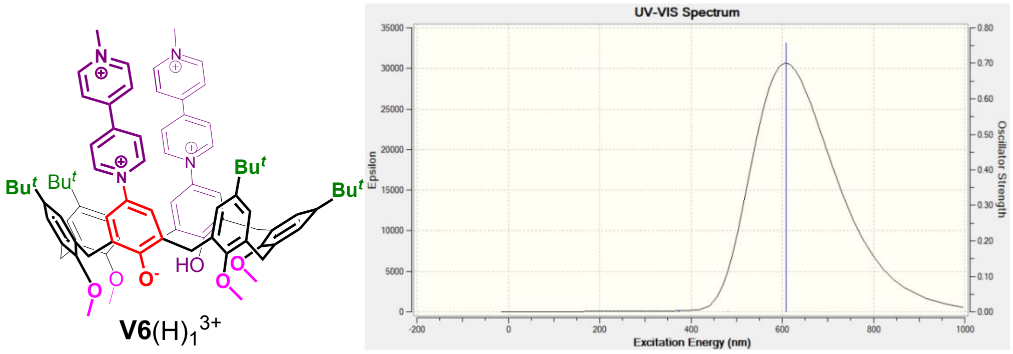

Figure S42. TD-DFT calculated UV-VIS spectrum of derivative **V6**(H)$_1$$^{3+}$ in acetonitrile.

Atomic coordinate of derivative V6(H)$_2$$^{4+}$ in acetonitrile

N	5.31095300	1.48868400	-2.13305500
C	5.70813800	2.29563900	-3.13994900
C	6.73684600	3.78754600	-1.56937900
C	5.60998500	1.79545900	-0.85330200
C	6.32536800	2.93012000	-0.54813800
C	6.41044000	3.45068900	-2.88373700
C	7.50140300	5.01891500	-1.26487600
C	7.21401200	5.77731100	-0.12887400
C	7.94184600	6.91565000	0.13268400
N	8.93067100	7.30691200	-0.69192500
C	9.22917200	6.59462600	-1.79312900
C	8.53029800	5.44932900	-2.10289600
C	9.66672600	8.55408200	-0.40228300
H	9.76150800	8.66493900	0.67523500
H	10.65380300	8.49336700	-0.85328400
H	9.11322600	9.39453100	-0.82034700
O	-3.08539600	-3.44134600	3.14670700
O	-0.51422600	-3.64407400	3.85043000
O	-2.71152800	-3.37118800	-0.07900100
O	0.05758300	-3.09115500	-4.21444600
O	2.59223300	-3.19042800	-3.37662000
O	2.27061800	-3.55447500	-0.25142900
C	0.15576700	-2.43835100	3.64918600
C	3.47115400	0.35527700	-3.28105300
C	1.40575600	-2.45222900	3.03039900
C	-3.49706600	0.18385000	3.34794700
C	2.76727300	-0.79539000	-3.61070200
C	2.03734300	-1.23028500	2.81162700
C	3.18408000	-2.02519600	-3.06477000
C	-0.47199300	-1.25029900	4.01723800
C	-0.49904000	-1.89531200	-3.76517700
C	-5.09801200	-3.17163300	-0.18414300
C	0.19993300	-0.05422600	3.78270000
C	3.33919600	-3.69619500	1.89422300
C	-4.58817700	-2.03066000	2.02658200
C	-0.32487600	0.46233600	-3.42497800
C	2.00651400	-3.77910400	2.59768700
C	-2.97419500	-1.07738900	3.60140800
C	-3.51940300	-2.18645900	2.92698500
C	-4.54018300	0.33379700	2.44610500
C	3.42445600	-3.61045400	0.50220900
C	-5.08722300	-0.75912900	1.78861800
C	0.23051000	-0.71761300	-3.91282600
C	-6.24828700	-3.00725000	-0.95595900
C	4.29086700	-2.09089900	-2.19861100
C	-1.57390700	0.49241900	-2.80414500
C	1.59810300	-0.70945700	-4.57260900
C	1.45992700	-0.01590400	3.18544200
C	-3.86332000	-3.26223300	-0.83047400
C	-1.73771200	-1.92267900	-3.12468400
C	4.66475300	-3.52579800	-0.13491700
C	4.97494300	-0.92542300	-1.88831700
C	-1.86405900	-1.24030900	4.62173000
C	-3.77667700	-3.18316700	-2.22260800
C	4.76301700	-3.41866400	-1.64238500
C	-2.25589900	-0.71687300	-2.65976500
C	2.15601000	1.33284100	2.96485700
C	-4.95081500	-3.01780800	-2.95288600
C	5.82048100	-3.52671800	0.64602600
C	4.56549300	0.28418900	-2.43126400
C	5.77908800	-3.60981500	2.03766800
C	-6.20569900	-2.92870600	-2.34778500
C	4.51964200	-3.69403200	2.63350100
C	7.04030300	-3.61101000	2.91025800
C	-0.28888500	-3.45775300	-5.55743100
C	-0.23748300	-4.28892600	5.10230500
C	3.50840700	1.17494700	2.26050000
C	1.26610800	2.23718000	2.09355200
C	-2.14176300	1.82274600	-2.29308300
C	2.39554800	2.01127400	4.32534700
C	7.09778100	-4.91445100	3.72665900
C	8.32332500	-3.51080600	2.07726500
C	-3.56290800	1.67041400	-1.73876800
C	-1.23759100	2.35872700	-1.16858300
C	6.99643900	-2.41025300	3.87234300
C	-2.18668400	2.84480100	-3.44268400
C	-7.46037500	-2.74888400	-3.21129100

C	-7.33830000	-1.44833900	-4.02538200
C	-7.59377200	-3.94101500	-4.17549400
C	-8.73853100	-2.66844300	-2.36888700
C	1.79145400	-4.81360000	-0.71441800
C	-2.31766500	-4.70509400	0.23010300
C	-2.44700400	-3.25252300	-2.93223200
C	-5.19081400	-3.23041800	1.32532700
H	5.45823600	1.97449100	-4.14130900
H	6.55940500	3.12828400	0.48974100
H	5.24481800	1.11491500	-0.09750200
H	6.69194700	4.07824800	-3.71930100
H	6.41622700	5.50647000	0.55038100
H	7.75424500	7.53939600	0.99638800
H	8.81657200	4.89264800	-2.98550700
H	10.03891500	6.96407700	-2.40763600
N	-5.08013300	1.65360100	2.20011800
C	-6.41268300	1.85633300	2.27575300
C	-6.11372100	4.16827700	1.71500300
C	-4.24826200	2.67217700	1.89486300
C	-4.73838400	3.93659700	1.66191500
C	-6.95223000	3.09697600	2.02581500
C	-6.66796400	5.51466100	1.44318800
C	-6.01411200	6.66267600	1.89168400
C	-6.54980400	7.90187700	1.62153400
N	-7.69579300	8.02034400	0.92693900
C	-8.34704800	6.92979100	0.48335900
C	-7.85658500	5.66713100	0.72830800
C	-8.22328100	9.36287800	0.61015500
H	-9.30808300	9.31284200	0.55976100
H	-7.92428800	10.05291600	1.39512900
H	-7.81610200	9.68357100	-0.34848400
H	-7.01333400	1.00362600	2.55944000
H	-4.03971500	4.72162500	1.40322300
H	-3.19733100	2.42927700	1.82316800
H	-8.02365400	3.22082500	2.11449500
H	-5.10167000	6.60817700	2.47096300
H	-6.08364200	8.82045200	1.95155100
H	-8.40003200	4.81659100	0.33794700
H	-9.25997800	7.10058800	-0.07129700
H	-2.15499400	-3.48002200	3.46744100
H	1.69431000	-3.09635100	-3.76829200
H	3.15604800	1.30940200	-3.68997500
H	-3.11515200	1.04204500	3.89096500
H	3.00700000	-1.24468800	2.33313400
H	-0.28371400	0.87046700	4.08230500
H	0.24088900	1.38142700	-3.54403600
H	1.27426100	-4.28496600	1.96528800
H	2.12860500	-4.41797800	3.47738300
H	-5.88612400	-0.62516300	1.06631400
H	-7.19776300	-2.94378700	-0.43710000
H	1.67379400	-1.52504500	-5.29549000
H	1.69803500	0.21468600	-5.14570600
H	5.84853000	-0.97501900	-1.24634400
H	-1.92734200	-0.41151000	5.33006500
H	-2.03831300	-2.15155400	5.19790700
H	5.80922300	-3.56306900	-1.92756400
H	4.19403100	-4.21404200	-2.12131600
H	-3.22013800	-0.73886800	-2.17074400
H	-4.86935200	-2.95865600	-4.03436300
H	6.77391100	-3.46502100	0.13430800
H	4.43913600	-3.76312700	3.71449100
H	0.04339000	-2.69650000	-6.26836300
H	0.21621900	-4.40088900	-5.76201100
H	-1.36824200	-3.59076100	-5.65318900
H	-0.51851900	-3.64691600	5.94145900
H	-0.83117000	-5.20203800	5.11921300
H	0.82228700	-4.53892100	5.17988200
H	3.96023000	2.16019900	2.11459400
H	4.20750900	0.57177700	2.84644500
H	3.38932600	0.70759600	1.27818000
H	1.75410400	3.20320100	1.93104200
H	1.08169100	1.78037500	1.11689900
H	0.29821800	2.42965100	2.56298400
H	2.88913100	2.97787900	4.18495400
H	1.45857100	2.18945300	4.85892800
H	3.03388900	1.39312500	4.96314700
H	7.99084100	-4.92551100	4.35906600
H	6.22709000	-5.02464400	4.37781300
H	7.13792900	-5.78637700	3.06737000
H	9.19128900	-3.51405800	2.74214600
H	8.42959800	-4.35541000	1.39064100
H	8.35784900	-2.58700100	1.49266300
H	-3.93451500	2.64675300	-1.41562500
H	-4.25437200	1.28601700	-2.49403900
H	-3.59383900	0.99968500	-0.87530800
H	-1.62823000	3.30721900	-0.78603300
H	-1.18891400	1.64984900	-0.33661200
H	-0.21745000	2.53405600	-1.52021700
H	7.88901800	-2.39951000	4.50545300
H	6.96197600	-1.46723000	3.31848200
H	6.12319900	-2.44719100	4.52835600
H	-2.59145800	3.79629100	-3.08453500
H	-1.19420100	3.04185800	-3.85468000
H	-2.82511500	2.48942900	-4.25662500
H	-8.22586500	-1.30805900	-4.65003300
H	-7.24782700	-0.58123100	-3.36433800
H	-6.46626500	-1.46197600	-4.68383300
H	-8.48251300	-3.82235500	-4.80293000
H	-6.72802100	-4.02691900	-4.83681600
H	-7.69073100	-4.88050400	-3.62368300
H	-9.60212300	-2.54017900	-3.02706300
H	-8.89945400	-3.57952900	-1.78570700
H	-8.71847700	-1.81926800	-1.67983400
H	0.95629800	-4.60463500	-1.38264500
H	2.56134000	-5.35757400	-1.26872600
H	1.44795200	-5.43843000	0.11726900
H	-3.12591300	-5.26078700	0.71367900
H	-2.00683200	-5.24638000	-0.67014500

H	-1.47674500	-4.63192800	0.91992600
H	-1.76528500	-3.91841100	-2.39845800
H	-2.60785900	-3.71751900	-3.90897600
H	-4.70530900	-4.12814700	1.70549100
H	-6.24687300	-3.30232100	1.60215300

Energy = -3926.507208 a.u.

0 imaginary frequency

Atomic coordinate of derivative V6(H)$_1^{3+}$ in acetonitrile

N	-3.13779300	2.06233300	2.78381100
C	-2.73034000	2.91953600	3.75947300
C	-3.10730400	4.82626000	2.33994600
C	-3.51950200	2.57825700	1.58401300
C	-3.52781900	3.93094900	1.35161900
C	-2.69271800	4.27632100	3.55631300
C	-3.11145500	6.28138500	2.11387500
C	-2.90294500	6.82090100	0.84084900
C	-2.92295700	8.18398800	0.65728900
N	-3.14265600	9.01829700	1.68963200
C	-3.34374600	8.52996000	2.92991500
C	-3.33177400	7.17759600	3.16748100
C	-3.15264300	10.47884600	1.48282300
H	-3.11446700	10.68458600	0.41653400
H	-4.06911000	10.88952600	1.90276400
H	-2.28414800	10.91440300	1.97564000
O	1.33490500	-3.34388100	-4.09330700
O	-1.31498100	-2.99195200	-4.09142100
O	1.58681700	-3.78985800	-0.87080400
O	-0.63640100	-3.93040100	3.10566800
O	-3.53749900	-3.28025700	4.04770900
O	-3.34182200	-2.87582300	0.21473100
C	-1.77886900	-1.69733000	-3.85745300
C	-2.22887800	0.06945800	3.86849900
C	-2.96191600	-1.51296800	-3.14067700
C	2.23422900	0.18092200	-3.80154500
C	-2.28987300	-1.26484700	4.17499500
C	-3.40276800	-0.20535900	-2.94013400
C	-3.37901300	-2.09041700	3.68049500
C	-1.02302300	-0.62057900	-4.32154400
C	0.38364300	-3.07651500	3.47141300
C	3.94374100	-3.97518700	-1.24528000
C	-1.50626200	0.66474000	-4.09736700
C	-4.81630200	-2.42205200	-1.61947300
C	3.20166900	-2.34410900	-3.06534800
C	1.14721800	-1.16575500	4.69104100
C	-3.72347500	-2.72190700	-2.61753000
C	1.51521900	-0.95032800	-4.16712500
C	1.99166900	-2.21175300	-3.76741800
C	3.40719000	0.04777800	-3.07330300
C	-4.59335300	-2.52044600	-0.24329000
C	3.89934700	-1.19902600	-2.71313200
C	0.12156200	-2.05830300	4.39428600
C	5.22353400	-4.18470400	-0.73335800
C	-4.28959700	-1.45868000	2.73664400
C	2.40729100	-1.24342400	4.09128600
C	-1.23853700	-1.91497500	5.04779700
C	-2.70492000	0.90077700	-3.42322000
C	2.85207800	-4.03367700	-0.37535600
C	1.63617900	-3.20800200	2.86776900
C	-5.60669100	-2.21357600	0.67213100
C	-4.18909200	-0.12504800	2.44395400
C	0.29002800	-0.82124900	-5.05453100
C	3.03323400	-4.29964000	0.98482600
C	-5.38722500	-2.32515800	2.16364700
C	2.62616100	-2.27618500	3.18090300
C	-3.20717500	2.33889900	-3.24410200
C	4.32932400	-4.52474900	1.44677600
C	-6.84682000	-1.80941900	0.17630600
C	-3.17939400	0.66817100	3.02288300
C	-7.11518200	-1.71163300	-1.18911800
C	5.44762800	-4.46800200	0.61384400
C	-6.07680600	-2.02890500	-2.06506400
C	-8.47703100	-1.27461400	-1.74321600
C	-0.77524900	-5.10352100	3.89638400
C	-1.86850100	-3.62279300	-5.25705400
C	-4.57160800	2.39532600	-2.54730800
C	-2.19220300	3.13451600	-2.40427200
C	3.48697400	-0.21534800	4.45453600
C	-3.35418000	3.00594600	-4.62386400
C	-9.05648300	-2.39649200	-2.62324800
C	-9.48668200	-0.96909200	-0.63085900
C	4.79463600	-0.44815600	3.68897800
C	2.98272700	1.19992900	4.12000300
C	-8.29957200	-0.00369800	-2.59348000
C	3.79099100	-0.30188300	5.96089400
C	6.84585300	-4.72546600	1.18946100
C	7.13792700	-3.70862700	2.30730100
C	6.90370000	-6.14913400	1.77155200
C	7.94380500	-4.59650900	0.12734800
C	-3.16739900	-4.26231200	0.49270600
C	0.88742000	-4.94541600	-1.32538900
C	1.85917000	-4.38330300	1.92878900
C	3.74730300	-3.71351400	-2.72248800
H	-2.47935200	2.47486900	4.71012900
H	-3.87041100	4.27425900	0.38379800
H	-3.78926400	1.86166100	0.82355400
H	-2.34460800	4.89734600	4.37178100
H	-2.70235400	6.19255800	-0.01653200
H	-2.76128900	8.64021600	-0.30964300
H	-3.52398500	6.83279100	4.17462500
H	-3.52106700	9.25670400	3.71175200
N	4.15358700	1.23382800	-2.71066200

C	5.46834500	1.31167200	-3.00671500
C	5.58225500	3.49287800	-2.01811100
C	3.53068600	2.25241200	-2.08128300
C	4.21900500	3.39292500	-1.73669600
C	6.20373600	2.42189800	-2.66163000
C	6.34923800	4.70337700	-1.64411400
C	5.77358700	5.97036700	-1.72881800
C	6.50964700	7.08028500	-1.37548000
N	7.77714700	6.95683000	-0.94537700
C	8.35688900	5.74499800	-0.85227300
C	7.66811100	4.60514800	-1.19555900
C	8.56372600	8.15438500	-0.58616200
H	9.33865600	8.30455500	-1.33674800
H	7.90227600	9.01574600	-0.55784000
H	9.01118400	8.00117700	0.39402600
H	5.89170900	0.47230500	-3.54048400
H	3.68754500	4.17885200	-1.21622100
H	2.48424300	2.10736200	-1.85188900
H	7.25037700	2.45107000	-2.93498800
H	4.76263900	6.11354700	-2.08730900
H	6.10798200	8.08272200	-1.43080400
H	8.16372400	3.64916500	-1.08782600
H	9.37750900	5.72260400	-0.49418200
H	0.36098300	-3.19373100	-4.11316400
H	-1.39583300	0.65750200	4.24472600
H	1.90224300	1.16051300	-4.12908200
H	-4.32506800	-0.06498100	-2.39384200
H	-0.92961100	1.50073200	-4.48111600
H	0.94556300	-0.38447800	5.41839100
H	-2.99830700	-3.41539700	-2.18925200
H	-4.17796100	-3.24468400	-3.46595300
H	4.81252900	-1.28571200	-2.13292400
H	6.05664200	-4.13648900	-1.42476700

H	-1.63209300	-2.89130300	5.33165800
H	-1.10994100	-1.33083300	5.96496900
H	-4.93467100	0.32730100	1.79614300
H	0.44208800	0.03567600	-5.71438600
H	0.23372500	-1.70006800	-5.70117200
H	-6.33700700	-2.10170500	2.66372400
H	-5.13921000	-3.35083800	2.44630600
H	3.58805100	-2.37353600	2.69595100
H	4.45611400	-4.75733900	2.50011400
H	-7.62008600	-1.57326700	0.89809000
H	-6.23828700	-1.96764600	-3.13757700
H	-0.78416500	-4.86589500	4.96416000
H	-1.73164200	-5.54921500	3.62619900
H	0.03353100	-5.81573200	3.69908300
H	-1.65868900	-3.03159900	-6.15246300
H	-1.39259900	-4.59961400	-5.33726200
H	-2.94691600	-3.75040400	-5.15185400
H	-4.88574700	3.43747500	-2.44301100
H	-5.34172900	1.87198100	-3.12116000
H	-4.54303700	1.95489100	-1.54666900
H	-2.53656300	4.16541900	-2.27230700
H	-2.06276300	2.68745800	-1.41425000
H	-1.21106100	3.17054100	-2.88480100
H	-3.71576500	4.03241000	-4.50937300
H	-2.40366600	3.04670500	-5.16105600
H	-4.07008900	2.46154400	-5.24619400
H	-10.02627400	-2.09525400	-3.03141200

Energy = -3926.018123 a.u.

0 imaginary frequency

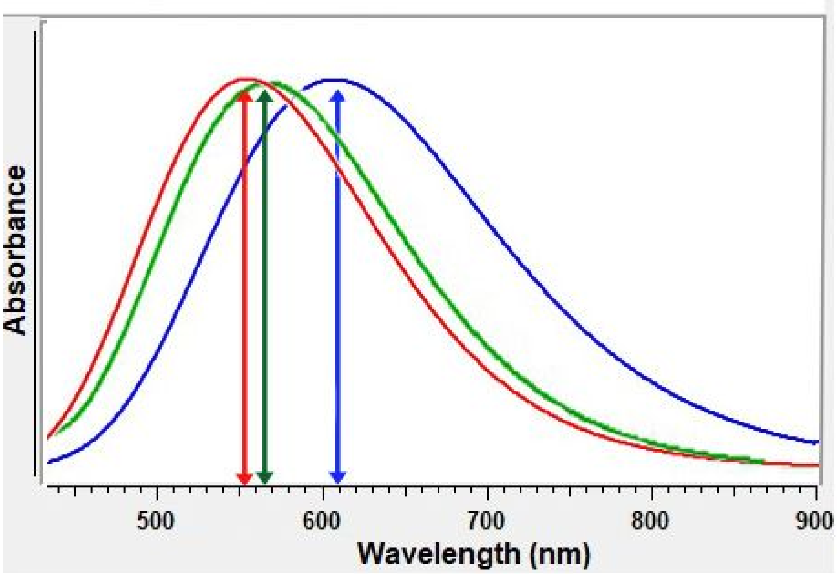

Figure S43. Simulated absorption bands of **V6**(H)$_1^{3+}$: (blue curve) in presence of one methanol molecule; (green curve) in presence of one water molecule (red curve).

Atomic coordinate of derivative V6(H)$_1^{3+}$ with 1 methanol molecule

N	2.86689600	2.24137500	-2.75359900
C	2.53303500	2.99939900	-3.82770600
C	2.64059200	5.00675000	-2.51313600
C	3.08217900	2.84561300	-1.55939800
C	2.99123800	4.20990100	-1.42125100
C	2.39978400	4.36363500	-3.72850000
C	2.53759400	6.47394500	-2.38847100
C	2.06014400	7.06817400	-1.21860900
C	1.97777300	8.43954100	-1.12963300
N	2.35356300	9.22139300	-2.15729500
C	2.81862300	8.67660800	-3.29855300
C	2.91885400	7.31350700	-3.44007600
C	2.27868500	10.69102100	-2.04615300
H	1.76638000	10.95012700	-1.12380700
H	3.28939700	11.09714500	-2.03581000
H	1.72393700	11.08183300	-2.89728800
O	-1.22039900	-2.94692600	4.39052100
O	1.37221500	-2.30794500	4.44935900
O	-1.36630100	-3.69008100	1.17407900
O	0.90953700	-3.94710800	-2.76109900
O	3.70818600	-3.12056500	-3.70224000
O	3.40548000	-2.37524800	0.32080800
C	1.71735300	-0.99295300	4.13896700
C	2.14244400	0.12036100	-3.73330100
C	2.91604300	-0.73335900	3.47344800
C	-2.47282600	0.42585100	3.77836100
C	2.31874300	-1.22567300	-3.95262300
C	3.23127200	0.59649700	3.19563600
C	3.44018800	-1.92273400	-3.37176800
C	0.82979500	0.02763700	4.48197800
C	-0.17983000	-3.21800900	-3.18769800
C	-3.69516500	-4.08590800	1.55958300
C	1.18993400	1.33744500	4.18363200
C	4.87259000	-1.58579700	2.04206100
C	-3.14892200	-2.24100800	3.24061100
C	-1.10288300	-1.47036100	-4.53699300
C	3.84109300	-1.88256900	3.10524700
C	-1.65450100	-0.58866100	4.25974200
C	-1.98220500	-1.92237200	3.95618700
C	-3.59996700	0.10796000	3.03515800
C	4.63202000	-1.86867100	0.69379900
C	-3.94924600	-1.20977200	2.77423200
C	-0.00209900	-2.23956800	-4.17213900
C	-4.94207900	-4.46656100	1.06534700
C	4.24301600	-1.19139900	-2.42072600
C	-2.36025700	-1.63176300	-3.94827700
C	1.35004800	-2.00413000	-4.81650000
C	2.39525200	1.65266400	3.55447100
C	-2.59451400	-4.10461200	0.69916600
C	-1.42357100	-3.42646500	-2.58676800
C	5.59789900	-1.58911700	-0.27975400
C	4.03509000	0.15288700	-2.21767200
C	-0.48901600	-0.25819700	5.17491000
C	-2.73393500	-4.50133600	-0.63382800
C	5.37552900	-1.92569700	-1.73673800
C	-2.49339900	-2.61817500	-2.97240300
C	2.75768900	3.12061700	3.29765700
C	-3.99626400	-4.89332100	-1.07735000
C	6.80060700	-1.00882000	0.12623700
C	3.00837400	0.82802200	-2.89137700
C	7.08066300	-0.71312700	1.45993200
C	-5.12291400	-4.88151600	-0.25394400
C	6.09569000	-1.02191900	2.39885000
C	8.40071400	-0.07882200	1.91544400
C	1.14846100	-5.16461500	-3.45943600
C	1.94631900	-2.80847900	5.66700600
C	4.13017800	3.27164100	2.63169100
C	1.69515000	3.76006900	2.38641900
C	-3.52823000	-0.74098000	-4.39191600
C	2.79550100	3.87879800	4.63709400
C	9.11090800	-1.02351600	2.90134500
C	9.35066600	0.19190100	0.74307900
C	-4.82212000	-1.05533200	-3.63204600
C	-3.17331800	0.73556100	-4.14099400
C	8.10950600	1.26030600	2.61661000
C	-3.79108000	-0.95069300	-5.89390100
C	-6.48218400	-5.32478300	-0.80931800
C	-6.86652900	-4.43010800	-2.00128200
C	-6.38901400	-6.78673400	-1.28154900
C	-7.59678800	-5.22989700	0.23890900
C	3.31533700	-3.79799400	0.25151800
C	-0.55765700	-4.72222300	1.73246400
C	-1.54874000	-4.54719500	-1.56653600
C	-3.54064600	-3.68456900	3.01001100
H	2.41313900	2.47402400	-4.76332700
H	2.11569200	4.91441700	-4.61614700
H	1.72590700	6.47600400	-0.37679700
H	1.60813700	8.94137400	-0.24577500
H	3.31797900	6.91812600	-4.36474600
H	3.10921900	9.36653500	-4.07988400
N	-4.45191700	1.17216200	2.54823200
C	-5.77639600	1.13774800	2.80618000
C	-6.08396000	3.18887800	1.60274700
C	-3.91771400	2.18673800	1.83657800
C	-4.70892800	3.20881200	1.36447000
C	-6.61105200	2.12490100	2.33600200
C	-6.96397200	4.26438300	1.08999400
C	-6.53255400	5.59108100	1.05523800
C	-7.37609400	6.57022400	0.58072800
N	-8.61163600	6.26398000	0.14578900
C	-9.05155900	4.99288200	0.16415700
C	-8.25031800	3.97603500	0.63182700
C	-9.48103700	7.32764200	-0.39594400
H	-10.51835100	7.06861500	-0.19855400
H	-9.23711000	8.26596400	0.09588800

H	-9.31100800	7.40938800	-1.46922900
H	-6.12737800	0.31304400	3.41047700
H	-7.66383500	2.06912900	2.57940200
H	-5.55509700	5.88239600	1.41704600
H	-7.09166400	7.61305700	0.54098800
H	-8.63658200	2.96533200	0.61601500
H	-10.05325000	4.82069100	-0.20566900
H	-0.27016100	-2.68768700	4.42999200
H	1.29133900	0.62443300	-4.18390400
H	-2.25667500	1.45949400	4.02843800
H	4.16618700	0.79468600	2.68949600
H	0.50876700	2.13138900	4.47344200
H	-0.96354400	-0.72083500	-5.31085500
H	3.22109300	-2.72947100	2.80952800
H	4.37668800	-2.19901800	4.00727900
H	-4.83009000	-1.44065200	2.18378100
H	-5.78251000	-4.44672400	1.74932600
H	1.83173900	-2.95275200	-5.05438500
H	1.18555300	-1.47009400	-5.75789700
H	4.70993200	0.69598200	-1.56220300
H	-0.76129900	0.62529000	5.75655600
H	-0.36944000	-1.07284700	5.89317500
H	6.31172900	-1.73547300	-2.27553800
H	5.18979900	-2.99470300	-1.84972000
H	-3.44904400	-2.77586700	-2.49082900
H	-4.08849900	-5.22381900	-2.10789600
H	7.53595900	-0.79615000	-0.64108600
H	6.27205800	-0.81902700	3.45138900
H	1.16452400	-5.00288100	-4.54180700
H	2.12627100	-5.52195200	-3.13744500
H	0.38178000	-5.91226700	-3.22738400
H	1.68184100	-2.16583100	6.51094700
H	1.53419900	-3.80590100	5.81682300
H	3.03221200	-2.87239900	5.58444100
H	4.33951200	4.33115700	2.46061700
H	4.93151500	2.87294200	3.26023000
H	4.17826300	2.76137100	1.66556000
H	1.94537000	4.80795500	2.19109800
H	1.63140200	3.23629200	1.42813000
H	0.70285300	3.73644200	2.84381800
H	3.05472600	4.92863600	4.46899500
H	1.82985600	3.85191700	5.14773600
H	3.54384000	3.44781800	5.30820200
H	10.05228000	-0.57970500	3.23999800
H	8.49944200	-1.22228200	3.78507000
H	9.33896100	-1.98253000	2.42716300
H	10.27246400	0.64586500	1.11705800
H	9.62379000	-0.72888300	0.22003500
H	8.91295500	0.88178600	0.01593300
H	-5.62087900	-0.39490700	-3.98105900
H	-5.14989800	-2.08588400	-3.79479600
H	-4.70877200	-0.89932700	-2.55532400
H	-3.99612800	1.38336700	-4.45971500
H	-2.99042900	0.91691300	-3.07766800
H	-2.27918800	1.03747900	-4.69206900
H	9.04278300	1.72511500	2.94948300
H	7.60751300	1.95569300	1.93711100
H	7.47167600	1.12843200	3.49438000
H	-4.61954400	-0.31748600	-6.22683300
H	-2.91561600	-0.69930800	-6.49777600
H	-4.05510300	-1.99172300	-6.10165400
H	-7.83537600	-4.73621900	-2.40786700
H	-6.94260600	-3.38248200	-1.69488900
H	-6.13250100	-4.49226800	-2.80871200
H	-7.35074600	-7.11565700	-1.68728900
H	-5.63637200	-6.91304600	-2.06376300
H	-6.12640400	-7.44888700	-0.45140700
H	-8.54325900	-5.55118700	-0.20432400
H	-7.40040000	-5.87334100	1.10114100
H	-7.73029800	-4.20567500	0.59885600
H	4.05103600	-4.22595900	-0.43402100
H	3.43723600	-4.25096100	1.24233000
H	2.31685100	-4.02162900	-0.12760300
H	-1.08528800	-5.26230000	2.52345000
H	-0.24099500	-5.43738900	0.96560400
H	0.31913600	-4.23583700	2.16069100
H	-0.62227700	-4.57096700	-0.99422400
H	-1.58909800	-5.49781900	-2.11147100
H	-2.80192300	-4.31783900	3.50034600
H	-4.49286000	-3.86709600	3.51769400
H	-4.24424600	3.99177400	0.77977900
H	-2.85509700	2.13154700	1.64597100
H	3.19933700	4.64131700	-0.45057100
H	3.30616200	2.19174900	-0.72990300
C	5.57815000	-5.76633400	-3.30046800
O	4.51910900	-5.30668900	-2.49231800
H	5.96391100	-6.69618100	-2.87252800
H	6.40962900	-5.04936900	-3.34878200
H	5.25864800	-5.97883900	-4.33022800
H	4.19384800	-4.45933300	-2.89314000

Energy = -4041.712504 a.u.

0 imaginary frequency

Atomic coordinate of derivative V6(H)$_1^{3+}$ with 1 water molecule

N	2.74721800	1.96080900	-2.84975400
C	2.40207300	2.75608400	-3.89224000
C	2.59527200	4.72138300	-2.52576800
C	3.01351200	2.52432800	-1.64661300
C	2.96112000	3.88608800	-1.46824800
C	2.30415900	4.11966700	-3.75104500
C	2.52729100	6.18651200	-2.35564800
C	2.10189300	6.75513700	-1.15358400
C	2.05633900	8.12470900	-1.02066400

N	2.41881100	8.92873100	-2.03572700
C	2.83023300	8.40875100	-3.20842300
C	2.89237900	7.04867900	-3.39438700
C	2.35377600	10.39572400	-1.88827500
H	2.16538600	10.63827000	-0.84599900
H	3.30491300	10.82379500	-2.19957600
H	1.54624500	10.77832200	-2.51120600
O	-0.98687600	-2.92948600	4.38884800
O	1.63821600	-2.39448300	4.33598100
O	-1.39562400	-3.70672700	1.13938400
O	0.57729400	-4.11403400	-2.89250900
O	3.37361800	-3.41290400	-3.92714600
O	3.33497700	-2.50934900	0.29762800
C	2.03116400	-1.09145000	4.03157400
C	1.92725800	-0.11323400	-3.85496600
C	3.21846100	-0.86956000	3.33318100
C	-2.09296400	0.50158700	3.82024300
C	2.04967500	-1.45942800	-4.10816000
C	3.57769800	0.45129200	3.06196700
C	3.16412500	-2.20853800	-3.57953200
C	1.20034600	-0.03954900	4.42114100
C	-0.49455800	-3.32978300	-3.26000800
C	-3.70690600	-3.95123200	1.70709300
C	1.60572700	1.25898500	4.13372300
C	5.03636300	-1.80407200	1.77255700
C	-2.92843100	-2.12711600	3.32311500
C	-1.39517500	-1.53375100	-4.56019900
C	4.10288000	-2.03971900	2.93595700
C	-1.31272600	-0.55312400	4.27730200
C	-1.71750300	-1.86822500	3.98748000
C	-3.25909300	0.24243500	3.11513300
C	4.63581900	-2.10508600	0.46396200
C	-3.68786200	-1.05634500	2.87732600
C	-0.31746600	-2.35726400	-4.25031900
C	-5.01011600	-4.25529400	1.31578500
C	4.02714100	-1.52053200	-2.64810400
C	-2.62814500	-1.63483300	-3.90870000
C	1.01752500	-2.18234900	-4.94672200
C	2.79972600	1.53513900	3.46511000
C	-2.67974600	-4.05030900	0.76549600
C	-1.71330800	-3.47550700	-2.59407600
C	5.52599700	-1.94635600	-0.60695200
C	3.87207600	-0.17415100	-2.41002800
C	-0.10416800	-0.28161700	5.15530500
C	-2.94571600	-4.45518800	-0.54548500
C	5.16767100	-2.30225200	-2.03323100
C	-2.75993500	-2.61415900	-2.92565900
C	3.22103800	2.99161400	3.23494800
C	-4.26107200	-4.76905300	-0.88496400
C	6.80477900	-1.45377800	-0.33170100
C	2.84414000	0.54727100	-3.02952400
C	7.23366200	-1.12510000	0.95230100
C	-5.31827800	-4.67211100	0.02091400
C	6.32276800	-1.32108200	1.99154700

C	8.63486400	-0.57919700	1.25274300
C	0.72198500	-5.33526100	-3.61035300
C	2.24746900	-2.94485200	5.51418900
C	4.52419100	3.09997000	2.43494700
C	2.11454600	3.74162900	2.47270100
C	-3.77119200	-0.68544200	-4.29170700
C	3.43941600	3.67148300	4.59925100
C	9.36732400	-1.54043700	2.20580300
C	9.48243000	-0.42368600	-0.01512400
C	-5.04111400	-0.94177600	-3.47193300
C	-3.33228300	0.77026600	-4.05177700
C	8.51287200	0.80213600	1.92108700
C	-4.11589200	-0.87191700	-5.78016100
C	-6.74231700	-5.02989000	-0.42257200
C	-7.15492700	-4.13003200	-1.60115300
C	-6.78341700	-6.50211100	-0.86977300
C	-7.76550800	-4.84203300	0.70347600
C	3.08902200	-3.80782300	-0.24547400
C	-0.61471300	-4.77612200	1.66546200
C	-1.84175700	-4.58815900	-1.56542400
C	-3.41314300	-3.54850700	3.13489300
H	2.24150400	2.26014400	-4.83778800
H	2.00690800	4.70221900	-4.61363800
H	1.78221700	6.14612600	-0.31821200
H	1.72904800	8.60766800	-0.11016900
H	3.25175500	6.67323400	-4.34336300
H	3.11028900	9.11584300	-3.97788800
N	-4.06437700	1.34932600	2.64543100
C	-5.38962500	1.36880100	2.90192500
C	-5.60662100	3.44839100	1.72882500
C	-3.48600700	2.35163500	1.95060000
C	-4.23123600	3.41491700	1.49560300
C	-6.18082200	2.39667600	2.44402000
C	-6.43778200	4.56674300	1.22735000
C	-5.96215800	5.87915700	1.24309600
C	-6.76101800	6.89783800	0.77702100
N	-7.99398200	6.64457400	0.30016000
C	-8.47391300	5.38916700	0.26731500
C	-7.71847700	4.33334500	0.72714500
C	-8.80729100	7.76076600	-0.22278700
H	-9.85000400	7.45514200	-0.24892400
H	-8.69244300	8.61812100	0.43659500
H	-8.46263100	8.00705500	-1.22677800
H	-4.98516100	6.12628500	1.63743100
H	-6.44415400	7.93211400	0.77513600
H	-8.13429900	3.33596200	0.67061800
H	-9.46819800	5.25880200	-0.13720800
H	-0.02592500	-2.71076100	4.39074000
H	1.07738700	0.42766200	-4.26317600
H	-1.81309400	1.52222800	4.05962700
H	4.50291500	0.61812800	2.52651600
H	0.97192800	2.07539000	4.46553000
H	-1.25755900	-0.78797600	-5.33797400
H	3.46017700	-2.89508700	2.72185100

H	4.71479400	-2.31624800	3.80218600
H	-4.60088300	-1.24397700	2.32140200
H	-5.79223300	-4.17270700	2.06141400
H	1.44350900	-3.15075800	-5.20897600
H	0.84510200	-1.63133300	-5.87693600
H	4.58952900	0.33271100	-1.77106400
H	-0.32115400	0.60493800	5.75527600
H	0.00739000	-1.10917700	5.85992400
H	6.06705000	-2.16910100	-2.64689000
H	4.92682500	-3.36336400	-2.11525000
H	-3.69580200	-2.72297800	-2.39422700
H	-4.45326200	-5.10599700	-1.89947000
H	7.47803700	-1.33118200	-1.17219600
H	6.61622200	-1.10501900	3.01494900
H	0.68634800	-5.16480400	-4.69115000
H	1.69871200	-5.73745400	-3.34225000
H	-0.06644000	-6.04767300	-3.34190400
H	2.05946000	-2.30494200	6.38054100
H	1.79330600	-3.92296800	5.67023000
H	3.32305300	-3.06197600	5.37385100
H	4.77292700	4.15338500	2.28048100
H	5.36402300	2.63703000	2.96057200
H	4.44311100	2.62743300	1.45171500
H	2.41618700	4.77901200	2.29655600
H	1.91199100	3.27173300	1.50594600
H	1.17621300	3.76319800	3.03200900
H	3.73731200	4.71521700	4.45907000
H	2.52987200	3.66119000	5.20505100
H	4.22731700	3.16624900	5.16513700
H	10.36690900	-1.15838200	2.43560300
H	8.83162000	-1.66140100	3.15067600
H	9.47807500	-2.52987500	1.75255400
H	10.46675800	-0.02770100	0.24946800
H	9.63611400	-1.38075500	-0.52160300
H	9.02605000	0.27062000	-0.72641500
H	-5.82296300	-0.24221800	-3.78059600
H	-5.42499800	-1.95484000	-3.62196800
H	-4.87015800	-0.79669300	-2.40134900
H	-4.13693100	1.45976700	-4.32614000
H	-3.08848200	0.93528600	-2.99807300
H	-2.45267500	1.03173100	-4.64531100
H	9.50573500	1.20511800	2.14423700
H	7.99933200	1.50984200	1.26337600
H	7.95576300	0.75053500	2.86008300
H	-4.92708400	-0.19643900	-6.06963400
H	-3.25927300	-0.65958300	-6.42467500
H	-4.44091500	-1.89727200	-5.97956600
H	-8.17057500	-4.37503000	-1.92694500
H	-7.13631200	-3.07482300	-1.31261200
H	-6.49089500	-4.25595200	-2.46011400
H	-7.79321000	-6.77090900	-1.19508300
H	-6.10295700	-6.69185200	-1.70366400
H	-6.50309700	-7.16752600	-0.04802700
H	-8.76248800	-5.10266000	0.33795900
H	-7.54944800	-5.48513400	1.56126700
H	-7.80066800	-3.80586400	1.05204000
H	2.78713800	-3.74512400	-1.29214000
H	3.96896100	-4.44912600	-0.16303700
H	2.27333600	-4.24918600	0.33304000
H	-1.12996800	-5.28638700	2.48386100
H	-0.37184400	-5.51012500	0.88940600
H	0.30437500	-4.33307600	2.05031600
H	-0.87810800	-4.68113000	-1.06605600
H	-1.99408000	-5.53154400	-2.10325100
H	-2.67237100	-4.21784000	3.57108300
H	-4.32893600	-3.67657900	3.72048500
H	-7.23597800	2.38170100	2.68380800
H	-5.77719100	0.55237500	3.49472700
O	4.04000000	-5.69266300	-2.69072300
H	4.53008300	-6.16590500	-3.37348900
H	3.80736200	-4.83180400	-3.11848500
H	3.24642400	1.84101800	-0.84319800
H	3.21115400	4.28595600	-0.49412500
H	-3.73206700	4.18869100	0.92716800
H	-2.42673300	2.25460300	1.75888900

Energy = -4002.442376 a.u.

0 imaginary frequency

^{1}H NMR of derivative P6$(H)_2^{2+}(Cl^-)_2$ in different solvents

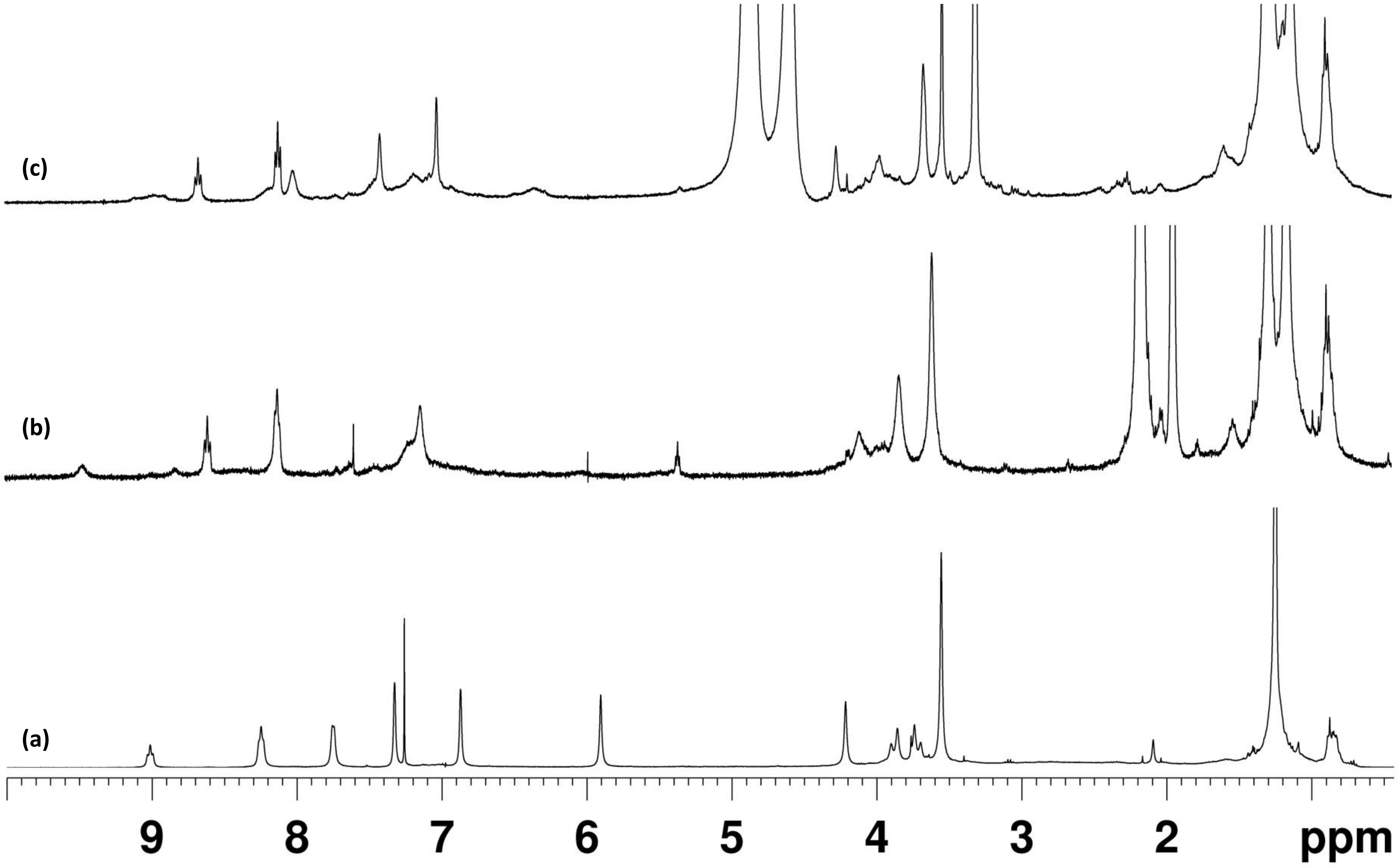

Figure S44. ^{1}H NMR spectra (400 MHz, 298 K) of derivative **P6**$(H)_2^{2+}\cdot(Cl^-)_2$ in: (a) $CDCl_3$; (b) in CD_3CN; (c) in CD_3OD.

^{1}H NMR spectra of derivative $P6(H)_2^{2+}(Cl^-)_2$ and $P6(H)_1^+$ in acetonitrile

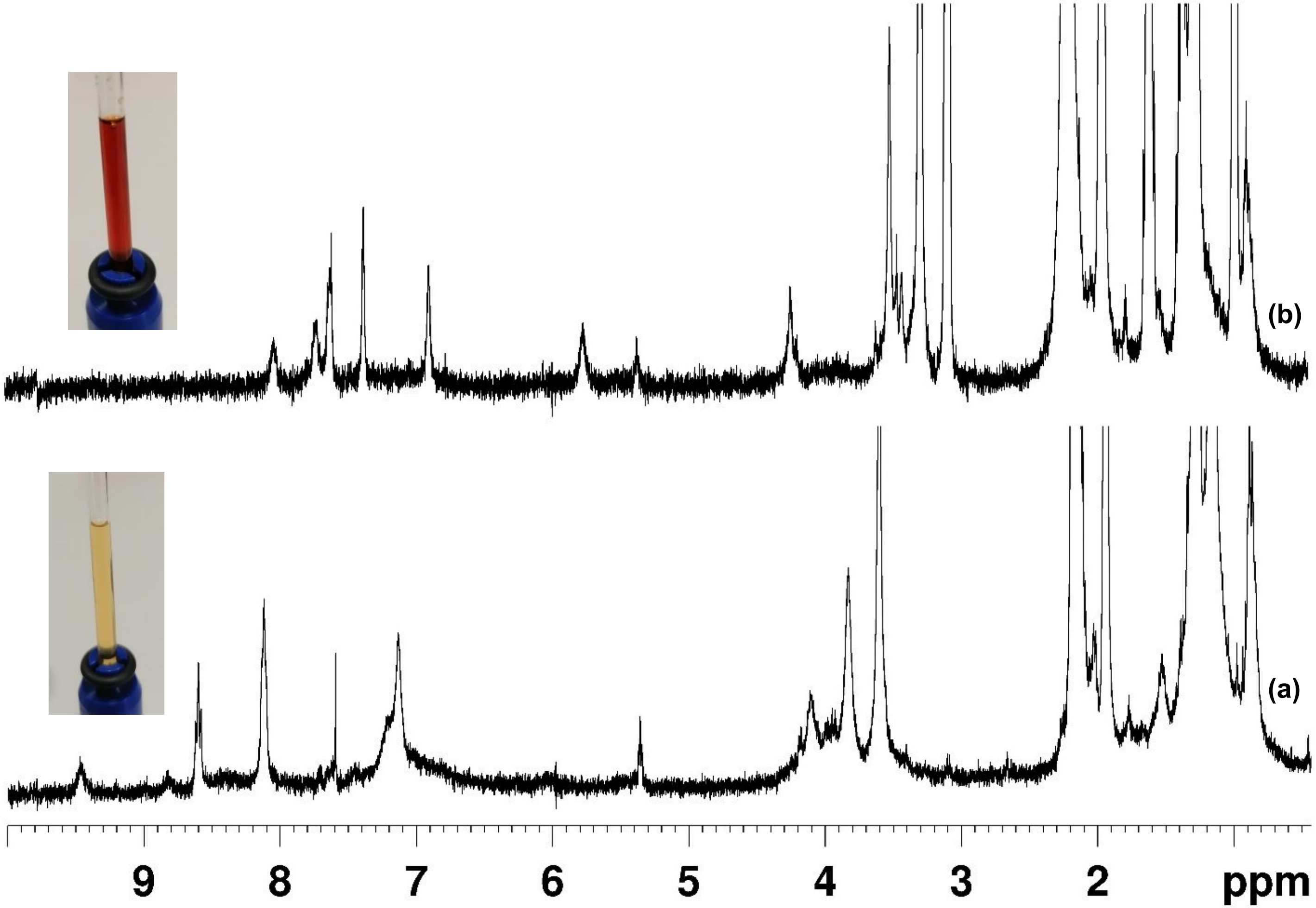

Figure S45. ^{1}H NMR spectra (400 MHz, CD_3CN, 298 K) of: (a) derivative **P6**$(H)_2^{2+}\cdot(Cl^-)_2$ and (b) derivative **P6**$(H)_1^+$, obtained by addition of 1 equivalent of TBAOH to the solution in (a).

2D COSY of derivative $P6(H)_2^{2+}(Cl^-)_2$ in acetonitrile

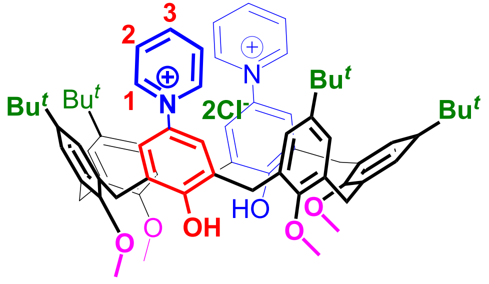

P6$(H)_2^{2+}\cdot(Cl^-)_2$

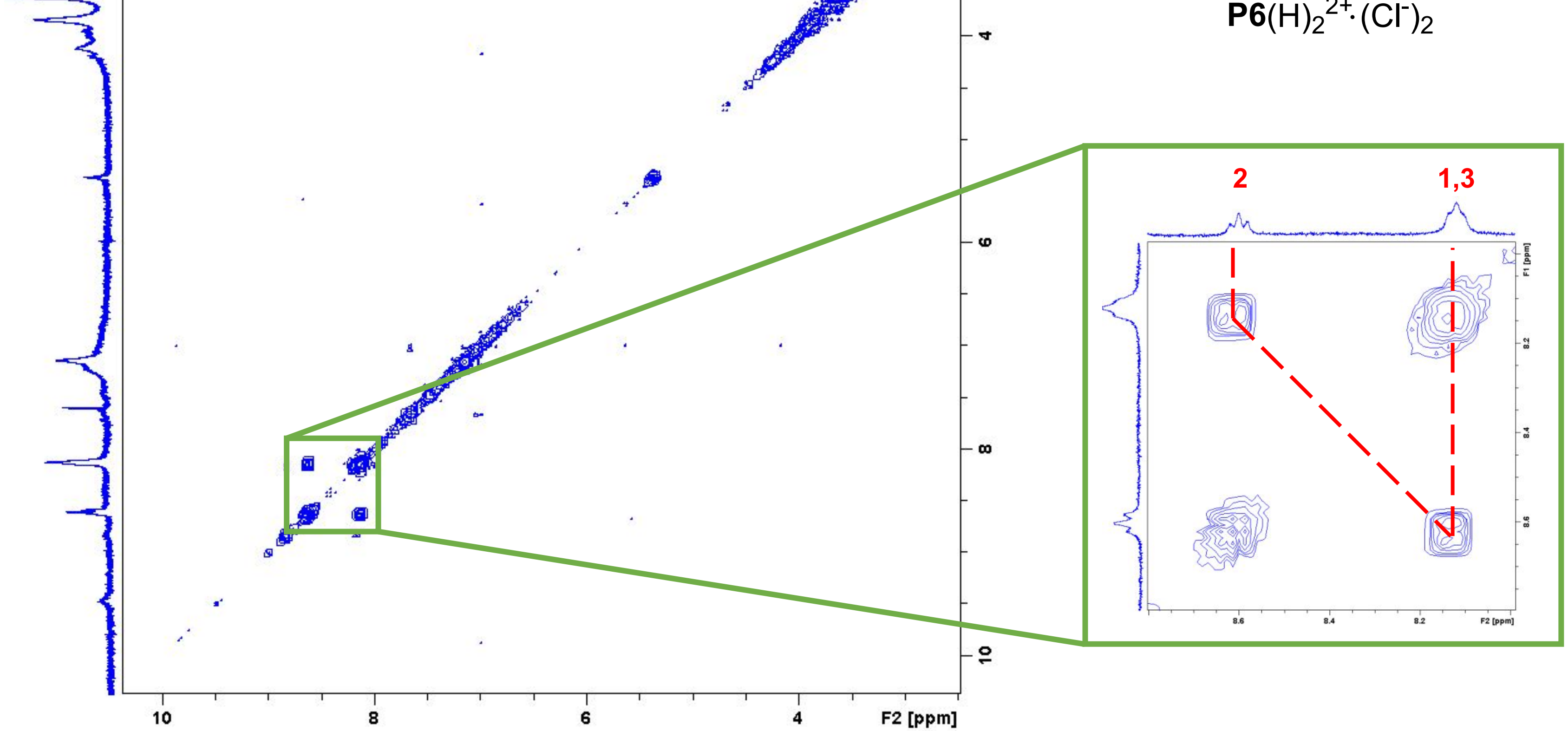

Figure S46. 2D COSY spectrum of derivative **P6**$(H)_2^{2+}\cdot(Cl^-)_2$ (400 MHz, CD_3CN, 298 K).

2D COSY of derivative P6(H)$_1^+$ in acetonitrile

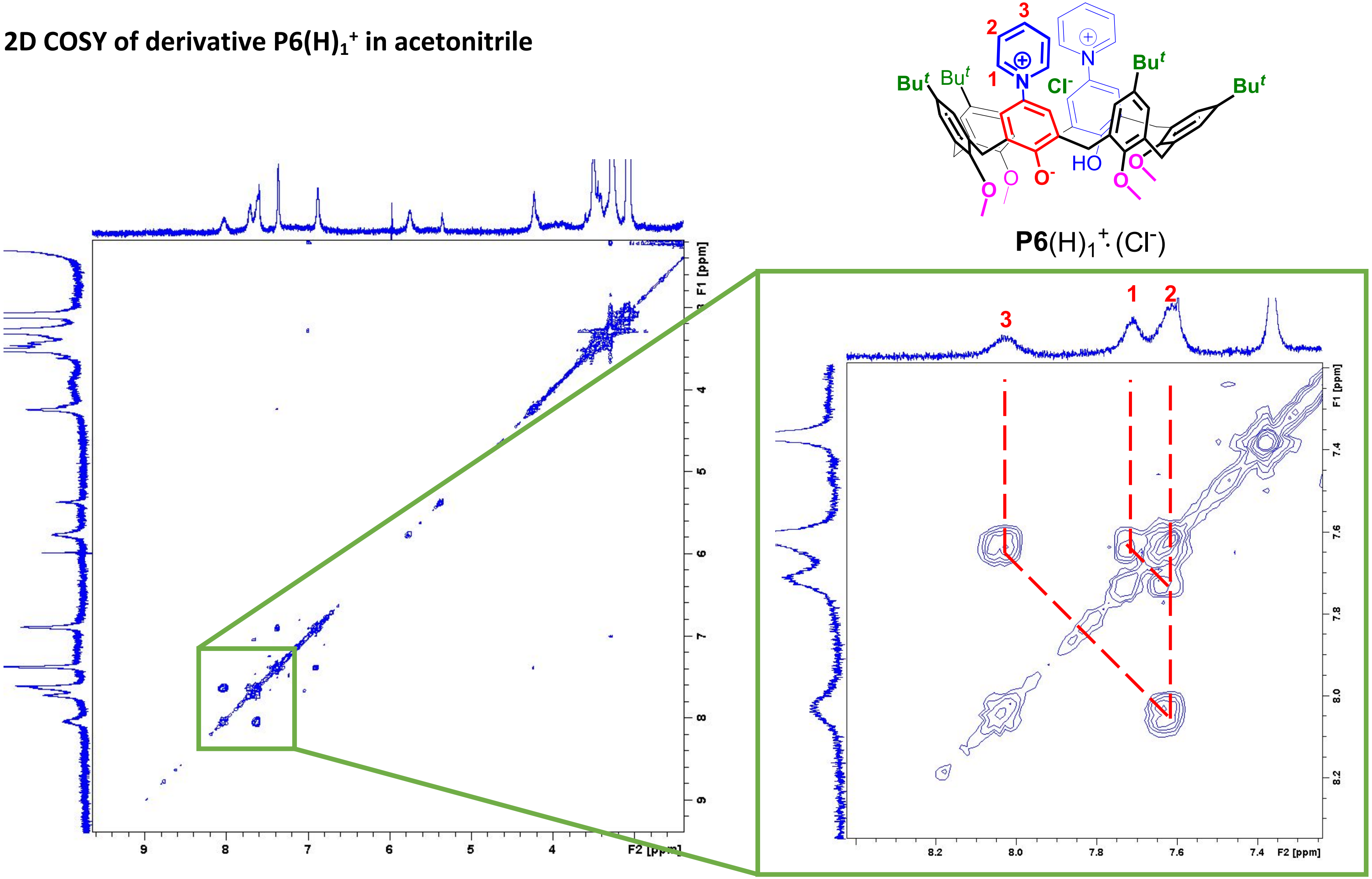

Figure S47. 2D COSY spectrum of derivative **P6**(H)$^+$·(Cl$^-$) (400 MHz, CD_3CN, 298 K).

^{1}H NMR spectra of equimolar mixtures of P6(H)$_1^+$/LiI and P6(H)$_1^+$/NaI

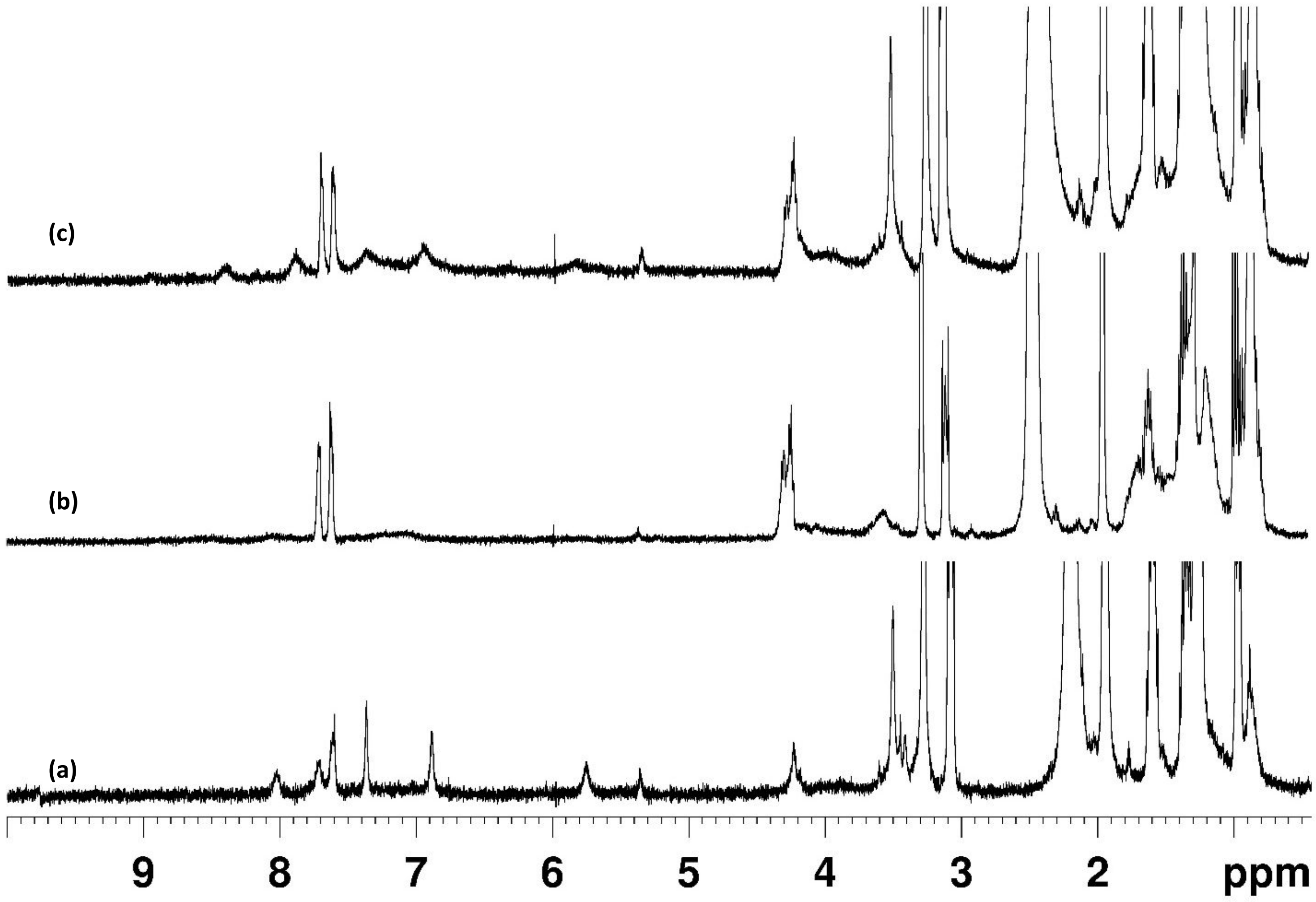

Figure S48. (a) ^{1}H NMR spectrum (400 MHz, CD_3CN, 298 K) of: **P6**(H)$_1^+$; (b) equimolar mixture of **P6**(H)$_1^+$ and LiI; (c) equimolar mixture of **P6**(H)$_1^+$ and NaI

Determination of association constants for the formation of Li^+@P6(H)$_1^+$ ·(Cl^-)·(I^-) and Na^+@P6(H)$_1^+$ ·(Cl^-)·(I^-) complexes by UV/Vis titrations using Benesi-Hildebrand equation

Association constants of complexes Li^+@**P6**(H)$_1^+$ ·(Cl^-)·(I^-) and Na^+@**P6**(H)$_1^+$ ·(Cl^-)·(I^-) were calculated according to the Benesi-Hildebrand equation.[7] Ka was calculated following the equation reported below.

$$\frac{1}{(A_0 - A)} = \frac{1}{K_a(A_0 - A_{max})[M^+]} + \frac{1}{A_0 - A_{max}}$$

Here A_0 is the absorbance of host in the absence of guest, A is the absorbance recorded in the presence of added guest, A_{max} is absorbance in presence of added $[M^+]_{max}$ and K_a is the association constant, where $[M^+]$ is $[Li^+]$ and/or $[Na^+]$. The association constant (Ka) could be determined from the slope of the straight line of the plot of $1/(A_0\text{-}A)$ against $1/[M^+]$. Errors < 15 % calculated as mean values of three measures.

- For Li^+@**P6**(H)$_1^+$ ·(Cl^-)·(I^-), $K_a = 1.7 \pm 0.2 \times 10^3$ M^{-1} (Figures S49 and S50).
- For Na^+@**P6**(H)$_1^+$ ·(Cl^-)·(I^-), $K_a = 6.5 \pm 0.2 \times 10^2$ M^{-1} (Figures S51 and S52).

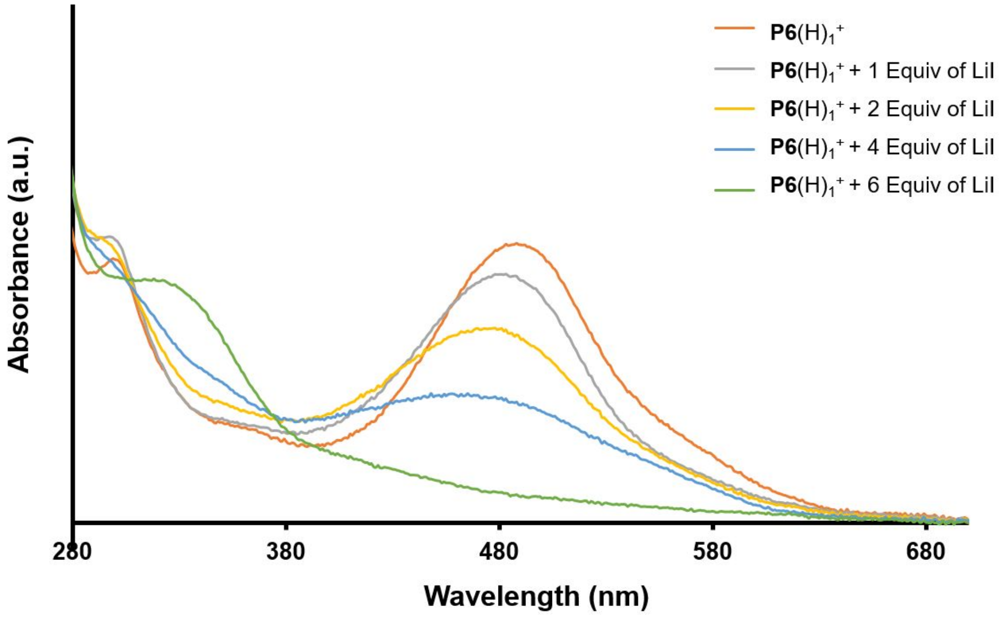

Figure S49. UV/Vis titration of **P6**(H)$_1^+$ with Li^+ as iodide salt (1-6 equiv) in acetonitrile at 25 °C.

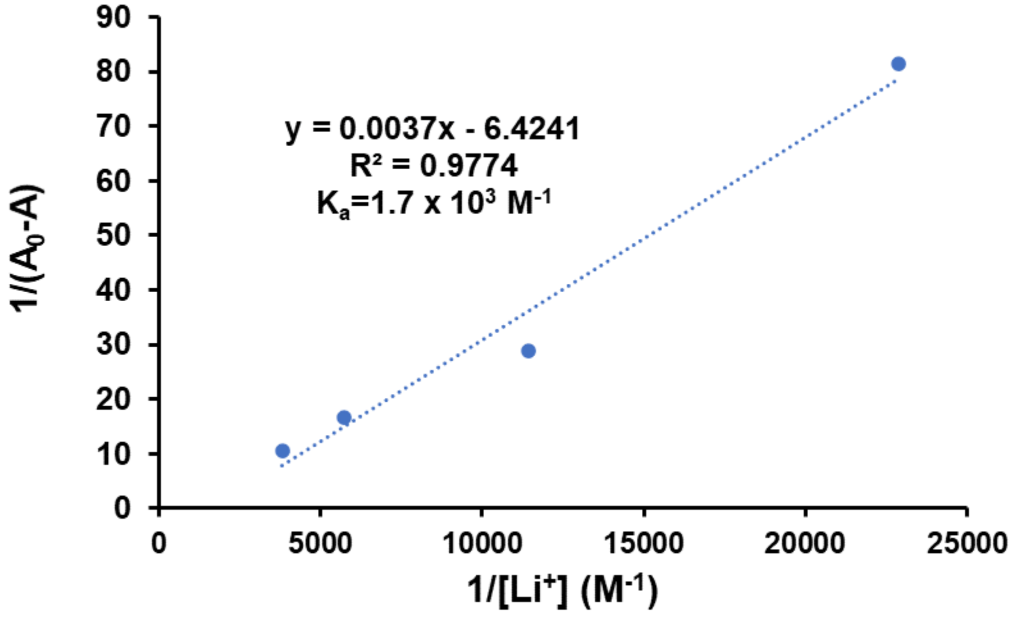

Figure S50. Benesi-Hildebrand plot from UV/vis titration data of **P6**(H)$_1^+$ with Li^+in acetonitrile at 25 °C; K_a=1.7±0.2 x 10^3 M^{-1}.

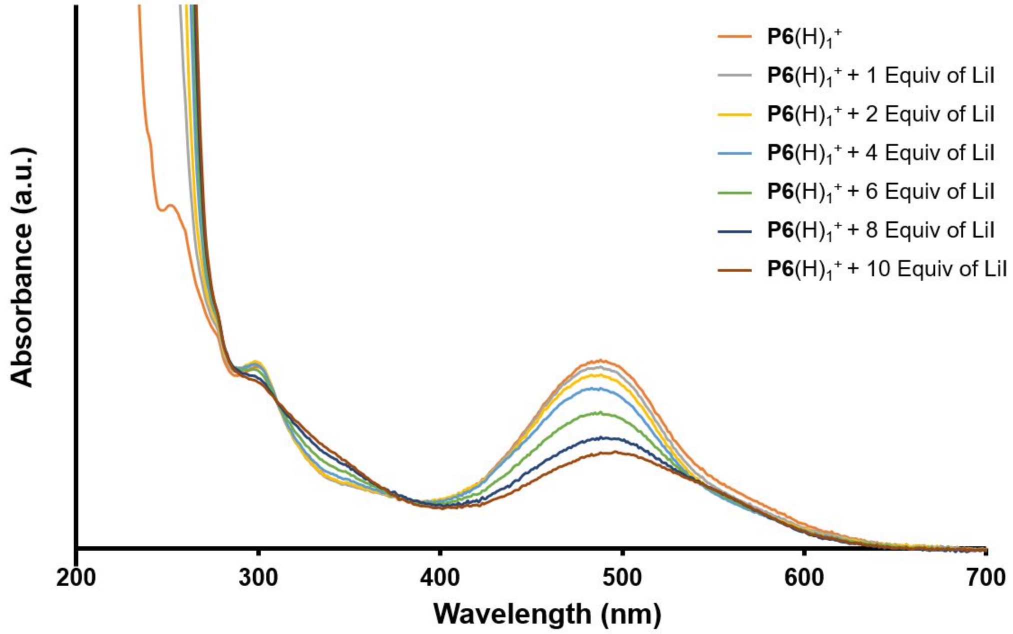

Figure S51. UV/Vis titration of **P6**$(H)_1^+$ with Na^+ as iodide salt (1-10 equiv) in acetonitrile at 25 °C.

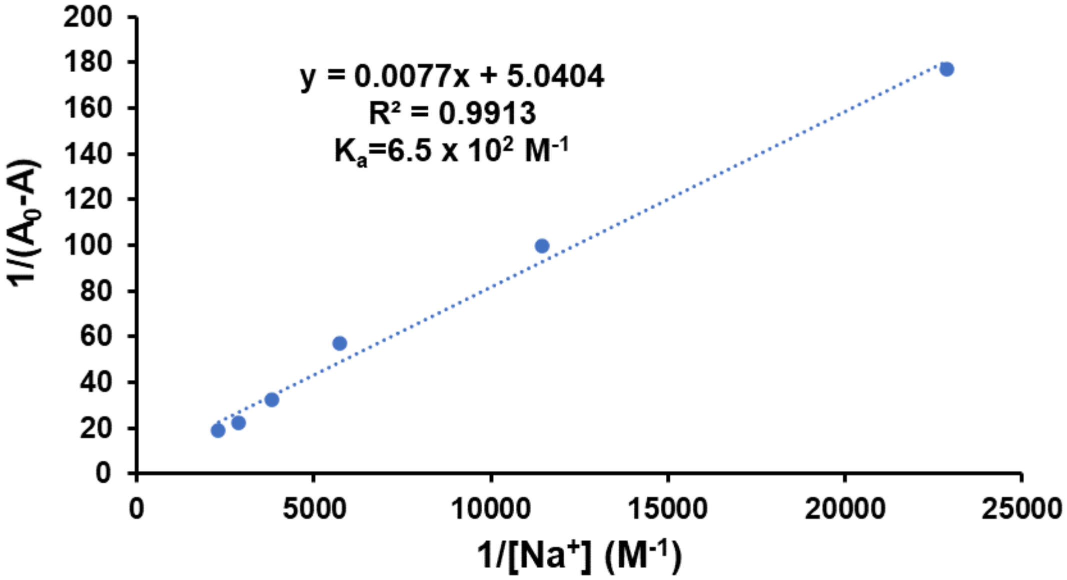

Figure S52. Benesi-Hildebrand plot from UV/Vis titration data of **P6**$(H)_1^+$ with Na^+ in acetonitrile at 25 °C; K_a=6.5±0.2 x 10^2 M^{-1}.

References

1. Krieger, E.; Vriend, G. YASARA View—Molecular Graphics for All Devices—from Smartphones to Workstations. *Bioinform.* **2014**, *30*, 2981–2982.

2. Salomon-Ferrer, R.; Case, D. A.; Walker, R. C. An Overview of the Amber Biomolecular Simulation Package: Amber Biomolecular Simulation Package. *WIREs Comput Mol Sci* **2013**, *3*, 198–210.
3. Mennucci, B. Polarizable Continuum Model. *WIREs Comput Mol Sci* **2012**, *2*, 386–404.
4. Yanai, T.; Tew, D. P.; Handy, N. C. A New Hybrid Exchange–Correlation Functional Using the Coulomb-Attenuating Method (CAM-B3LYP). *Chem. Phys. Lett.* **2004**, *393*, 51–57.
5. Iuliano, V.; Talotta, C.; Gaeta, C.; Soriente, A.; De Rosa, M.; Geremia, S.; Hickey, N.; Mennucci, B.; Neri, P. Negative Solvatochromism in a *N* -Linked *p* -Pyridiniumcalix[4]Arene Derivative. *Org. Lett.* **2019**, *21*, 2704–2707.
6. Gaussian 16, Revision A.02, Frisch, M. J.; Trucks, G. W.; Schlegel, H. B.; Scuseria, G. E.; Robb, M. A.; Cheeseman, J. R.; Scalmani, G.; Barone, V.; Petersson, G. A.; Nakatsuji, H.; Li, X.; Caricato, M.; Marenich, A. V.; Bloino, J.; Janesko, B. G.; Gomperts, R.; Mennucci, B.; Hratchian, H. P.; Ortiz, J. V.; Izmaylov, A. F.; Sonnenberg, J. L.; Williams-Young, D.; Ding, F.; Lipparini, F.; Egidi, F.; Goings, J.; Peng, B.; Petrone, A.; Henderson, T.; Ranasinghe, D.; Zakrzewski, V. G.; Gao, J.; Rega, N.; Zheng, G.; Liang, W.; Hada, M.; Ehara, M.; Toyota, K.; Fukuda, R.; Hasegawa, J.; Ishida, M.; Nakajima, T.; Honda, Y.; Kitao, O.; Nakai, H.; Vreven, T.; Throssell, K.; Montgomery, J. A., Jr.; Peralta, J. E.; Ogliaro, F.; Bearpark, M. J.; Heyd, J. J.; Brothers, E. N.; Kudin, K. N.; Staroverov, V. N.; Keith, T. A.; Kobayashi, R.; Normand, J.; Raghavachari, K.; Rendell, A. P.; Burant, J. C.; Iyengar, S. S.; Tomasi, J.; Cossi, M.; Millam, J. M.; Klene, M.; Adamo, C.; Cammi, R.; Ochterski, J. W.; Martin, R. L.; Morokuma, K.; Farkas, O.; Foresman, J. B.; Fox, D. J. Gaussian, Inc., Wallingford CT, 2016.
7. (a) Benesi, H. A.; Hildebrand, J. H. A Spectrophotometric Investigation of the Interaction of Iodine with Aromatic Hydrocarbons. *J. Am. Chem. Soc.* **1949**, *71*, 2703–2707; (b) Goswami, S.; Sen, D.; Das, N. K.; Fun, H.-K.; Quah, C. K. A New Rhodamine Based Colorimetric 'off–on' Fluorescence Sensor Selective for Pd^{2+} along with the First Bound X-Ray Crystal Structure. *Chem. Commun.* **2011**, *47*, 9101-9103. (c) Aich, K.; Goswami, S.; Das, S.; Mukhopadhyay, C. D. A New ICT and CHEF Based Visible Light Excitable Fluorescent Probe Easily Detects in Vivo Zn^{2+}. *RSC Adv.* **2015**, *5*, 31189–31194.
